# Highest ocean heat in four centuries places Great Barrier Reef in danger

**DOI:** 10.1038/s41586-024-07672-x

**Published:** 2024-08-07

**Authors:** Benjamin J. Henley, Helen V. McGregor, Andrew D. King, Ove Hoegh-Guldberg, Ariella K. Arzey, David J. Karoly, Janice M. Lough, Thomas M. DeCarlo, Braddock K. Linsley

**Affiliations:** 1 Environmental Futures, School of Earth, Atmospheric and Life Sciences, University of Wollongong, Wollongong, New South Wales Australia; 2 Securing Antarctica’s Environmental Future, University of Wollongong, Wollongong, New South Wales Australia; 3 School of Agriculture, Food and Ecosystem Sciences, University of Melbourne, Parkville, Victoria Australia; 4 School of Geography, Earth and Atmospheric Sciences, University of Melbourne, Parkville, Victoria Australia; 5 ARC Centre of Excellence for Climate Extremes, University of Melbourne, Parkville, Victoria Australia; 6 School of the Environment, The University of Queensland, Brisbane, Queensland Australia; 7 Australian Institute of Marine Science, Townsville, Queensland Australia; 8 ARC Centre of Excellence for Coral Reef Studies and School of Earth Sciences, University of Western Australia, Crawley, Western Australia Australia; 9 Department of Earth and Environmental Sciences, Tulane University, New Orleans, LA USA; 10 Lamont-Doherty Earth Observatory of Columbia University, Palisades, NY USA

**Keywords:** Climate change, Palaeoclimate, Environmental impact

## Abstract

Mass coral bleaching on the Great Barrier Reef (GBR) in Australia between 2016 and 2024 was driven by high sea surface temperatures (SST)^1^. The likelihood of temperature-induced bleaching is a key determinant for the future threat status of the GBR^2^, but the long-term context of recent temperatures in the region is unclear. Here we show that the January–March Coral Sea heat extremes in 2024, 2017 and 2020 (in order of descending mean SST anomalies) were the warmest in 400 years, exceeding the 95th-percentile uncertainty limit of our reconstructed pre-1900 maximum. The 2016, 2004 and 2022 events were the next warmest, exceeding the 90th-percentile limit. Climate model analysis confirms that human influence on the climate system is responsible for the rapid warming in recent decades. This attribution, together with the recent ocean temperature extremes, post-1900 warming trend and observed mass coral bleaching, shows that the existential threat to the GBR ecosystem from anthropogenic climate change is now realized. Without urgent intervention, the iconic GBR is at risk of experiencing temperatures conducive to near-annual coral bleaching^3^, with negative consequences for biodiversity and ecosystems services. A continuation on the current trajectory would further threaten the ecological function^4^ and outstanding universal value^5^ of one of Earth’s greatest natural wonders.

## Abstract (web summary)

High ocean temperatures that caused mass coral bleaching and mortality on the Great Barrier Reef in the past decade are the warmest in 400 years and are the result of human-caused climate change.

## Main

Like many coral reefs globally, the World Heritage-listed GBR in Australia is under threat^4,6^. Mass coral bleaching, declining calcification rates^5,7^, outbreaks of crown-of-thorns starfish (*Acanthaster* spp.)^8^, severe tropical cyclones^9^ and overfishing^10^ have placed compounding detrimental pressures on the reef ecosystem. Coral bleaching typically occurs when heat stress triggers the breakdown of the symbiosis between corals and their symbiotic dinoflagellates^11^. Although coral bleaching can occur locally as a result of low salinity, cold waters or pollution, regional and global mass bleaching events, in which the majority of corals in one or more regions bleach at once, are strongly associated with increasing SST linked to global warming^2^.

The first modern observations of mass coral bleaching on the GBR occurred in the 1980s, but these events were less widespread and generally less severe^3^ than the bleaching events in the twenty-first century^4^. Stress bands in coral skeletal cores have provided potential evidence for pre-1980s bleaching in the GBR and Coral Sea, such as during the 1877–78 El Niño^12^. However, stress bands are evident in relatively few cores before 1980 (ref. ^12^),  suggesting that severe mass bleaching did not occur in the 1800s and most of the 1900s.

As the oceans have warmed, however, mass coral bleaching events have become increasingly lethal to corals^4^. Coral bleaching on the GBR^1^ in 1998 coincided with a strong eastern-Pacific El Niño, and in 2002 with a weak El Niño. El Niño events can induce lower cloud cover and increased solar irradiance over the GBR^13^, increasing the risk of thermal stress and mass bleaching events^14^. In 2004, water temperatures were anomalously warm, and although bleaching occurred in the Coral Sea^15^, it was not widespread in the GBR, probably because there was reduced upwelling and an associated reduced influence of nutrients on symbiotic dinoflagellate expulsion^16^.

However, in the nine January–March periods from 2016 to 2024 (inclusive) there were five mass coral bleaching events on the GBR. Each was associated with high SSTs and affected large sections of the reef. GBR mass bleaching occurred in both 2016 and 2017, influenced by the presence of an El Niño event in 2016, and led to the death of at least 50% of shallow-water (depths of 5–10 m) reef-building corals^4^. Major bleaching events occurred again in quick succession in 2020 and 2022, with the accumulated heat stress for large sections of the GBR reaching levels conducive to widespread bleaching but lower levels of coral mortality^1^. The bleaching event in 2022 occurred, unusually, during a La Niña event, which is typically associated with cooler summer SSTs, higher than average rainfall and higher cloud cover on the GBR^1^. At the time of writing, researchers are assessing the impacts of the 2024 mass bleaching event.

The frequency of recent mass coral bleaching and mortality on the GBR is cause for concern. In 2021, the World Heritage Committee of the United Nations Educational, Scientific and Cultural Organization (UNESCO) drafted^17^ a decision to inscribe the GBR on the List of World Heritage in Danger, stating that the reef is “facing ascertained danger”, citing recent mass coral bleaching events and insufficient progress by the State Party (Australia) in countering climate change, improving water quality and land management issues. The committee’s adopted decisions^18^ have not included inscription of the ‘in danger’ status, but the draft inscription highlights the seriousness of the recent mass coral bleaching events. Authorities in Australia^5^ have noted that climate change and coral bleaching have deteriorated the integrity of the outstanding universal value of the GBR, a defining feature of its World Heritage status.

Although rapidly rising SSTs are attributed to human activities with virtual certainty^19^, understanding the multi-century SST history of the GBR is critical to understanding the influence of SST on mass coral bleaching and mortality in recent decades. Putting aside a problematic attempt to do this^20^, which was discredited^21,22^, knowledge of the long-term context for GBR SSTs comes primarily from two multi-century reconstructions based on the geochemistry of coral cores collected from the inner shelf^23^ and outer shelf^24^ (Flinders Reef) in the central GBR. These reconstructions showed that SSTs in the early 2000s were not unusually high relative to levels in the past three centuries, with five-year mean SSTs (and salinities) estimated to be higher in the 1700s than in the 1900s. However, these records were limited by their relatively coarse five-year sampling resolution and their most recent data point being from the early 2000s. After these studies were published, SSTs in the GBR have continued to rise. Updated analysis of coral data from Flinders Reef provides valuable improved temporal resolution^25^, but interpretations of these records remain limited spatially.

Here, we investigate the recent high SST events in the GBR region in the context of the past four centuries. We combine a network of 22 coral Sr/Ca and δ^18^O palaeothermometer series (Supplementary Tables 1 and 2) located in and near to the Coral Sea region to infer spatial mean SST anomalies (SSTAs) for January–March, the months when maximum SST and thermal bleaching are most likely to occur in the Coral Sea^16,26^, each year from 1618 to 1995 (Methods and Supplementary Information). Anthropogenic climate change began and proceeded entirely within the multi-century lives of some of these massive coral colonies, offering a continuous multi-century record covering the industrial era. We use this 1618–1995 reconstruction and the available 1900–2024 instrumental data to contextualize the modern trend and rank four centuries of January–March SSTAs with greater precision than was previously possible. We then assess the degree of human influence on ocean temperatures in the region using climate model simulations run both with and without anthropogenic forcing.

## The instrumental period (1900–present)

**Fig. 1:**
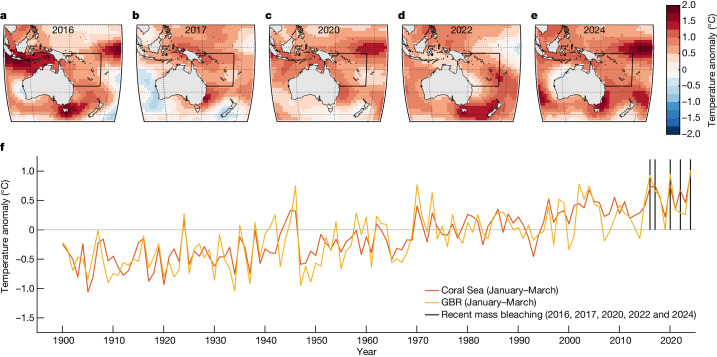
Widespread high SSTAs during GBR mass bleaching events. **a**–**e**, SSTAs (using ERSSTv5 data) for January–March in the Australasian region relative to the 1961–90 average for the five recent GBR mass coral bleaching years: 2016, 2017, 2020, 2022 and 2024. The black box shows the Coral Sea region (4° S–26° S, 142° E–174° E). **f**, Coral Sea and GBR mean SSTAs for 1900–2024 in January–March relative to the 1961–90 average. The black vertical lines indicate the five recent GBR mass coral bleaching years.

Mass coral bleaching on the GBR in 2016, 2017, 2020, 2022 and 2024 during January–March coincided with widespread warm SSTAs in the surrounding seas^1^, including the Coral Sea (Fig. 1a–e, using ERSSTv5 data^27^). The Coral Sea and GBR have experienced a strong warming trend since 1900 (Fig. 1f). January–March SSTAs averaged over the GBR are strongly correlated (*ρ* = 0.84, *P* ≪ 0.01) with those in the broader Coral Sea (Fig. 1f), including when the long-term warming trend is removed from both time series (*ρ* = 0.69, *P* < 0.01; Supplementary Fig. 4). Based on the strength of this correlation, we associate high January–March area-averaged Coral Sea SSTAs with increased thermal bleaching risk in the GBR.

Record temperatures were set in 2016 and 2017 in the Coral Sea, and in 2020 they peaked fractionally below the record high of 2017. The January–March of 2022 was another warm event, the fifth warmest on record at the time. Recent data (ERSSTv5) indicate that 2024 set a new record by a margin of more than 0.19 °C above the previous record for the region. The January–March mean SSTs averaged over the five mass bleaching years during the period 2016–2024 are 0.77 °C higher than the 1961–90 January–March averages in both the Coral Sea and the GBR. The multidecadal warming trend, extreme years and association between GBR and Coral Sea SSTs are similar for the HadISST^28^ gridded SST dataset, with some notable differences in the 1900–40 period (Supplementary Fig. 3). Furthermore, analysis of modern temperature-sensitive Sr/Ca series from GBR corals for 1900–2017 provides coherent independent evidence of statistically significant multi-decadal warming trends in January–March SSTs in the central and southern GBR (Supplementary Information section 4.2).

## A multi-century context (1618–present)

**Fig. 2:**
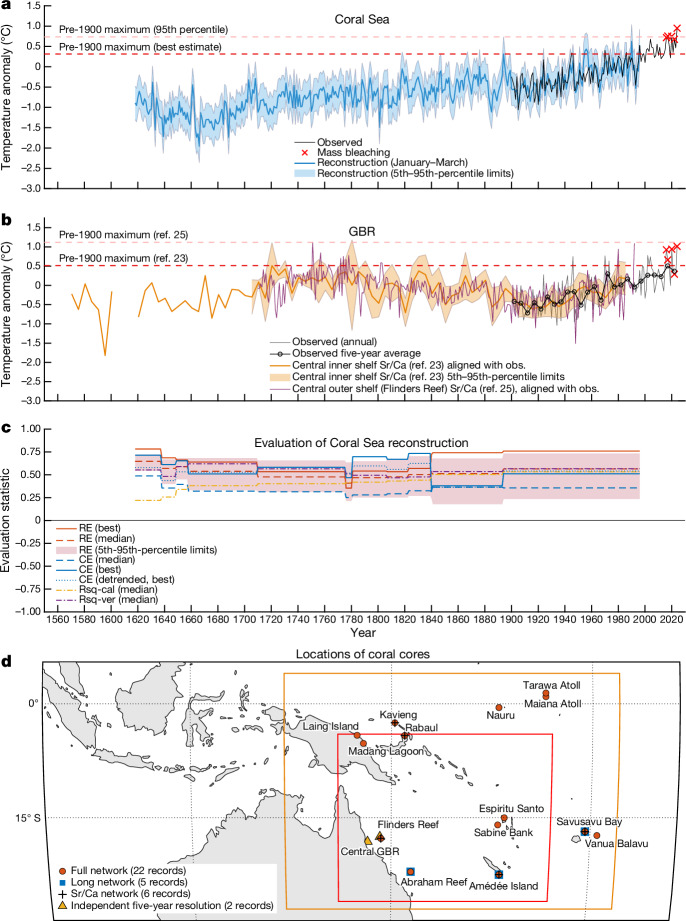
Multi-century reconstruction of January–March SSTAs. **a**, Reconstructed and observed mean January–March SSTAs in the Coral Sea for 1618–2024 relative to 1961–90. Dark blue, highest skill (maximum coefficient of efficiency) reconstruction with the full proxy network; light blue, 5th–95th-percentile reconstruction uncertainty; black, observed (ERSSTv5) data. Red crosses indicate the five recent mass bleaching events. Dashed lines indicate the best estimate (highest skill, red) and 95th-percentile (pink) uncertainty bound for the maximum pre-1900 January–March SSTA. **b**, Central GBR SSTA for the inner shelf^23^ in thick orange and outer shelf^25^ (Flinders Reef) in thin orange lines; these series are aligned here (see Methods) with modern observations of mean GBR SSTAs for January–March relative to 1961–90. Observed data are shown at annual (grey line) and five-year (black line with open circles, plotted at the centre of each five-year period and temporally aligned with the five-year coral series^23^) resolution. Dashed lines indicate best-estimate pre-1900 January–March maxima for refs. ^23^ (red) and ^25^ (pink). Orange shading indicates 5th–95th-percentile uncertainty bounds. Red crosses indicate the five recent mass bleaching events. **c**, Evaluation metrics for the Coral Sea reconstruction (Supplementary Information section 3.1); RE, reduction of error; CE, coefficient of efficiency; Rsq-cal, R-squared in the calibration period; Rsq-ver, R-squared in the verification (evaluation) period. **d**, Coral data locations relative to source data region (orange box) and Coral Sea region (red box). Coral proxy metadata are given in Supplementary Tables 1 and 2.

Reconstructing Coral Sea January–March SSTs from 1618 to 1995 extends the century-long instrumental record back in time by an additional three centuries (Fig. 2a and Methods). The reconstruction (calibrated to ERSSTv5) shows that multi-decadal SST variability was a persistent feature in the past. At the centennial timescale, there is relative stability before 1900, with the exception that cooler temperatures prevailed in the 1600s. Warming during the industrial era has been evident since the early 1900s (Fig. 2a). There is a warming trend for January–March of 0.09 °C per decade for 1900–2024 and 0.12 °C per decade for 1960–2024 (Fig. 1f) using ERSSTv5 data. Calibrating our reconstruction to HadISST1.1 yields similar results, with some differences in the degree of pre-1900 variability at both multi-decadal and centennial timescales (Supplementary Information section 5.2.6).

Our best-estimate (highest skill; Methods) annual-resolution Coral Sea reconstruction (Fig. 2a), using the full coral network calibrated to the ERSSTv5 instrumental data, indicates that the January–March mean SSTAs in 2016, 2017, 2020, 2022 and 2024 were, respectively, 1.50 °C, 1.54 °C, 1.53 °C, 1.46 °C and 1.73 °C above the 1618–1899 (hereafter ‘pre-1900’) reconstructed average. Using the same best-estimate reconstruction, Coral Sea January–March SSTs during these GBR mass bleaching years were five of the six warmest years the region has experienced in the past 400 years (Fig. 2a).

By comparing the recent warm events to the reconstruction’s uncertainty range (Methods), we quantify, using likelihood terminology consistent with recent reports from the Intergovernmental Panel on Climate Change^19^, that the recent heat extremes in 2017, 2020 and 2024 are ‘extremely likely’ (>95th percentile; Fig. 2a) to be higher than any January–March in the period 1618–1899. Furthermore, the heat extremes in 2016 and 2022 are (at least) ‘very likely’ (>90th percentile) to be above the pre-1900 maximum. We perform a series of tests that verify that our findings are not simply an artefact of the nature of the coral network itself (Supplementary Information section 5.2). In a network perturbation test, we generate 22 subsets of the reconstruction by adding proxy records incrementally in order from the highest to the lowest correlation with the target (Supplementary Information section 5.2.5). We confirm that 2017, 2020 and 2024 were ‘extremely likely’ (>95th percentile) to have been warmer than any year pre-1900 (using ERSSTv5 data) for all of these proxy subsets. Furthermore, in 20 of the 22 subsets, 2016 was also ‘extremely likely’ (>95th percentile), rather than ‘very likely’, to be warmer (2022 was ‘extremely likely’ in 14 of the 22 subsets). All our additional tests, including a reconstruction with only Sr/Ca coral data (thereby omitting the possibility of any non-temperature signal in δ^18^O_coral_ on the reconstruction), achieve high reconstruction skill and confirm the extraordinary nature of recent extreme temperatures in the multi-century context (Supplementary Information section 5.2). Analyses using HadISST1.1 generally show lower correlations with the coral data and reconstructions with slightly warmer regional SSTs before 1900, along with more-muted centennial and multi-decadal variability in the pre-instrumental period. Nevertheless, the HadISST1.1-calibrated reconstructions show that the recent thermal extremes are well above the best estimate (highest skill) of the pre-1900 maximum of reconstructed January–March SSTAs (Supplementary Fig. 42). Furthermore, lower SSTAs (in the HadISST1.1 data) relative to the previous three centuries (as in our reconstructions calibrated to HadISST1.1), coupled with the recently observed mass coral bleaching events, could indicate that long-lived corals have a greater sensitivity to warming than is currently recognized.

Reconstructed regional GBR SSTAs based on a five-year-resolution, multi-century coral δ^18^O record from the central inshore GBR^23^ (Fig. 2b) show similarly strong warming since 1900 but more multi-decadal-to-centennial variability than the Coral Sea reconstruction. Recent five-year mean January–March GBR SSTAs narrowly exceed the best estimate of the maximum pre-1900 five-year mean since the early 1600s (Fig. 2b). The averages for the five-year periods centred on 2018 and 2022 exceed the pre-1900 maximum by 0.11 °C and 0.06 °C, respectively. Results are similar using the five-year-resolution Flinders Reef (central outer shelf)^24^ record (Supplementary Fig. 24), although its interpretation is limited by the lack of uncertainty estimates available for that record. Our Coral Sea reconstruction incorporates an updated (annual resolution) record from Flinders Reef^25^, which indicates similar centennial trends (thin orange line in Fig. 2b) and shows that the recent high January–March SSTA events have approached the estimated local pre-1900 maximum SSTA. Although contiguous multi-century cores from within the GBR are limited in their spatial extent, twentieth-century warming is evident in these records.

**Fig. 3:**
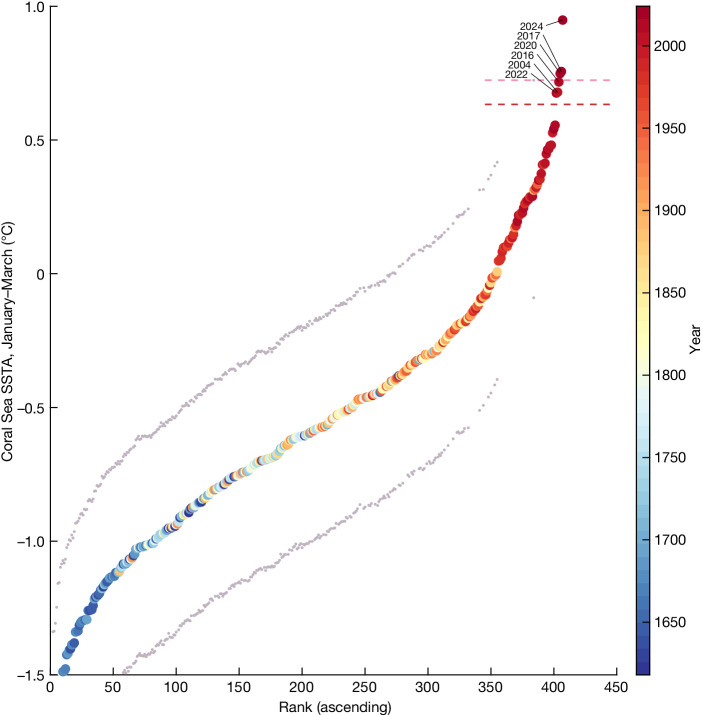
Exceptional nature of recent January–March Coral Sea surface temperatures. Ranked January–March SSTAs for 1618–2024 relative to 1961–90 (coloured circles) from the best-estimate (highest skill, full coral network) reconstruction (1618–1899) and instrumental (ERSSTv5) data (1900–2024). The year is indicated by the colour of the filled circles. The 5th–95th-percentile uncertainty bounds of the pre-1900 reconstructed SSTAs are shown by small grey dots. The year labels indicate the warmest six years on record, five of which were mass coral bleaching years on the GBR. The pink (upper) dashed line indicates the 95th-percentile uncertainty bound of the maximum pre-1900 reconstructed SSTA; the red (lower) dashed line indicates the 90th-percentile limit.

The extraordinary nature of the recent Coral Sea January–March SSTs in the context of the past 400 years is further illustrated by comparing the ranked temperature anomalies (Fig. 3) for the combined reconstructed and instrumental period from 1618–2024, incorporating reconstruction uncertainty (Methods). The mass coral bleaching years of 2016, 2017, 2020, 2022 and 2024, and the heat event of 2004, stand out as the warmest events across the whole 407-year record. The warmest three years (2024, 2017 and 2020) exceed the upper uncertainty bound (95th percentile) of the warmest reconstructed January–March in the pre-1900 period (pink (upper) dashed line in Fig. 3); 2016, 2004 and 2022 exceed the 90th percentile bound (red (lower) dashed line in Fig. 3). The warming trend is clear in the association between the ascending rank of the temperature anomalies and the year (shown as the colour of the filled circles in Fig. 3). Despite high interannual variability, 78 of the warmest 100 January–March periods between 1618 and 2024 occurred after 1900, and the 23 warmest all occur after 1900. The warmest 20 January–March periods all occur after 1950, coinciding with accelerated global warming.

## Assessing anthropogenic influence

**Fig. 4:**
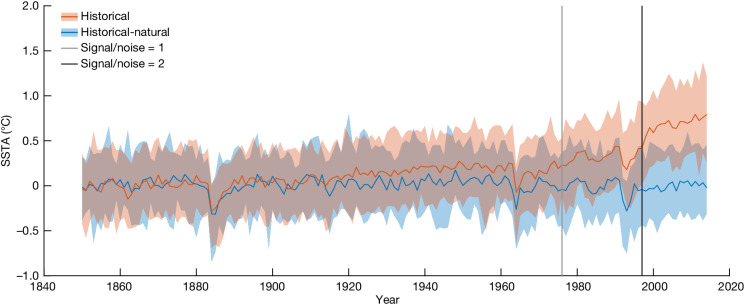
Climate change is driving the rise in Coral Sea January–March SSTAs. Climate-model simulations of Coral Sea January–March SSTAs relative to the 1850–1900 average for the period 1850–2014, for models within the ‘likely’ range for their transient climate response^31^. The blue line (median) and light blue shading (5th–95th-percentile limits) are from the ‘historical-natural’ climate model simulations (no anthropogenic climate forcing); the red line and light red shading are from the ‘historical’ simulations (anthropogenic influences on the climate included) using the same set of climate models. The climate-model-derived time of emergence of anthropogenic climate change, shown by the grey and black vertical lines (1976 and 1997), is when the ratio of the climate change signal to the standard deviation of noise/variability^32^ across model ensemble members first rises above 1 and 2, respectively. All models are represented equally in the model ensemble.

Using climate model simulations from the most recent (sixth) phase of the Coupled Model Intercomparison Project^29^ (CMIP6), we assess the human influence on January–March SSTAs in the Coral Sea. The model simulations are from two experiments in the Detection and Attribution Model Intercomparison Project (DAMIP)^30^. The first set of simulations represents historical climate conditions, including both the natural and human influences on the climate system over the 1850–2014 period (‘historical’; red in Fig. 4). The second experiment is a counterfactual climate that spans the same period and uses the same models but includes only natural influences on the climate, omitting all human influences (‘historical-natural’; blue in Fig. 4). The historical experiment includes anthropogenic emissions of greenhouse gases and aerosols, stratospheric ozone changes and anthropogenic land-use changes; the historical-natural experiment does not. Variations in natural climate forcings, such as from volcanic eruptions and solar variability, are incorporated in both experiments. We include models that have a transient climate response (the global mean surface-temperature anomaly at the time of a doubling of atmospheric CO_2_ concentration) in the range 1.4–2.2 °C, which is deemed ‘likely’ by the science community^31^ (Methods and Supplementary Information).

It is only with the incorporation of anthropogenic influences on the climate that the model simulations capture the modern-era warming of the Coral Sea January–March SSTA (Fig. 4). The median of the historical simulations has statistically significant warming trends of 0.05 °C, 0.10 °C and 0.15 °C per decade for the periods from 1900, 1950 and 1970 to 2014, respectively; the equivalent historical-natural trends are smaller in magnitude than ±0.01 °C per decade. To further explore the centennial-scale trends, we use a bootstrap ensemble (Methods) of the two sets of 165-year simulations from 1850–2014. We found that 100% of the historical bootstrap ensemble has statistically significant positive trends (Methods) for 1900–2014, but this value is 0% for the historical-natural ensemble. The observed (ERSSTv5) mean SSTA for 2016–2024 of 0.60 °C relative to 1961–90 is warmer than any nine-year sequence in the 7,095 simulated years in the historical-natural experiments from models with transient climate responses in the ‘likely’ range^31^.

We also use the simulations to estimate the time of emergence of the anthropogenic influence on January–March Coral Sea SSTAs above the natural background variability. The anthropogenic warming signal^32^ increases from near zero in 1900 to around 0.5 standard deviations of the variability (‘noise’) in 1960. The climate change signal-to-noise ratio then increases rapidly from 1960 to 2014, exceeding 1.0 in 1976, 2.0 in 1997 and around 2.8 by 2014, the end of these simulations (Fig. 4, Methods and Supplementary Fig. 50). Anthropogenic impacts on the climate are virtually certain to be the primary driver of this long-term warming in the Coral Sea.

## Discussion

Previously, our knowledge of the SST history of the GBR and the Coral Sea region has been highly dependent on instrumental observations, with the exception of the five-year-resolution multi-century coral Sr/Ca and U/Ca SST reconstructions from the two point locations in the central GBR^23,24^, an update at one of these locations^25^, seasonal resolution ‘floating’ (in time) chronologies from the GBR in the Holocene^33,34^ and point SST estimates further back in time^35^. Thus, the context of recent warming trends in the Coral Sea and GBR and their relation to natural variability on decadal to centennial timescales is largely unknown without reconstructions such as the one we developed here.

Our coral proxy network is located mostly beyond the GBR, in the Coral Sea, and some series are located outside the Coral Sea region (Fig. 2d). The selection of the Coral Sea as a study region allowed for a larger sample of contributing coral proxy data than exists for the GBR. However, coral bleaching on the GBR can be influenced by factors other than large-scale SST, including local oceanic and atmospheric dynamics that can modulate the occurrence and severity of thermal bleaching and mortality events^13^. Nonetheless, warming of seasonal SSTs over the larger Coral Sea region is likely to prime the background state and increase the likelihood of smaller spatio-temporal-scale heat anomalies. Furthermore, where we use only the five-year resolution series directly from the GBR to reconstruct GBR SSTAs, we draw similar conclusions about the long-term trajectory of SSTAs as for our full coral network (Fig. 2b and Supplementary Fig. 24). Furthermore, short modern coral series from within the GBR, analysed in this study, document a multi-decadal warming signal that is coherent with instrumental data (Supplementary Figs. 29 and 30). Nonetheless, additional high-resolution, multi-century, temperature-sensitive coral geochemical series from within the GBR would help unravel the local and remote ocean–atmosphere contributions to past bleaching events and reduce uncertainties.

The focus on the larger Coral Sea study region also takes advantage of the global modelling efforts of CMIP6. The large number of ensemble members available for CMIP6 means that greater climate model diversity, and therefore greater certainty in our attribution analysis, is possible compared with most single model analyses. There is also a methodological benefit in having high replication of the same experiments run with multiple climate models. However, coarse-resolution global-scale models do not accurately simulate smaller-scale processes, such as inshore currents and mesoscale eddies in the Coral Sea or the Gulf of Carpentaria, which probably affect local surface temperatures and variations in nutrient upwelling in the GBR^36,37^. Upwelling on the GBR is linked to the strength of the East Australian Current^16^, the southward branch of the South Pacific subtropical gyre. The CMIP-scale models we use do capture these gyre dynamics. The models show that the East Australian Current is expected to increase in strength as the climate continues to warm through this century^38^, and this may lead to more nutrient inputs that can exacerbate coral sensitivity to rising heat stress^39,40^. As well as focusing our model analysis on the larger Coral Sea region, we use a three-month time step. In doing so, we minimize the impact of model spatio-temporal resolution on our inferences about the role of anthropogenic greenhouse-gas emissions on the SST conditions that give rise to GBR mass bleaching.

### Remaining uncertainties

We present analyses and interpretations that are as robust as possible given currently available data and methods. However, several sources of remaining uncertainty mean that future reconstructions of past Coral Sea and GBR SSTs could differ from those presented here. Although bias corrections are applied to observational SST datasets such as ERSST and HadISST, these datasets probably retain biases, especially for the period during and before 1945 (ref. ^41^), and these may not be fully accounted for in the uncertainty estimates^42^. Because our reconstructions are calibrated directly to these datasets, future observational-bias corrections are likely to improve proxy-based reconstructions.

Reconstructions of SST that use coral δ^18^O records may be susceptible to the influence of changes in the coral δ^18^O–SST relationship on time periods longer than the instrumental training period, along with non-SST changes in the δ^18^O of seawater, which can covary with salinity. As such, new coral records of temperature-sensitive trace-element ratios such as Sr/Ca, Li/Mg or U/Ca may prove influential in future efforts to distinguish between changes in past temperature and hydroclimate. Owing to the limited availability of multi-century coral data from within the GBR itself, the reconstructed low-frequency variability of GBR SSTs in recent centuries is likely to change as more temperature proxy data become available. It is also likely that new sub-annual resolution records would aid in removing potential signal damping or bias from our use of some annual-resolution records to reconstruct seasonal SSTAs.

### Ecological consequences

With global warming of 0.8–1.1 °C above pre-industrial levels^19^ there has been a marked increase in mass coral bleaching globally^43^. Even limiting global warming to the Paris Agreement’s ambitious 1.5 °C level would be likely to lead to the loss of 70–90% of corals that are on reefs today^44^. If all current international mitigation commitments are implemented, global mean surface temperature is still estimated to increase in the coming decades, with estimates varying between 1.9 °C (ref. ^45^) and 3.2 °C (ref. ^46^) above pre-industrial levels by the end of this century. Global warming above 2 °C would have disastrous consequences for coral ecosystems^19,44^ and the hundreds of millions of people who currently depend on them.

Coral reefs of the future, if they can persist, are likely to have a different community structure to those in the recent past, probably one with much less diversity in coral species^4^. This is because mass bleaching events have a differential impact on different coral species. For example, fast-growing branching and tabulate corals are affected more than slower-growing massive species because they have different thermal tolerance^4^. The simplification of reef structures will have adverse impacts on the many thousands of species that rely on the complex three-dimensional structure of reefs^4^. Therefore, even with an ambitious long-term international mitigation goal, the ecological function^4^ of the GBR is likely to deteriorate further^5^ before it stabilizes.

Coral adaptation and acclimatization may be the only realistic prospect for the conservation of some parts of the GBR this century. However, although adaptation opportunities may be plausible to some extent^47^, they are no panacea because evolutionary changes to fundamental variables such as temperature take decades, if not centuries, to occur, especially in long-lived species such as reef-building corals^48^. There is currently no clear evidence of the real-time evolution of thermally tolerant corals^48^. Most rapid changes depend on a history of exposure to key genetic types and extremes, and there are limitations to genetic adaptation that prevent species-level adaptation to environments outside of their ecological and evolutionary history^19^. Model projections also indicate that rates of coral adaptation are too slow to keep pace with global warming^49^. In a rapidly warming world, the temperature conditions that give rise to mass coral bleaching events are likely to soon become commonplace. So, although we may see some resilience of coral to future marine heat events through acclimatization, thermal refugia are likely to be overwhelmed^50^. Global warming of more than 1.5 °C above pre-industrial levels will probably be catastrophic for coral reefs^44^.

### Conclusion

Our new multi-century reconstruction illustrates the exceptional nature of ocean surface warming in the Coral Sea today and the resulting existential risk for the reef-building corals that are the backbone of the GBR. The reconstruction shows that SSTs were relatively cool and stable for hundreds of years, and that recent January–March ocean surface heat in the Coral Sea is unprecedented in at least the past 400 years. The coral colonies and reefs that have lived through the past several centuries, and that yielded the valuable Sr/Ca and δ^18^O data on which our reconstruction is based, are themselves under serious threat. Our analysis of climate-model simulations confirms that human influence is the driver of recent January–March Coral Sea surface warming. Together, the evidence presented in our study indicates that the GBR is in danger. Given this, it is conceivable that UNESCO may in the future reconsider its determination that the iconic GBR is not in danger. In the absence of rapid, coordinated and ambitious global action to combat climate change, we will likely be witness to the demise of one of Earth’s great natural wonders.

## Methods

### Instrumental observations

The Coral Sea and GBR area-averaged monthly SSTAs relative to 1961–90 for January–March are obtained from version 5 of the Extended Reconstructed Sea Surface Temperature dataset (ERSSTv5)^27^. We compare our results using ERSSTv5 with those generated using the Hadley Centre Sea Ice and Sea Surface Temperature dataset (HadISST1.1)^28^. We use only post-1900 instrumental SST observations here. Although gridded datasets have some coverage before 1900, ship-derived temperature data in the region for that period are too sparse to be reliable for calibrating our reconstruction (Supplementary Information section 1.2). The regional mean for the GBR is computed using the seven grid-cell locations used by the Australian Bureau of Meteorology (Supplementary Information section 1.1). We define the Coral Sea region as the ocean areas inside 4° S–26° S, 142° E–174° E.

### Coral-derived temperature proxy data

We use a network of 22 published and publicly available sub-annual and annual resolution temperature-sensitive coral geochemical series (proxies; Fig. 2d, Supplementary Tables 1 and 2, and Supplementary Fig. 5a–v) from the western tropical Pacific in our source data region (4° N–27° S, 134° E–184° E) that cover at least the period from 1900 to 1995. Of these 22 series, 16 are δ^18^O, which are in per mil (‰) notation relative to Vienna PeeDee Belemnite (VPDB)^51^; the remaining six are Sr/Ca series. The coral data are used as predictors in the reconstruction of January–March mean SSTAs in the Coral Sea region. We apply the inverse Rosenblatt transformation^52,53^ to the coral data to ensure that our reconstruction predictors are normally distributed. Sub-annually resolved series are converted to the annual time step by averaging across the November–April window. This maximizes the detection of the summer peak values, allowing for some inaccuracy in sub-annual dating and the timing of coral skeleton deposition^54,55^. A small fraction (less than 0.8%) of missing data is infilled using the regularized expectation maximization (RegEM) algorithm^56^ (Supplementary Information section 2.3), after which the proxy series are standardized such that each has a mean of zero and a standard deviation of one over their common 1900–1995 period.

### Reconstruction method

To produce our Coral Sea reconstruction, we use nested principal component regression^57^ (PCR), in which the principal components of the network of 22 coral proxies are used as regressors against the target-region January–March SSTA relative to the 1961–90 average. We perform the reconstructions separately for each nest of proxies, where a nest is a set of proxies that cover the same time period. The longest nest dates back to 1618, when at least two series are available. The nests allow for the use of all coral proxies over the full time period of their coverage. The 96-year portion of the instrumental period (1900–1995) that overlaps with the reconstruction period is used for calibration and evaluation (or equivalently, verification) against observations. We reconstruct regional SSTAs from the principal components of the coral network of δ^18^O and Sr/Ca data, rather than their local SST calibrations, to minimize the number of computational steps and to aid in representing the full reconstruction uncertainty.

Principal component analysis (PCA) is used to reduce the dimensionality of the proxy matrix, as follows. Let *P*(*t*,*r*) denote the palaeoclimate-data matrix during the time period *t* = 1,...,*n* at an annual time step for proxy series *r* = 1,...,*p*. PCA is undertaken on this matrix during the calibration period, *P*_cal_. We obtain the principal component coefficients matrix *P*_coeff_(*r*,*e*) for principal components *e* = 1,...,*n*_PC_ and principal component scores *P*_score_(*t*,*e*), which are representations of the input matrix *P*_cal_ in the principal component space. *P*_score_ is truncated to include *n*_PC,use_ principal components to form ${P}_{{\rm{score}}}^{{\prime} }$ such that the variance of the proxy network explained by the *n*_PC,use_ principal components is greater than ${\sigma }_{{\rm{expl}}}^{2}$ (which we set to 95%). Reconstruction tests in which ${\sigma }_{{\rm{expl}}}^{2}$ is varied from 70% to 95% show that our results are not strongly sensitive to this choice, and tests based on lag-one autoregressive noise for ${\sigma }_{{\rm{expl}}}^{2}$ from 50% to 99% further support this choice (Supplementary Information section 3.2). These principal components are used as predictors against which the Coral Sea January–March instrumental SSTAs are regressed. We regress the standardized SSTA target data during the calibration period, *I*_cal_, against the retained principal components of the predictor data, ${P}_{{\rm{score}}}^{{\prime} }$:

$${I}_{{\rm{cal}}}=\mathop{\sum }\limits_{e=1}^{{n}_{{\rm{PC}},{\rm{use}}}}{P}_{{\rm{score}}}^{{\prime} }\left(t,e\right)\times \,{\gamma }_{e}+\,{\varepsilon }_{t}$$

Thus, we obtain *n*_PC,use_ estimates of the regression coefficients *γ*_*e*_ with gaussian error term *ε*_*t*_ ~ *N*(0,${\sigma }_{N}^{2}$). The principal components are extended back into the pre-instrumental period by multiplying the entire proxy matrix *P*(*t*,*p*) with the truncated principal component coefficient matrix ${P}_{{\rm{coeff}}}^{{\prime} }$(*t*,*e*) to obtain ${Q}_{{\rm{coeff}}}^{{\prime} }$:

$${Q}_{{\rm{coeff}}}^{{\prime} }(t,p)=P(t,p)\times \,{P}_{{\rm{coeff}}}^{{\prime} }(t,e).$$

The reconstruction proceeds with the fitted regression coefficients *γ*_*e*_ and extended coefficient matrix ${Q}_{{\rm{coeff}}}^{{\prime} }$ to obtain a reconstruction time series *R*_*m*_(*t*) for a given nest of proxy series

$${R}_{m}(t)=\mathop{\sum }\limits_{e=1}^{{n}_{{\rm{eof}}}}{Q}_{{\rm{coeff}}}^{{\prime} }(t,e)\,\times \,{\gamma }_{e}+\,{\varepsilon }_{t}.$$

The standardized reconstruction *R*_*m*_(*t*) is then calibrated to the instrumental data such that the standard deviation and mean of the reconstruction and target during the calibration interval are equal. As well as obtaining reconstructions for each nest of available proxies, we compute stitched reconstructions *S*_*c*_(*t*) for each calibration period *c*, which include at each time step the reconstructed data for the proxy nest with maximum coefficient of efficiency^58,59^ (Supplementary Information section 3.1). This procedure is performed for contiguous calibration intervals between 60 and 80 years duration between 1900 and 1995, with interval width and location increments of two years, reserving the remaining data in the overlapping period for independent evaluation, and for all proxy nests. The reconstruction error is modelled with a lag-one autoregressive process fitted to the residuals. We evaluate the capacity of our reconstruction method to achieve spurious skill from overfitting by performing a test in which we replace the coral data with synthetic noise (Supplementary Information section 3.2i). We find that reconstructions based on synthetic noise achieve extremely low or zero skill and as more noise principal components are included in the regression, the evaluation metrics indicate declining skill. Our reconstruction and evaluation methods therefore guard against the potential for spurious skill.

### Pseudo-proxy reconstructions

Our reconstruction method is further evaluated by using a pseudo-proxy modelling approach based on the Community Earth System Model (CESM) Last Millennium Experiment (LME)^60^, for which there are 13 full-forcing ensemble members covering the period 850–2005. We use the pseudo-proxy reconstructions to evaluate our reconstruction method and coral network in a fully coupled climate-model environment. We form pseudo-proxies by extracting from each LME ensemble member the SST and sea surface salinity (SSS) from the 1.5° × 1.5° grid cell located nearest to our coral data. We then apply proxy system models in the form of linear regression models, basing δ^18^O on both SST and SSS, and Sr/Ca on SST only (Supplementary Information section 3.3). We set the spatial and temporal availability of the pseudo-coral network to match that of the coral network. We then apply our PCR reconstruction and evaluation procedure to the pseudo-proxy network, taking advantage of the availability of the modelled Coral Sea SSTA data across the multi-century period of 1618–2005, which allows for the evaluation of the pseudo-proxy reconstruction over this entire time period. We first test our method using a ‘perfect proxy’ approach (with no proxy measurement error) before superimposing synthetic noise on the pseudo-proxy time series, evaluating our methodology at two separate levels of measurement error, quantified by signal-to-noise ratios of 1.0 and 4.0. The evaluation metrics for these tests indicate that our coral network and reconstruction method obtain skilful reconstructions of Coral Sea SSTAs in the climate-model environment (Supplementary Figs. 17b, 18, 20b, 21, 22b and 23).

### Comparison with independent coral datasets

We use two multi-century five-year-resolution coral series from the central GBR^23,24^ (Fig. 2b and Supplementary Fig. 24) and a network of sub-annual and annual resolution modern coral series (dated from 1900 onwards but not covering the full 1900–1995 period) from 44 sites in the GBR (Supplementary Information section 4.2) for independent evaluation of coral-derived evidence for warming in the region. We estimate five-year GBR SSTAs (Fig. 2b) by aligning the post-1900 mean and variance of the proxy and instrumental (ERSSTv5) data.

### Reconstruction sensitivity to non-SST influences

Of the 22 available coral series, 16 are records of δ^18^O, a widely used measure of the ratio of the stable isotopes ^18^O and ^16^O. In the tropical Pacific Ocean, δ^18^O is significantly correlated with SST^61–64^. Coral δ^18^O is also sensitive to the δ^18^O of seawater^65^, which can reflect advection of different water masses and/or changes in freshwater input, such as from riverine sources or precipitation, which in turn co-vary with SSS. Thus, it is generally considered that the main non-SST contributions to coral δ^18^O are processes that co-vary with SSS^62,66^. Our methodology minimizes the influence of non-temperature impacts on the reconstruction by exploiting the contrast in spatial heterogeneity between SST and SSS in January–March (Supplementary Information section 5.1). SSS is spatially inhomogeneous in the tropical Pacific^66,67^, leading to low coherence in SSS signals across our coral network. By contrast, the strong and coherent SST signal across our coral network locations and the Coral Sea region leads to principal components that are strongly representative of SST variations. This produces a skilful reconstruction of SST, as determined by evaluation against independent observations, and low correlations with SSS across the Coral Sea region (Supplementary Fig. 31).

Although the likelihood of non-SST influences on our SST reconstruction is low, we nonetheless test the sensitivity of our reconstruction and its associated interpretations to the possibility of these influences on the coral data. The tests compute the correlations between our best-estimate SSTA reconstruction (highest coefficient of efficiency) and observations of SSS, along with a series of additional reconstructions based on subsets of our coral network. The correlations between our highest coefficient of efficiency January–March Coral Sea SSTA reconstruction and January–March SSS are mapped for the Coral Sea and its neighbouring domain using three instrumental SSS datasets (Supplementary Fig. 31). Correlations are not statistically significant over most of the domain. Noting differing spatial correlation patterns between the instrumental SSS datasets^68^, which also cover different time periods (Supplementary Information section 5.1), we undertake six sensitivity tests using subsets of the coral network (Supplementary Information section 5.2). We use the following combinations of coral series: (1) the full network of 22 δ^18^O and Sr/Ca series (Figs. 2a and 3); (2) a subset of the six available Sr/Ca series (Supplementary Figs. 32–33), to test how the reconstruction is influenced by the inclusion of coral δ^18^O records; (3) a fixed nest subset of the five longest coral series, extending back to at least 1700 (Supplementary Figs. 34–35), to test for the potential influence of combining series of differing lengths (from our splicing of portions of the best reconstructions from each nest); (4) a subset of the ten coral series that are most strongly correlated with the target (Supplementary Figs. 36 and 37), to test how our reconstruction is influenced by the inclusion of coral series that are less strongly correlated with our target; (5) a subset of coral series that excludes the six records that are reported to potentially include biological mediation or non-climatic effects, or have low correlation with the target (Supplementary Figs. 38 and 39), to test their influence on the reconstruction; and (6) a network perturbation test comprising 22 separate subsets of proxies, in which proxy records are added incrementally in order of highest to lowest correlation with the target, starting with a single coral series and increasing the number of included proxies to all 22 series in our network (Supplementary Information section 5.2.5), to systematically quantify the influence of gradually including more coral datasets on our reconstruction and its interpretations.

The evaluation metrics (Fig. 2c and Supplementary Figs. 32b, 34b, 36b and 38b) indicate a skilful reconstruction back to 1618 for the reconstructions based on the Full, Sr/Ca only, Long, Best-10 and OmitBioMed networks. These reconstructions explain 82.7%, 80.6%, 77.6%, 79.8% and 80.4% (R-squared values) of the variance in January–March SSTAs, respectively, in the independent evaluation periods (using ERSSTv5b). All coral subsets in the network perturbation test produce skilful reconstructions (Supplementary Fig. 40). The highest-skill reconstructions for all subsets in the network perturbation test align with our key interpretations (Supplementary Figs. 41 and 42). Together, our sensitivity tests show that the coral network, observational data and reconstruction methodology are a sound basis for reconstructing Coral Sea January–March SSTAs in past centuries and contextualizing recent high-SST events (Supplementary Information).

### Climate-model attribution ensembles and experiments

The multi-model attribution analysis used here is based on simulations from CMIP6. We analyse simulations from the historical experiment (including natural and anthropogenic influences for 1850–2014) and the historical-natural experiment (natural-only forcings for 1850–2014). We select climate models for which monthly surface temperature is available in at least three historical and historical-natural simulations (Supplementary Table 5). All model simulations are interpolated to a common regular 1.5° × 1.5° latitude–longitude grid. January–March SSTAs relative to 1961–90 are calculated for each simulation. The full historical all-forcings ensemble is composed of 14 models with 268 simulations for 1850–2014. The natural-only ensemble is composed of the same 14 models with 95 individual simulations. A subset of climate models in the CMIP6 ensemble are considered by the science community to be ‘too hot’, simulating warming in response to increased atmospheric carbon dioxide concentrations that is larger than that supported by independent evidence^31^. We omit these models from our analysis by including only models with a transient climate response in the ‘likely’ range^31^ of 1.4–2.2 °C. Our results are not strongly sensitive to this selection (Supplementary Information section 6.3). The ten remaining models yield a total of 25,410 years from 154 historical ensemble members and 7,095 years from 43 historical-natural ensemble members. We weight the models equally in our analysis using bootstrap sampling. We report linear trends based on simple linear regression models fitted with ordinary least squares. The statistical significance of linear trends is assessed using the Spearman’s rank correlation test^69^.

### Time of emergence of the anthropogenic impact

We assess the anthropogenic influence on SSTAs in the Coral Sea region by starting with the assumption that any anthropogenic influence on SSTAs in the Coral Sea is indistinguishable from natural variability at the commencement of the model experiments. We measure the impact of anthropogenic influence on the climate in the region using a signal-to-noise approach^32,70^. We calculate the anthropogenic ‘signal’ as the mean of the difference between the smoothed (using a 41-year Lowess filter) modelled historical Coral Sea SSTA and the mean smoothed modelled historical-natural SSTA. Our ‘noise’ is the standard deviation of the difference between the modelled historical SSTA and its smoothed time series (Supplementary Information section 6).

Methods additionally rely on Supplementary Information and refs. ^71–104^.

## Online content

Any methods, additional references, Nature Portfolio reporting summaries, source data, extended data, supplementary information, acknowledgements, peer review information; details of author contributions and competing interests; and statements of data and code availability are available at 10.1038/s41586-024-07672-x.

### Supplementary information

Supplementary Information .

## Data Availability

The ERSSTv5 instrumental SST data are available from the US National Oceanic and Atmospheric Administration at https://psl.noaa.gov/data/gridded/data.noaa.ersst.v5.html. The HadISST1.1 data are available from the UK Met Office at https://www.metoffice.gov.uk/hadobs/hadisst/. The original coral palaeoclimate data are available at the links provided in Supplementary Table 2. Land areas for maps are obtained from the Mapping Toolbox v.23.2 in Matlab v.2023b and the Global Self-consistent, Hierarchical, High-resolution Geography (GSHHS) Database at https://www.soest.hawaii.edu/pwessel/gshhg/ through the m_map toolbox by R. Pawlowicz, available at https://www.eoas.ubc.ca/%7Erich/map.html. Prepared data from the coral geochemical series, reconstructions and climate models that support the findings of this study are available at: 10.24433/CO.4883292.v1.

## Code availability

The code that supports the findings of this study is available and can be run at : 10.24433/CO.4883292.v1.
