# Supplementary material for: Highest ocean heat in four centuries places Great Barrier Reef in danger
Source: Nature. 2024 Aug 7;632(8024):320–6. doi: 10.1038/s41586-024-07672-x (PMC11306100; doi:10.1038/s41586-024-07672-x)
Supplement: Supplementary file 1 — Supplementary Information . [file 41586_2024_7672_MOESM1_ESM.pdf]

nature portfolio

https://doi.org/10.1038/s41586-024-07672-x

Supplementary information

Highest ocean heat in four centuries places Great Barrier Reef in danger

In the format provided by the authors and unedited

Supplementary Information for:

Highest ocean heat in four centuries places Great Barrier Reef in danger

Henley et al.

Table of Contents

1. Instrumental data 3

1.1. Location of GBR observed SST grid cells and GBR relative to Coral Sea 3

1.2. Early instrumental data and frequency of ICOADS observations 3

1.3. Correlation between detrended Coral Sea and GBR SSTs 5

2. Palaeoclimate data 6

2.1. Palaeoclimate metadata 6

2.2. Palaeoclimate data timeseries 7

2.3. Data infilling using RegEM 12

2.4. Proxy correlations with instrumental data 14

3. Reconstruction evaluation and methodology tests 15

3.1. Evaluation metrics 15

3.1.1. RE: Reduction of Error 16

3.1.2. CE: Coefficient of Efficiency 16

3.1.3. Other metrics 17

3.2. Selection of Principal Components (PC truncation) 18

3.3. Pseudoproxy reconstructions with CESM-LME 21

3.3.1. Pseudoproxy methodology and CESM-LME data 21

3.3.2. Perfect proxy experiment (testing the PCR method and spatio-temporal availability of proxy data) 23

3.3.3. Pseudoproxy experiments to assess influence of measurement error on reconstruction skill 26

4. Evaluation with independent datasets 32

4.1. Additional independent 5-year resolution record (Calvo et al. 2007) 32
4.2. Independent evaluation with modern coral records from the GBR 33
5. Reconstruction sensitivity to non-SST parameters 42
5.1. Analysis of possible confounding impacts of salinity on reconstruction 42
5.2. Sensitivity to proxy network 44
5.2.1. Sr/Ca network 44
5.2.2. Long network 46
5.2.3. Best-10 proxies 48
5.2.4. OmitBioMed 50
5.2.5. Proxy network perturbation 52
5.2.6. Comparisons across proxy subsets and instrumental datasets 57
5.2.7. Sensitivity to averaging window of sub-annual coral records 59
6. Climate model attribution with CMIP6 models 62
6.1. Model runs available and transient climate responses 62
6.2. Signal and noise for TCR-likely, equal numbers of model ensemble members...... 63
6.3. Sensitivity of time of emergence to model subset and ensemble numbers 64

1. Instrumental data

1.1. Location of GBR observed SST grid cells and GBR relative to Coral Sea

Figure S1a shows the seven grid point locations used by the Australian Bureau of Meteorology for their official Great Barrier Reef mean SST timeseries. Figure S1b shows the location of the GBR region in the broader Coral Sea region.

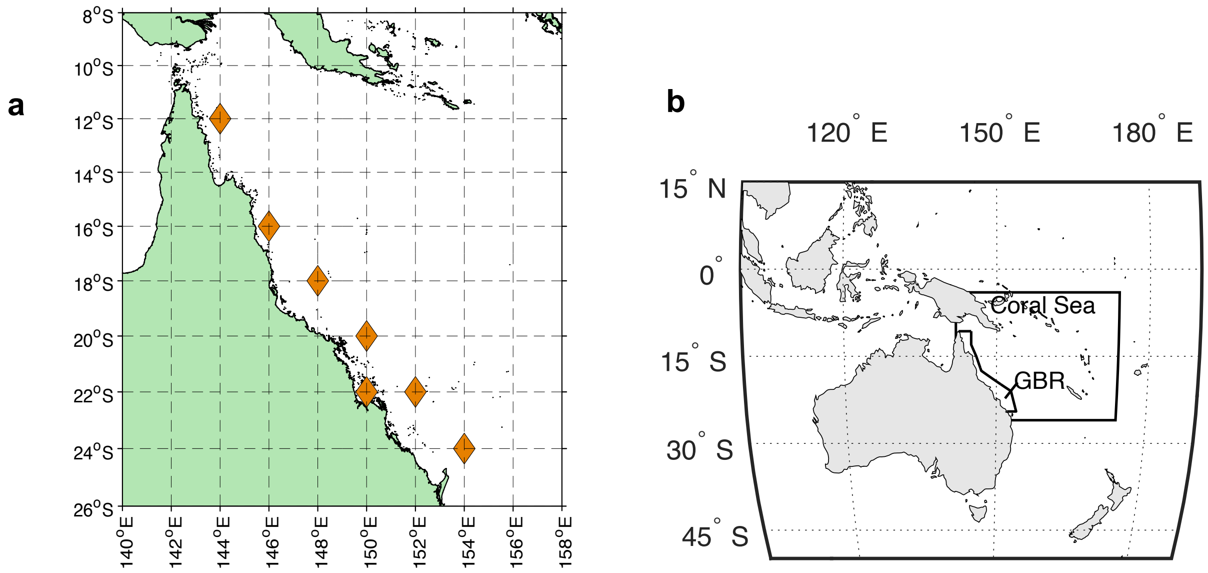

Figure S1 | Great Barrier Reef and Coral Sea locations. a, Seven Great Barrier Reef grid point SST locations used in the Australian Bureau of Meteorology's official Great Barrier Reef mean SST timeseries. **b,** Coral Sea and GBR region locations relative to Australian coast and surrounding countries. More information about the Bureau of Meteorology's GBR SST series is available at: http://www.bom.gov.au/climate/change/about/sst_timeseries.shtml)

1.2. Early instrumental data and frequency of ICOADS observations

In Figure S2 we show the percentage of months in which there are ship observations in the International Comprehensive Ocean-Atmosphere Data Set (ICOADS) dataset[71] in 20-year windows from 1860 to 1979. ICOADS is one of the datasets on which both of the gridded datasets used in this study, ERSSTv5[72] and HadISST1.1[28], are primarily based.

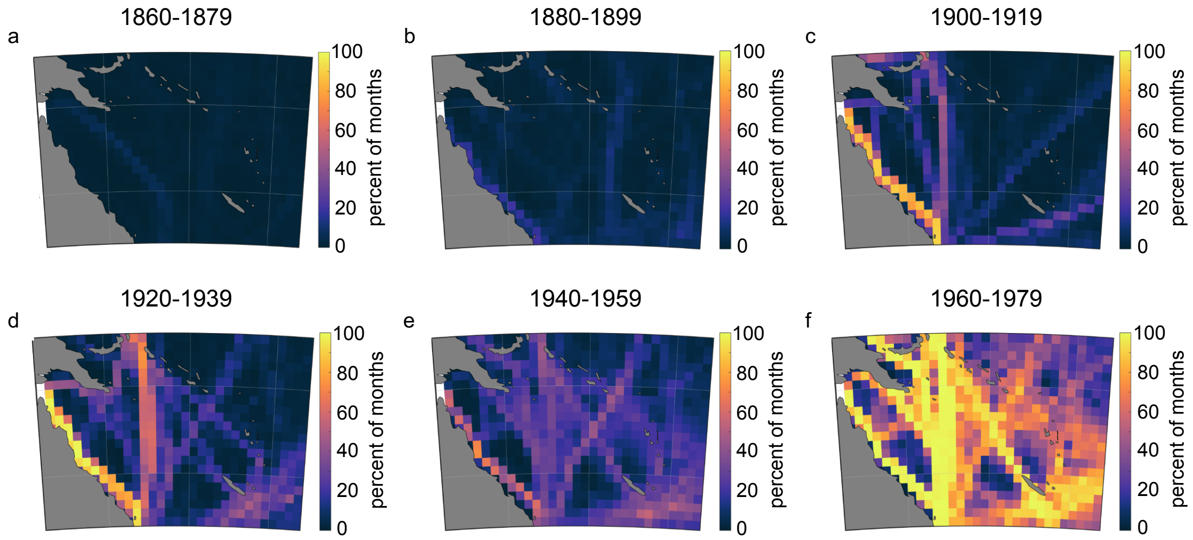

Figure S2 | Changing availability of ship observations in ICOADS over time. a-f, Percentage of months in which there are ship observations in ICOADS in 20-year periods from 1860 to 1979, as shown.

Prior to 1900 there are very few observations in the Coral Sea and the GBR, under 20% of months for most of the Coral Sea domain. We infer therefore that the uncertainties in both ERSSTv5 and HadISST1.1 are likely to be very high prior to 1900 in these two regions. In Ref [72] the total uncertainty in ERSSTv5 is the sum of parametric and reconstruction uncertainties. Inspection of Figures 1a and 3a in Ref [72] indicates that prior to 1900 the 1σ parametric uncertainty in the Coral Sea and GBR regions in ERSSTv5 is 0.4–0.8°C, and the (additional) reconstruction uncertainty is 0.4–0.6°C. When superimposed, the error totals 0.8–1.4°C. At a distance of 1.64 standard deviations away from the mean, comparable to our 90% coral reconstruction uncertainties (bounds of 5–95%), the total ERSSTv5 uncertainty is 1.3–2.3°C. With these large uncertainties prior to 1900, our coral-based reconstruction is actually potentially more accurate than these instrumental gridded datasets due to their extremely sparse input data prior to 1900. We have therefore truncated the instrumental data to include only the post-1900 period in our main analysis. This is consistent with the Australian Bureau of Meteorology's use of the ERSSTv5 data for their GBR SST timeseries, which commences in 1900 (Supp. Section 1.1). Figure S3 shows timeseries of the Coral Sea and GBR Jan–Mar SSTa in both ERSSTv5 and HadISST1.1, with the pre-1900 period dotted to indicate its high uncertainty.

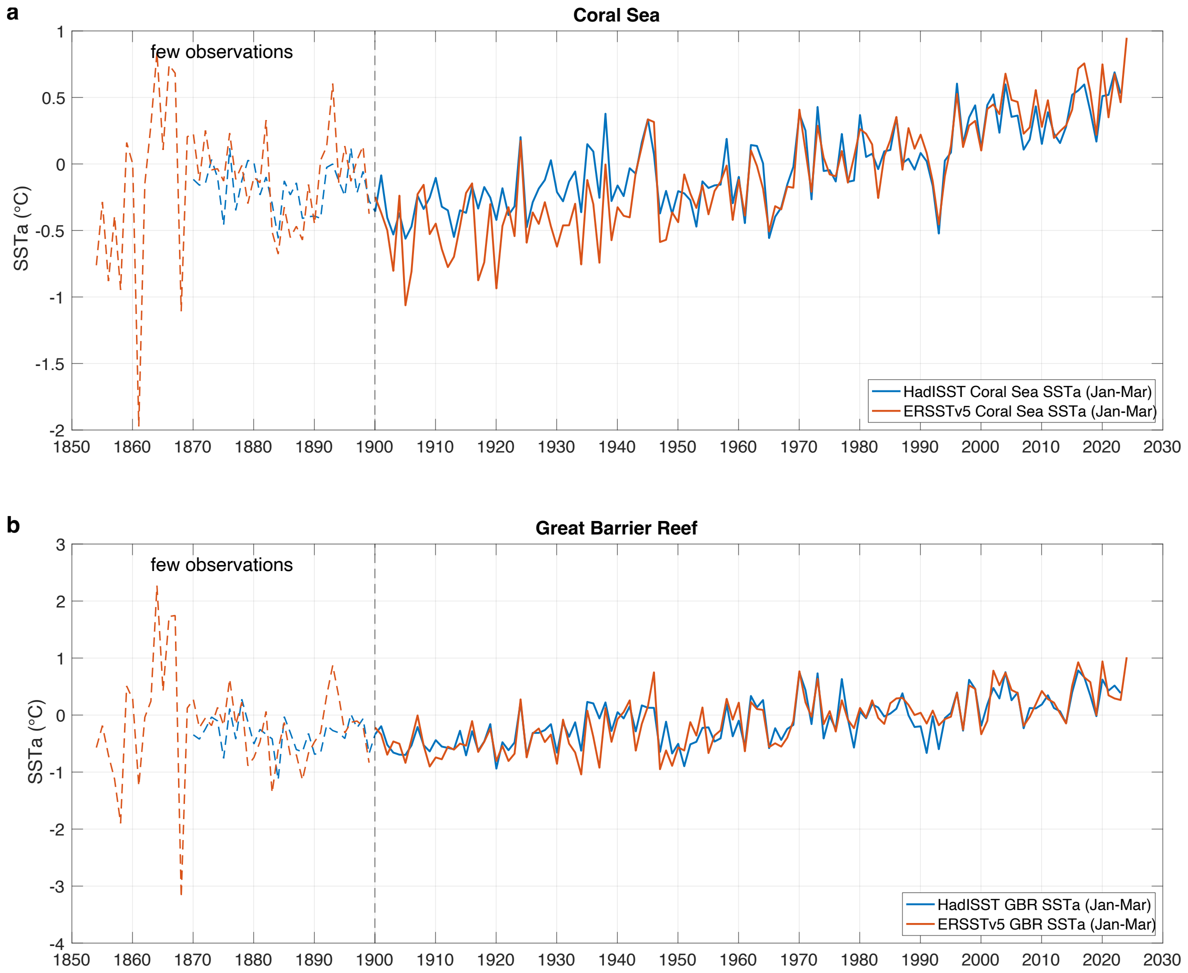

Figure S3 | Coral Sea and GBR Jan-Mar SSTa. Area average mean Coral Sea (a) and Great Barrier Reef (b) Jan–Mar SSTa for the full time periods of available data in ERSSTv5 (1854–2024) and HadISST1.1 (1870–2023) gridded instrumental datasets. Note that very few observations (under 20% of months over most of the domain, see Figure S2) are available prior to 1900 (dashed lines), so the SSTa in this period is highly uncertain.

1.3. Correlation between detrended Coral Sea and GBR SSTs

Figure S4, similar to Figure 1d, shows detrended Coral Sea and GBR region Jan–Mar SST anomalies for 1900–2024. The two timeseries remain strongly correlated ($r=0.69$, $p<0.001$) when the long-term warming signal is removed from both series. These timeseries are detrended by first differencing (subtracting each data point from the next).

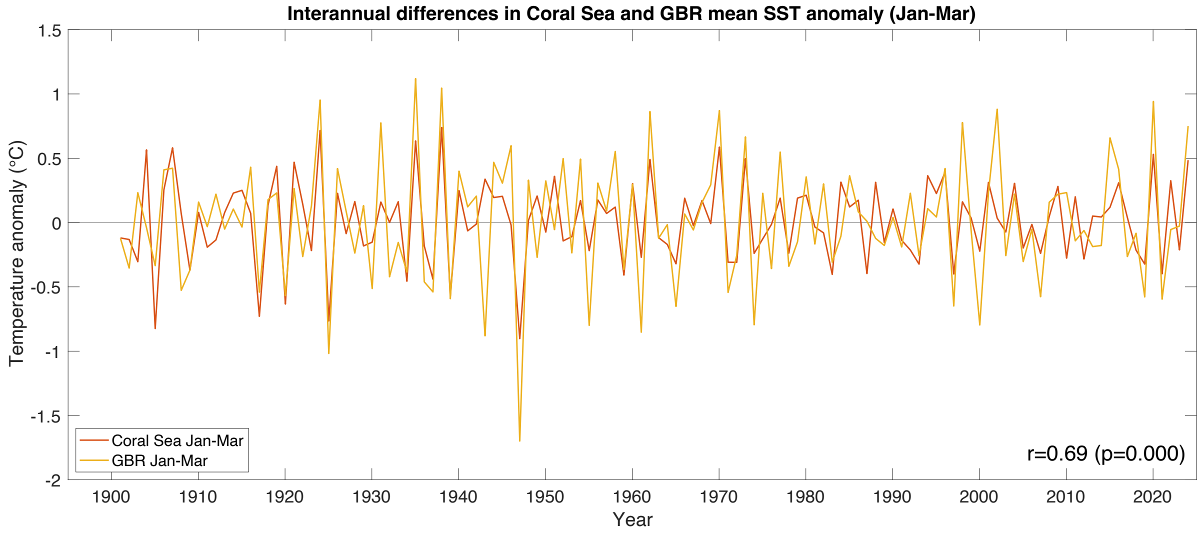

Figure S4 | Detrended Jan–Mar SST anomalies. Timeseries of detrended mean Jan–Mar SSTa from ERSSTv5 for the Coral Sea and GBR regions; detrended by first differencing (subtracting each data point from the next).

2. Palaeoclimate data

2.1. Palaeoclimate metadata

The key metadata for the coral palaeoclimate series used in our reconstructions are shown in Table S1, spanning the period 1617–2007 CE. The full reference for each record is given in the reference list. The 'networks' column refers to whether the record is included in (only) the full reconstruction, or additionally, one or more of the subsets of coral series used as sensitivity tests in this study and defined in Supp. Section 5.2. The coral data are available at the URLs shown in Table S2. Additional modern coral data with data during but not fully covering the period 1900–2017, used for independent evaluation in this study, is described and analysed in Supp. Section 4.2.

Table S1 | Palaeoclimate network metadata[73–86]

Series No.	Site name	Proxy	Country	Year from	Year to	Resolution	Reference	Reference DOI	Networks
1	Abraham Reef	$\delta^{18}O$	Australia	1638	1983	annual	Druffel & Griffin (1999)	10.1029/1999JC900212	Full, Long, OmitBioMed
2	Amédée Island	Sr/Ca	New Caledonia	1649	1999	monthly	DeLong et al. (2012)	10.1038/nclimate1583	Full, Long, Sr/Ca, Best10, OmitBioMed
3	Amédée Island	$\delta^{18}O$	New Caledonia	1658	1992	seasonal	Quinn et al. (1998)	10.1029/98PA00401	Full, Long, Best10, OmitBioMed
4	Espiritu Santo	$\delta^{18}O$	Vanuatu	1807	1979	annual	Quinn et al. (1996)	10.1029/96GL03169	Full
5	Flinders Reef	Sr/Ca	Australia	1708	1992	annual	Zinke et al. (2023)	10.1177/20530196221142963	Full, Sr/Ca, Best10,OmitBioMed
6	Flinders Reef	$\delta^{18}O$	Australia	1708	1992	annual	Zinke et al. (2023)	10.1177/20530196221142963	Full, OmitBioMed
7	Kavieng	Sr/Ca	Papua New Guinea	1823	1997	monthly	Alibert et al. (2008)	10.1029/2006JC003979	Full, Sr/Ca
8	Laing Island	$\delta^{18}O$	Papua New Guinea	1884	1993	seasonal	Tudhope et al. (2001)	10.1126/science.1057969	Full, Best10, OmitBioMed
9	Madang Lagoon	$\delta^{18}O$	Papua New Guinea	1880	1993	seasonal	Tudhope et al. (2001)	10.1126/science.1057969	Full, OmitBioMed
10	Maiana Atoll	$\delta^{18}O$	Kiribati	1840	1994	bimonthly	Urban et al. (2000)	10.1038/35039597	Full, OmitBioMed
11	Nauru	$\delta^{18}O$	Nauru	1891	1995	seasonal	Guilderson et al. (1999)	10.1029/1999PA900024	Full, OmitBioMed
12	Nauru	$\delta^{18}O$	Nauru	1897	1995	seasonal	Guilderson et al. (1999)	10.1029/1999PA900024	Full, Best10
13	Rabaul	$\delta^{18}O$	Papua New Guinea	1867	1997	monthly	Quinn et al. (2006)	10.1029/2005JC003243	Full
14	Rabaul	Sr/Ca	Papua New Guinea	1867	1997	monthly	Quinn et al. (2006)	10.1029/2005JC003243	Full, Sr/Ca
15	Sabine Bank	$\delta^{18}O$	Vanuatu	1842	2007	monthly	Gorman et al. (2012)	10.1029/2012PA002302	Full, OmitBioMed
16	Savusavu Bay	$\delta^{18}O$	Fiji	1776	2001	seasonal	Bagnato et al. (2005)	10.1029/2004GC000879	Full, OmitBioMed
17	Savusavu Bay	$\delta^{18}O$	Fiji	1617	2001	seasonal	Linsley et al. (2006)	10.1029/2005GC001115	Full, Long, Best10, OmitBioMed
18	Savusavu Bay	Sr/Ca	Fiji	1618	2000	annual	Linsley et al. (2006)	10.1029/2005GC001115	Full, Long, Sr/Ca, Best10, OmitBioMed
19	Savusavu Bay	$\delta^{18}O$	Fiji	1781	1996	seasonal	Linsley et al. (2006)	10.1029/2005GC001115	Full, Best10, OmitBioMed
20	Savusavu Bay	Sr/Ca	Fiji	1781	1997	seasonal	Linsley et al. (2006)	10.1029/2005GC001115	Full, Sr/Ca, Best10, OmitBioMed
21	Tarawa Atoll	$\delta^{18}O$	Kiribati	1894	1990	monthly	Cole et al. (1993)	10.1126/science.260.5115.1790	Full
22	Vanua Balavu	$\delta^{18}O$	Fiji	1841	2004	monthly	Dassié et al. (2014)	10.1002/2013PA002591	Full, Best10, OmitBioMed

Table S2 | Palaeoclimate source data URLs, for the coral data series in Table S1

Series No.	Data URL
1	http://www1.ncdc.noaa.gov/pub/data/paleo/coral/west_pacific/great_barrier/abraham1999iso.txt
2	https://www.ncei.noaa.gov/pub/data/paleo/coral/west_pacific/amedee2012_UpdateStephans2004-noaa.txt
3	https://www.ncei.noaa.gov/pub/data/paleo/coral/west_pacific/amedee_1998.txt
4	https://www.ncei.noaa.gov/pub/data/paleo/coral/west_pacific/quinn1996_noaa.txt
5	https://journals.sagepub.com/doi/suppl/10.1177/20530196221142963/suppl_file/sj-xlsx-1-anr-10.1177_20530196221142963.xlsx
6	https://journals.sagepub.com/doi/suppl/10.1177/20530196221142963/suppl_file/sj-xlsx-1-anr-10.1177_20530196221142963.xlsx
7	http://www1.ncdc.noaa.gov/pub/data/paleo/coral/west_pacific/papua_new_guinea/kavieng2008.txt
8	https://www.ncei.noaa.gov/pub/data/paleo/coral/west_pacific/papua_new_guinea/laing_2001.txt
9	https://www.ncei.noaa.gov/pub/data/paleo/coral/west_pacific/papua_new_guinea/tudhope2001-dt91-7_noaa.txt
10	https://www.ncei.noaa.gov/pub/data/paleo/coral/west_pacific/urban2000_noaa.txt
11	https://www.ncei.noaa.gov/pub/data/paleo/coral/west_pacific/guilderson1999-nauru1-long_noaa.txt
12	https://www.ncei.noaa.gov/pub/data/paleo/coral/west_pacific/guilderson1999-nauru2_noaa.txt
13	https://www.ncei.noaa.gov/pub/data/paleo/coral/west_pacific/papua_new_guinea/quinn2006_noaa.txt
14	https://www.ncei.noaa.gov/pub/data/paleo/coral/west_pacific/papua_new_guinea/quinn2006_noaa.txt
15	https://www.ncei.noaa.gov/pub/data/paleo/coral/west_pacific/gorman2012.txt
16	https://www.ncei.noaa.gov/pub/data/paleo/coral/west_pacific/bagnato2005_noaa.txt
17	https://www.ncei.noaa.gov/pub/data/paleo/coral/west_pacific/linsley2006/linsley2006-AB.txt
18	https://www.ncei.noaa.gov/pub/data/paleo/coral/west_pacific/linsley2006/linsley2006-AB.txt
19	https://www.ncei.noaa.gov/pub/data/paleo/coral/west_pacific/linsley2006/linsley2006-1f.txt
20	https://www.ncei.noaa.gov/pub/data/paleo/coral/west_pacific/linsley2006/linsley2006-1f.txt
21	https://www.ncei.noaa.gov/pub/data/paleo/coral/west_pacific/cole1993-mon_noaa.txt
22	https://scholarsarchive.library.albany.edu/cas_daes_geology_etd/128/

2.2. Palaeoclimate data timeseries

Timeseries of the 22 coral palaeoclimate $\delta^{18}O$ and Sr/Ca series are shown in Figure S5 a–v. Both the original data series and the either the seasonalised data (for series with sub-annual temporal resolution) or annual data (for series with annual resolution) are shown. Sub-annually resolved series are converted to an annual timestep by averaging across the November–April window, since coral timeseries are seldom dated with absolute accuracy at monthly resolution (see Methods).

Figure S5 | Palaeoclimate source data timeseries. a–v, Original data from published sources (blue) and seasonal means or annual data (red circles) used in this study; Basic metadata for the records are shown in the title for each plot. The $\delta^{18}O$ and Sr/Ca data are inversely related to SST, so the y-axes have been inverted to aid interpretation, as per standard practice, to aid readability.

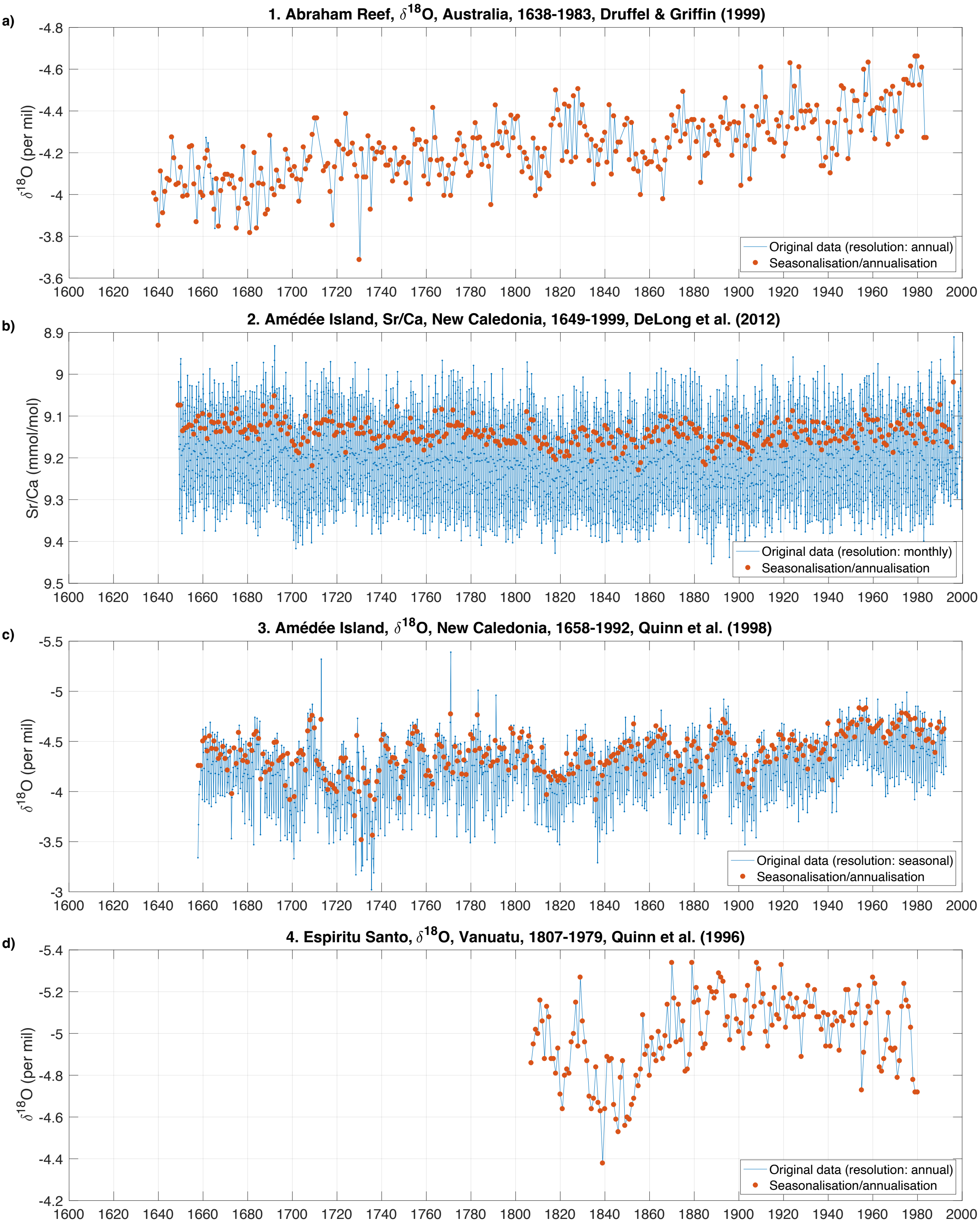

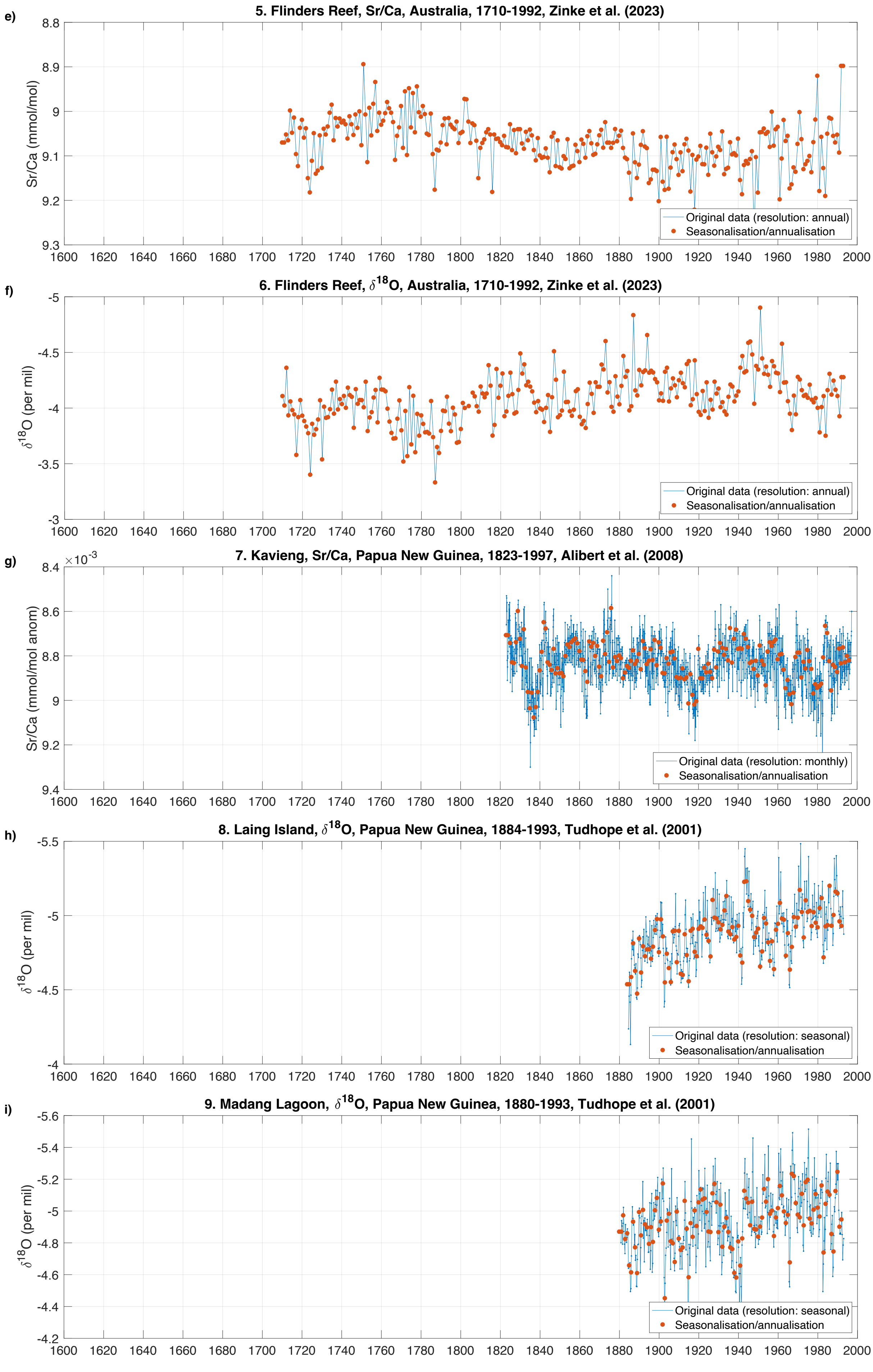
e)
5. Flinders Reef, Sr/Ca, Australia, 1710-1992, Zinke et al. (2023)
Sr/Ca (mmol/mol)
Original data (resolution: annual)
Seasonalisation/annualisation
f)
6. Flinders Reef, δ18O, Australia, 1710-1992, Zinke et al. (2023)
δ18O (per mil)
Original data (resolution: annual)
Seasonalisation/annualisation
g)
7. Kavieng, Sr/Ca, Papua New Guinea, 1823-1997, Alibert et al. (2008)
×10-3
Sr/Ca (mmol/mol anom)
Original data (resolution: monthly)
Seasonalisation/annualisation
h)
8. Laing Island, δ18O, Papua New Guinea, 1884-1993, Tudhope et al. (2001)
δ18O (per mil)
Original data (resolution: seasonal)
Seasonalisation/annualisation
i)
9. Madang Lagoon, δ18O, Papua New Guinea, 1880-1993, Tudhope et al. (2001)
δ18O (per mil)
Original data (resolution: seasonal)
Seasonalisation/annualisation

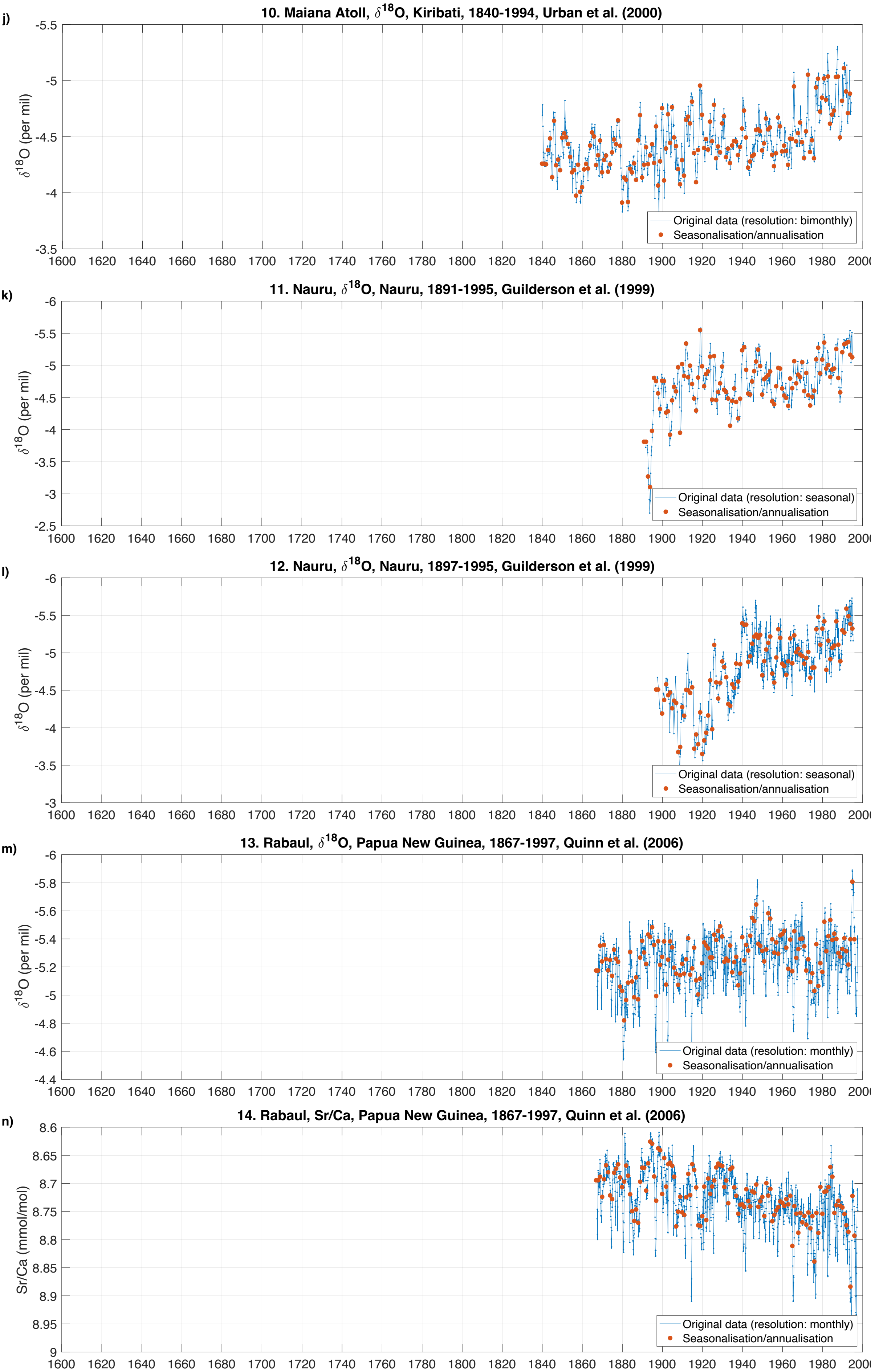
j)
10. Maiana Atoll, δ18O, Kiribati, 1840-1994, Urban et al. (2000)
δ18O (per mil)
Original data (resolution: bimonthly)
Seasonalisation/annualisation
k)
11. Nauru, δ18O, Nauru, 1891-1995, Guilderson et al. (1999)
δ18O (per mil)
Original data (resolution: seasonal)
Seasonalisation/annualisation
l)
12. Nauru, δ18O, Nauru, 1897-1995, Guilderson et al. (1999)
δ18O (per mil)
Original data (resolution: seasonal)
Seasonalisation/annualisation
m)
13. Rabaul, δ18O, Papua New Guinea, 1867-1997, Quinn et al. (2006)
δ18O (per mil)
Original data (resolution: monthly)
Seasonalisation/annualisation
n)
14. Rabaul, Sr/Ca, Papua New Guinea, 1867-1997, Quinn et al. (2006)
Sr/Ca (mmol/mol)
Original data (resolution: monthly)
Seasonalisation/annualisation
1600 1620 1640 1660 1680 1700 1720 1740 1760 1780 1800 1820 1840 1860 1880 1900 1920 1940 1960 1980 2000

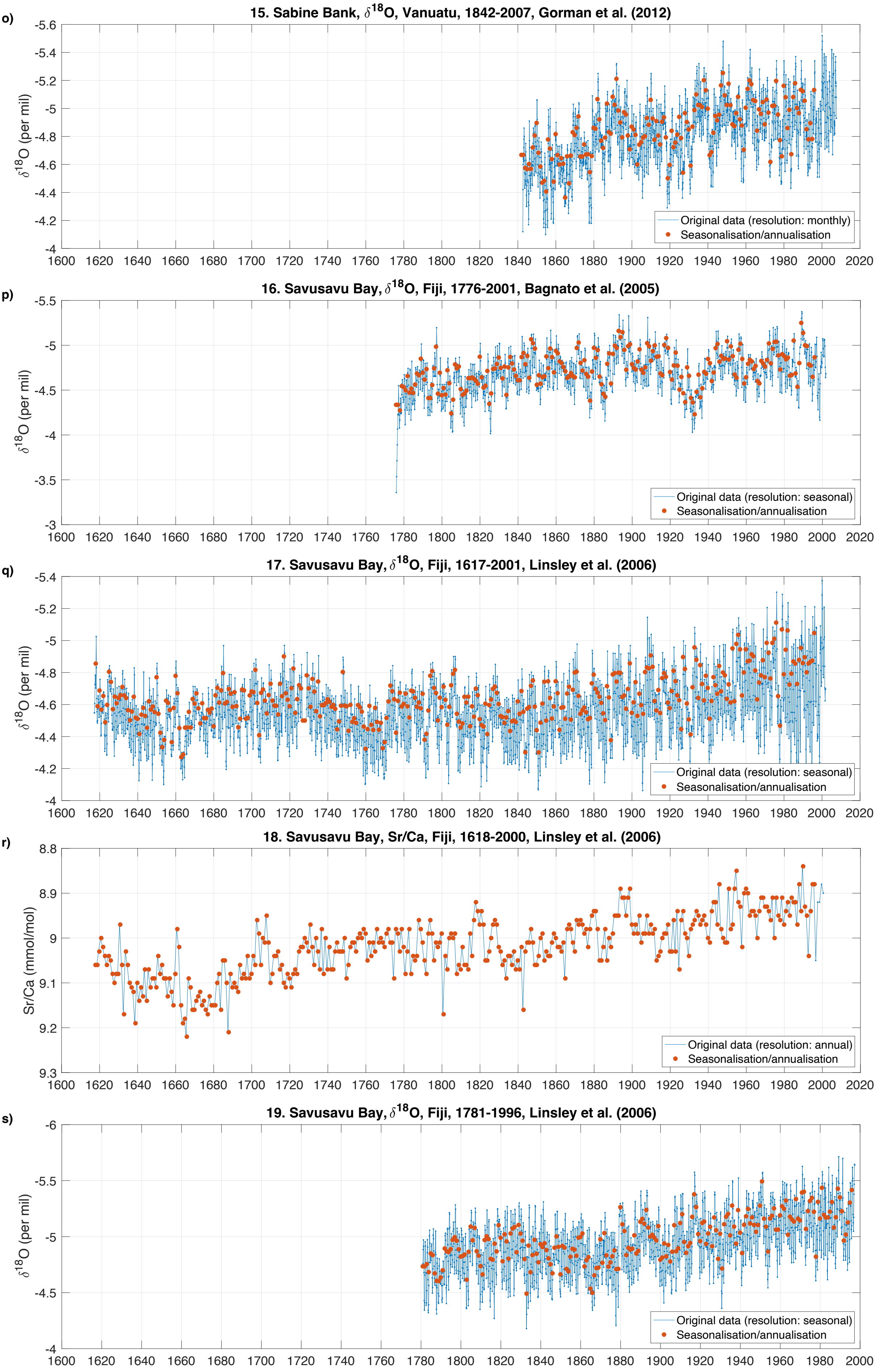
o)
15. Sabine Bank, δ18O, Vanuatu, 1842-2007, Gorman et al. (2012)
δ18O (per mil)
Original data (resolution: monthly)
Seasonalisation/annualisation
p)
16. Savusavu Bay, δ18O, Fiji, 1776-2001, Bagnato et al. (2005)
δ18O (per mil)
Original data (resolution: seasonal)
Seasonalisation/annualisation
q)
17. Savusavu Bay, δ18O, Fiji, 1617-2001, Linsley et al. (2006)
δ18O (per mil)
Original data (resolution: seasonal)
Seasonalisation/annualisation
r)
18. Savusavu Bay, Sr/Ca, Fiji, 1618-2000, Linsley et al. (2006)
Sr/Ca (mmol/mol)
Original data (resolution: annual)
Seasonalisation/annualisation
s)
19. Savusavu Bay, δ18O, Fiji, 1781-1996, Linsley et al. (2006)
δ18O (per mil)
Original data (resolution: seasonal)
Seasonalisation/annualisation

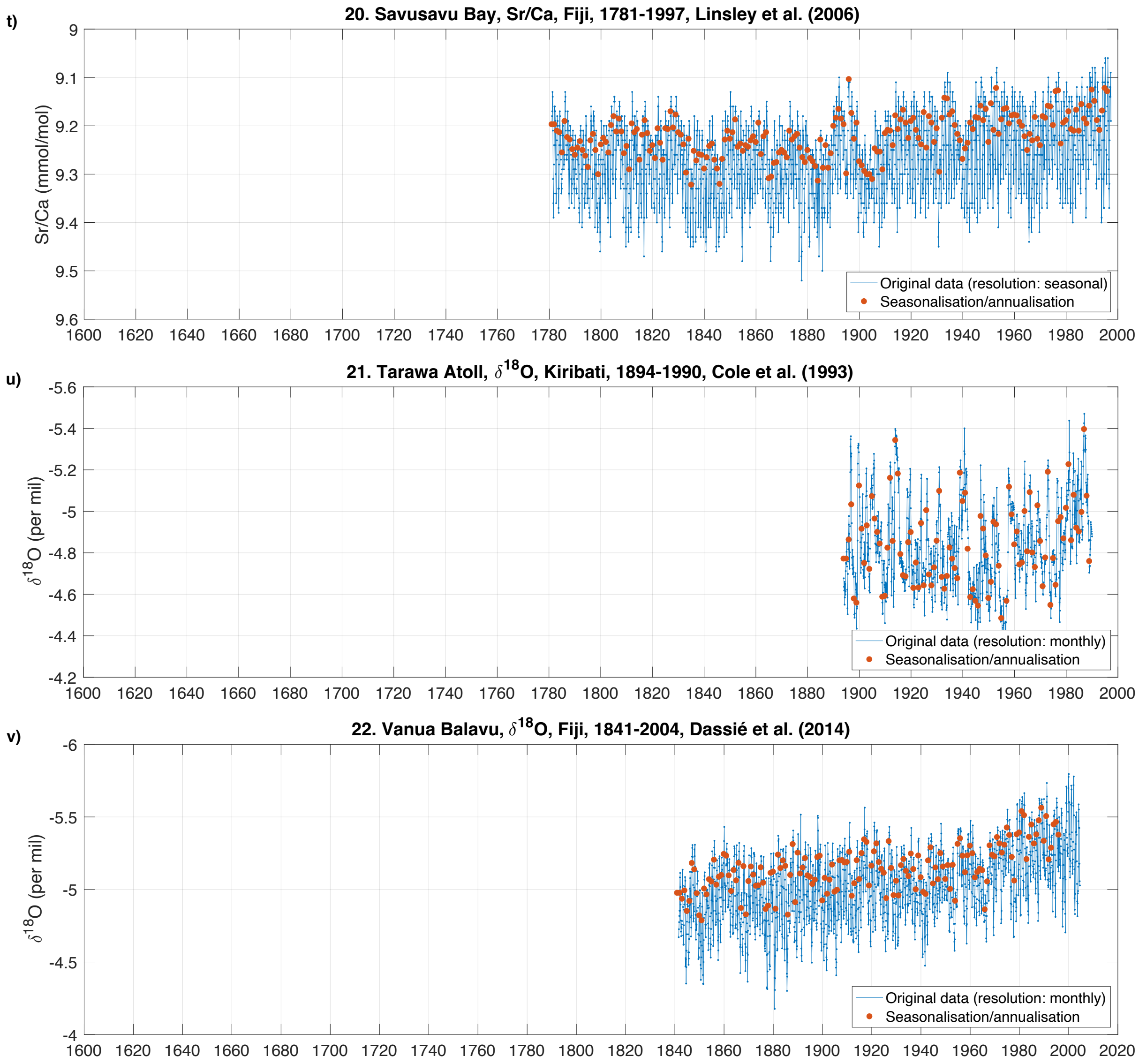

2.3. Data infilling using RegEM

There is a small proportion of missing data in the seasonalised/annualised coral series (0.8%). These data points were infilled prior to the reconstruction analysis using the Regularised Expectation Maximisation (RegEM) algorithm[56]. This ensures that the series are continuous once they have commenced, and that all series have a common ending year. This reduces the number of reconstruction nests. The data availability is illustrated below for each of the 22 coral series before (Figure S6) and after (Figure S7) infilling.

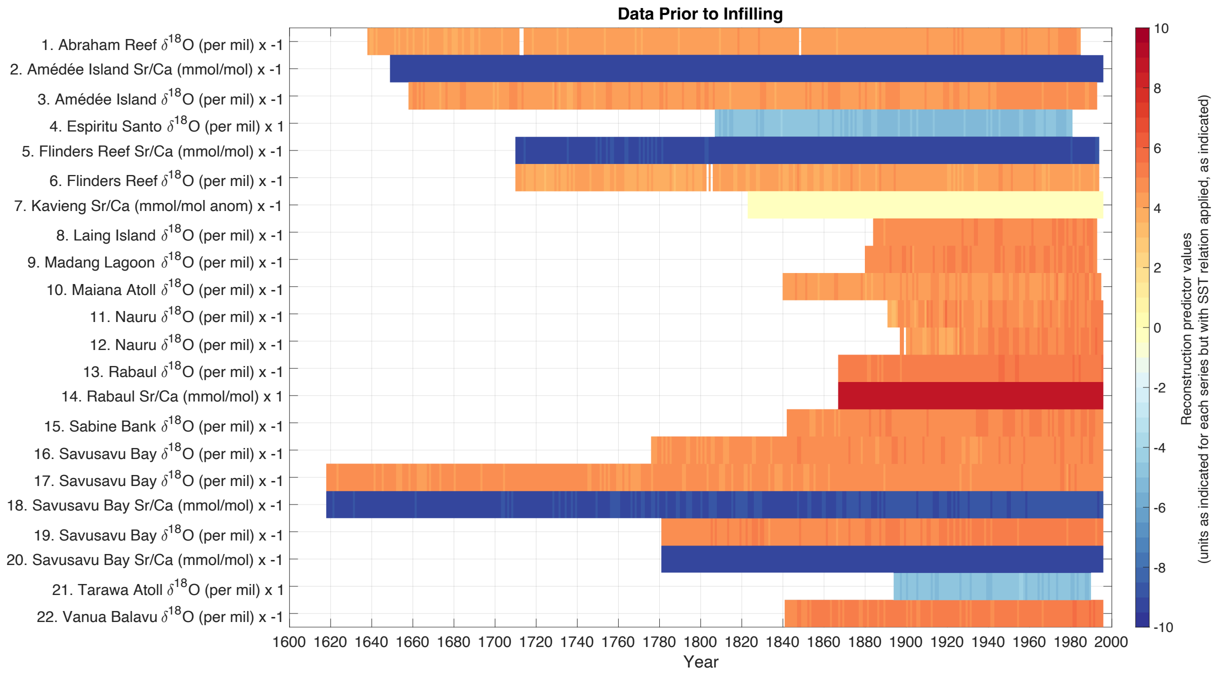

Figure S6 | Coral proxy data availability *before* infilling. Temporal availability and data values for coral geochemical series used in this study, prior to infilling with RegEM.

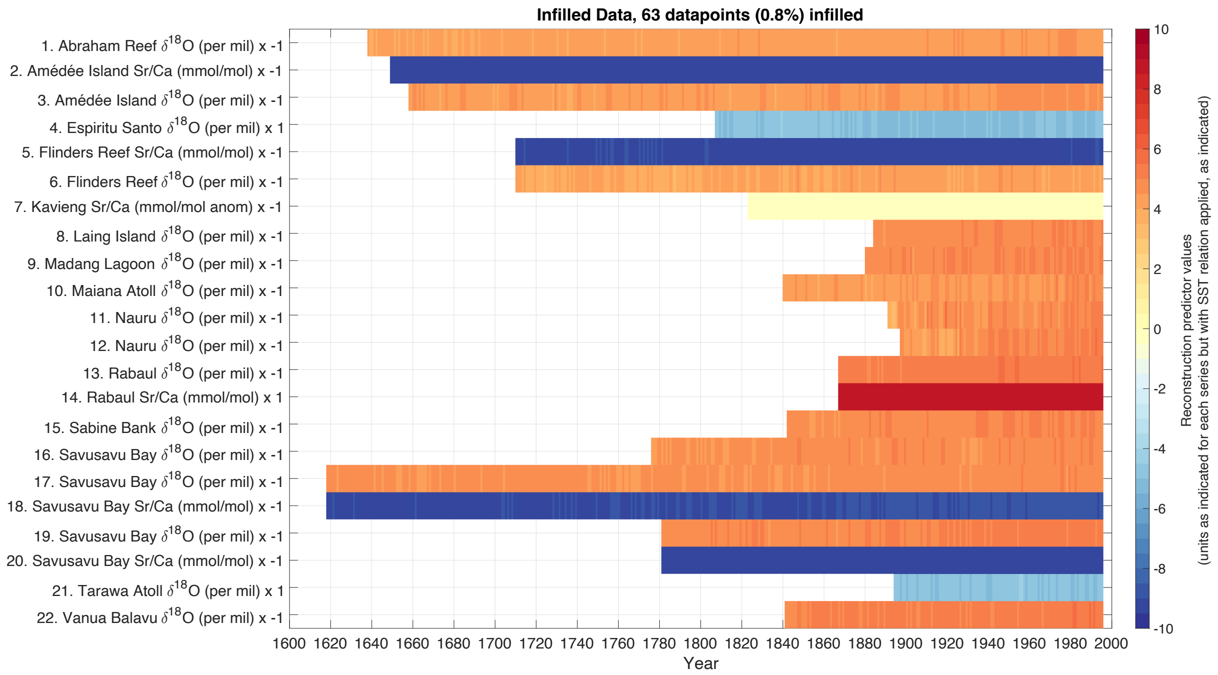

Figure S7 | Coral proxy data availability *after* infilling. Temporal availability and data values for coral geochemical series used in this study, after infilling with RegEM.

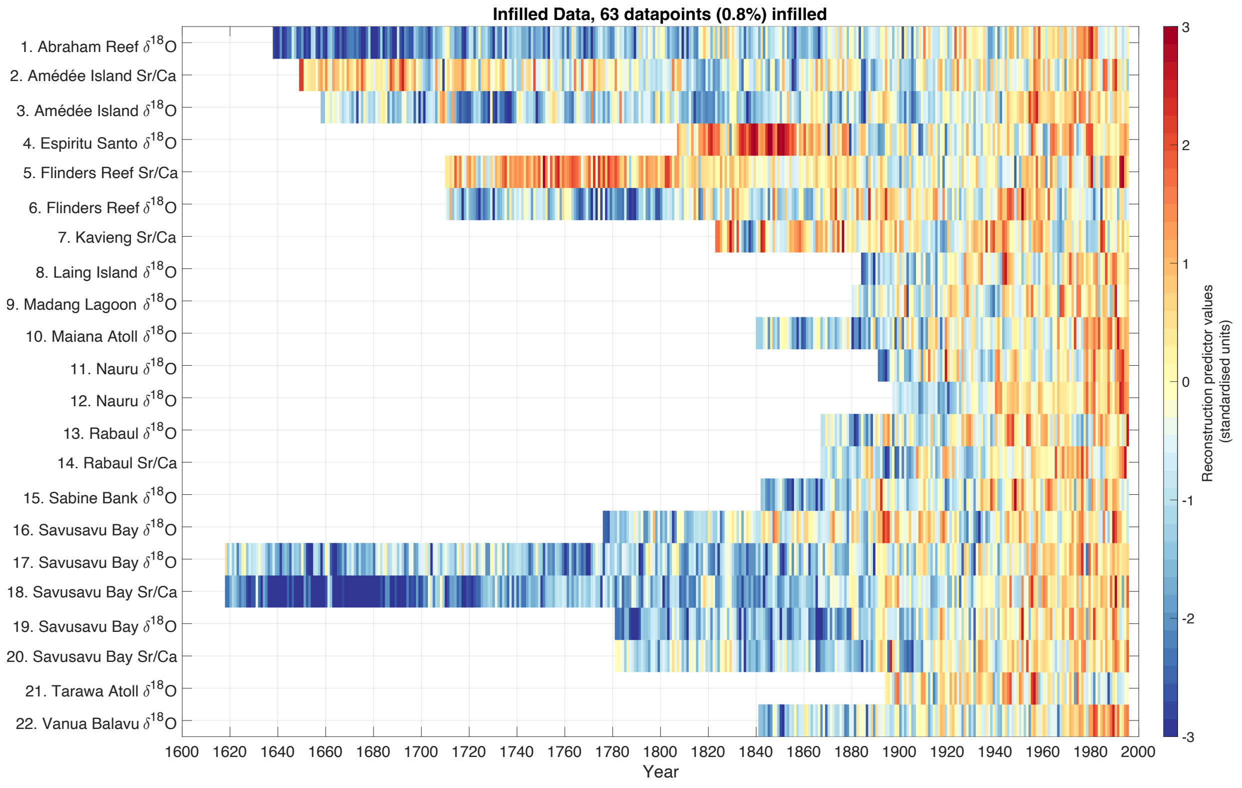

Figure S8 | Coral proxy data availability after infilling and standardisation. Temporal availability and data values for coral geochemical series used in this study, after infilling with RegEM and standardisation over the common 1900–1995 period.

2.4. Proxy correlations with instrumental data

Here we assess the correlation between the 22 coral proxy records used in this study and the ERSSTv5 and HadISST1.1 Coral Sea Jan–March average SST and the SODA 3.3.1[87], ORAS5[88], IAP[89] SSS datasets for the same region and season (Figure S9). We assess these correlations after the infilling procedure described in section 2.3. Overall, HadISST1.1 has lower correlations with the proxy data than ERSSTv5. There are substantial differences between the surface salinity correlations across the SSS datasets, including several instances of opposing sign of the correlation. This is likely due to the SSS datasets' varying temporal coverage, varying methodologies and limitations in their input data quantity and quality[68].

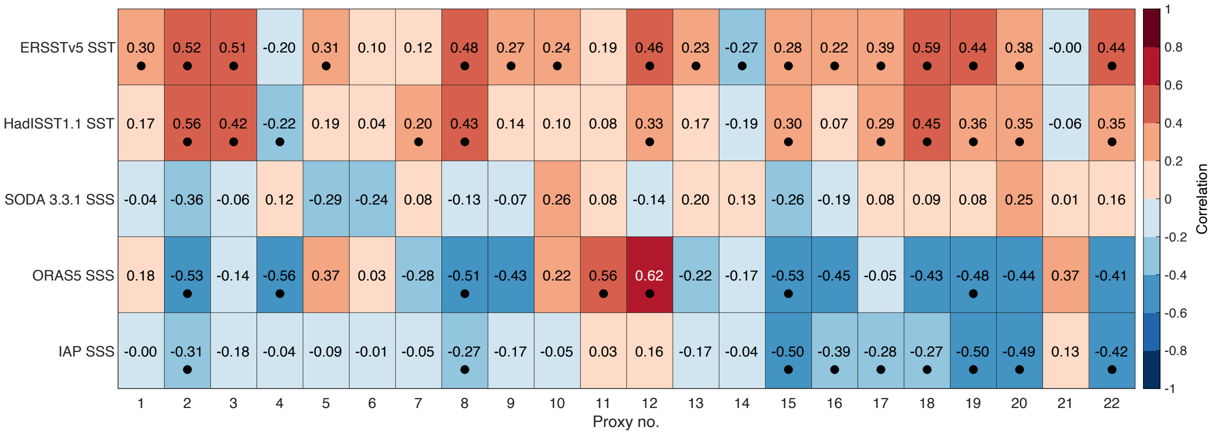

Figure S9 | Correlation between coral proxy data and instrumental regional mean SST and SSS. Correlation between, and statistical significance of, coral proxy data (x-axis) and Coral Sea regional mean SST and SSS (y-axis) for their maximum individual overlapping periods; Black dots indicate statistical significance at the 5% significance level. Proxy numbers correspond to the listing in Table S1.

3. Reconstruction evaluation and methodology tests

3.1. Evaluation metrics

We use the following 14 evaluation metrics to evaluate the skill of the reconstructions, where the term "skill" is used here to describe the overall ability of our method to accurately and precisely reconstruct the observed data, as measured collectively and/or individually by several evaluation metrics. The terms "verification" period and "evaluation" period are interchangeable here and refer to the period in which the corresponding observational and reconstruction datapoints are available for evaluation, independent of the calibration period datapoint pairs. Note that in our reconstruction approach, we vary the calibration interval durations from 0.6–0.8 of the overlapping period, and the locations vary across the full overlapping period (see Methods). Equations are provided for RE and CE since these metrics tend to be primarily used by the palaeoclimate community. Other statistical measures take their standard form.

3.1.1. *RE: Reduction of Error*

The reduction of error (RE) varies from a perfect (upper) value of 1.0 to a lower value bounded at –∞. A value of zero (or below) indicates skill that is no better (or worse) than if the reconstruction was equal to the observational mean value.

$$RE = 1 - \frac{\mathrm{SSE}_v}{V_{vc}}$$

where the sum of squared errors in the verification period ($\mathrm{SSE_v}$) is:

$$SSE_v = \sum_{i=1}^{n} (y_{recon,v,i} - y_{obs,v,i})^2$$

Where $y_{recon,v,i}$ is ith reconstruction data point during the n datapoints of the verification period and, similarly, $y_{obs,v,i}$ is for the observed data during the verification period. The observed verification period variance with respect to the calibration period mean (V_{vc}) is:

$$V_{vc} = \sum_{i=1}^{n} (y_{obs,v} - \overline{y_{obs,c}})^2$$

3.1.2. *CE: Coefficient of Efficiency*

The CE is similar to the RE, except that the verification/evaluation period mean is used in the denominator of the second term rather than the calibration period mean. Similarly to RE, the CE varies from a perfect score of 1.0, to a lower bound of –∞, and a value of zero indicates skill that is no better than if the reconstruction was equal to the observational mean value:

$$CE = 1 - \frac{\mathrm{SSE}_v}{V_{vv}}$$

where the observed verification period variance with respect to the *verification* period mean (V_{vc}) is:

$$V_{vv} = \sum_{i=1}^{n} (y_{obs,v} - \overline{y_{obs,v}})^2$$

3.1.3. Other metrics

r^2_v – the coefficient of determination in the evaluation (verification) period indicates the proportion of variance in the observations explained by the reconstruction in the evaluation period, varying from -∞ to 1.0.

r^2_c – the coefficient of determination in the calibration period is similar to r^2_v but is for the calibration period.

$RMSE_v$ – the root mean squared error in the evaluation (verification) period is a measure of the expected error in the reconstruction in the units of the target, during the evaluation period. It is the standard deviation of the reconstruction error.

$RMSE_c$ – Root mean squared error in the calibration period, similarly to $RMSE_v$ but for the calibration period.

$Corr_v$ – the correlation between reconstruction and the observations in the evaluation (verification) period indicates how well the observations and reconstruction vary together during the evaluation period, between –1.0 and 1.0.

$Corr_c$ – correlation between reconstructions and observations in the calibration period, similar to $Corr_v$ but for the calibration period.

MAE_v – the mean absolute error in the evaluation (verification) period is an additional measure of the expected error in the units of the target, during the evalutaion period.

MAE_c – the mean absolute error in the calibration period is similar to the MAE_v but for the calibration period.

$PBIAS_v$ – the proportion of bias in the mean in the evaluation (verification) period, sometimes reported as a percentage, indicates the average tendency of the reconstructed data to be larger or smaller than the observations during the evaluation period, varying from –∞ to ∞, with a perfect reconstruction obtaining a value of zero.

$PBIAS_c$ – the proportion of bias in the mean in the calibration period is similar to the $PBIAS_v$ but for the calibration period.

RE_{det} – the reduction of error of the detrended timeseries is similar to the RE, but both the observed and reconstructed data are detrended prior to the calculation. In this study we detrend the data using a fitted quadratic polynomial.

CE_{det} – the coefficient of efficiency of the detrended timeseries is similar to RE_{det} but for CE rather than RE.

3.2. Selection of Principal Components (PC truncation)

Our reconstruction methodology incorporates the first *n* principal components (PCs) of the proxy matrix in the multiple regression, where *n* is determined as the smallest number of PCs for which their total explained variance is at least *e (*%) of the overall variance. Including more PCs increases the risk of the inclusion of noise, which may lead to overfitting. Including fewer PCs may reduce the skill of the reconstruction, leading to a suboptimal reconstruction. Here we undertake two tests to optimise the truncation of PCs by varying the percentage of the sum of the variance explained by the principal components, *e,* used as predictors in the reconstruction. Our tests are:

(i) **a synthetic noise test:** where the infilled proxy matrix (see section 2.3) is replaced by lag-one autocorrelated AR(1), "red" noise with the same statistical properties (mean, variance and lag-one autocorrelation) and temporal coverage as the infilled proxy matrix, for *e* = 50, 60, 70, 80 90, 95, 97.5 and 99%; and

(ii) **a coral data test:** where the real coral data is used as per the reconstruction in the main text of this study, but with varying *e*, with *e* = 50, 60, 70, 80 90, 95, 97.5 and 99%.

For both tests we use the identical reconstruction methodology, with the ERSSTv5b SST as the reconstruction target, varying only the minimum explained variance in the PCs. We explore the effect on the CE and the RE (see section 3.1) for the range of *e*.

Figure S10 shows boxplots for the CE and RE, where the central line of the boxplot is the median value and the upper and lower edges of the box are the 25th and 75th percentile values. When the reconstruction's input data is purely noise, our method is only very rarely

capable of achieving positive CE (no part of the boxes extends above zero). For RE, the skill is centred on zero for *e*< 80% and well under zero for *e*>80%. The reconstructions based on noise achieve extremely low, or zero skill. This shows that the CE and RE are useful evaluation metrics in determining the skill of our reconstruction, because they are able objectively distinguish between reconstructions built on real data or noise. In addition, as the explained variance of the included PCs increases from 50 to 99%, the median and range of the CE and RE distribution declines, showing that as additional (possibly noisy) PCs are included, the reconstruction skill, as measured by CE and RE, declines. This demonstrates that our method does not achieve spurious skill by increasing the number of lower order (noise) PCs included in the reconstruction.

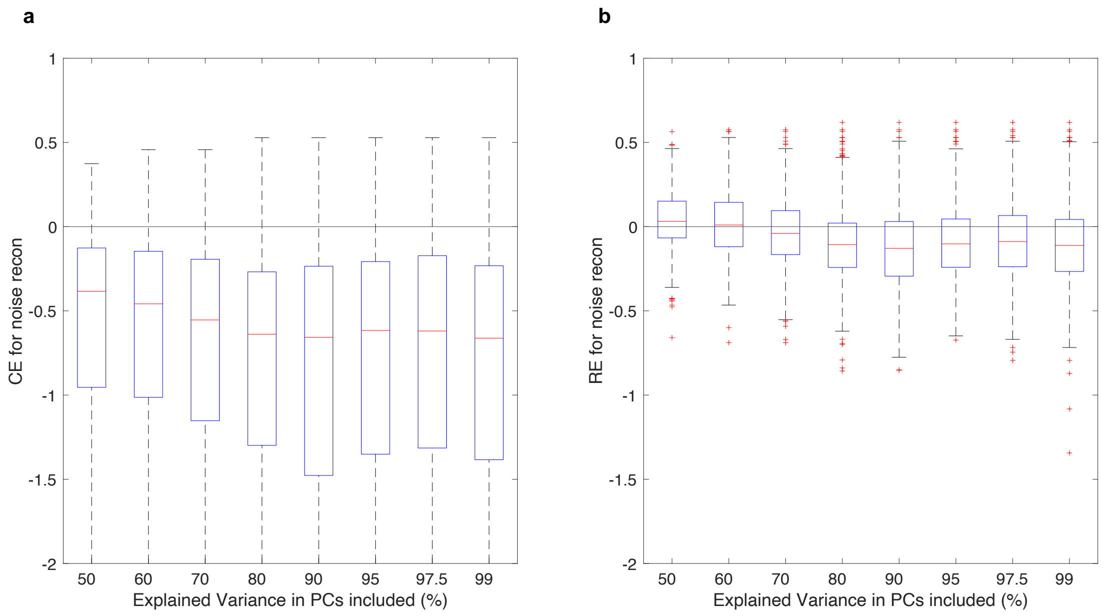

Figure S10 | Synthetic noise test skill metrics with varying explained variance in the PCs. Probability distributions of the **a,** CE and **b,** RE for reconstructions from an AR(1) noise matrix with the same statistical properties (mean, variance and lag-one autocorrelation) and temporal coverage as the proxies (the full coral network). The boxplots show the distribution of CE and RE across all reconstruction nests and calibration intervals; the sum of the explained variances in the PCs included as predictors in the reconstruction vary from 50 to 99%, as shown. In each box plot, the red central horizontal line indicates the median; the bottom and top edges of the box indicate the 25th and 75th percentiles, respectively; the whiskers extend to the most extreme data points not considered outliers; the outliers are shown as red plus signs, and an outlier is a value that is more than 1.5 times the interquartile range away from the bottom or top of the box.

Figure S11 shows the influence of a range of *e* with the real coral data. There are small but appreciable increases in skill as the PC explained variance increases from 80–95%, particularly in the lower quartile bound in the boxplots, however beyond 95% there is little change in skill. We therefore conclude that including the PCs which explain 95% of the total variance is an appropriate choice to maximise skill but maintain confidence that we are not achieving spurious skill and overfitting through the inclusion of noise. We therefore present the results for *e* = 95% in Figure 2 of the main text of this study.

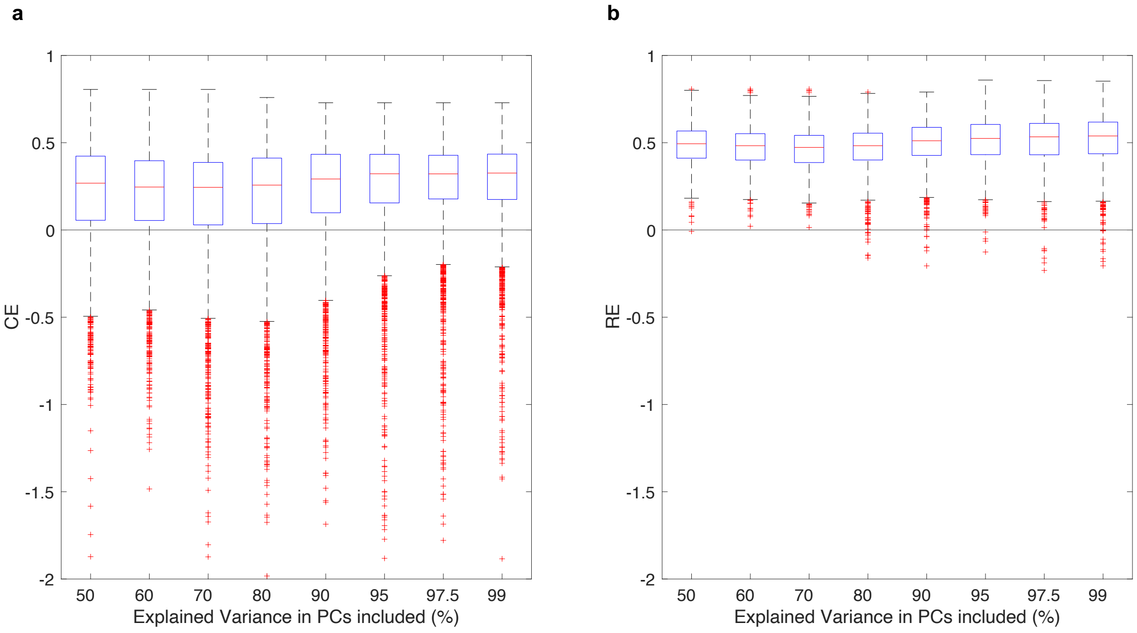

Figure S11 | Coral data test skill metrics with varying explained variance in the PCs. Probability distributions of the **a,** CE and **b,** RE for reconstructions using the coral data (full network). Similarly to Figure S10, the boxplots show the distribution of CE and RE across all reconstruction nests and calibration intervals; the sum of the explained variances in the PCs included as predictors in the reconstruction vary from 50 to 99%, as shown. In each box plot, the red central horizontal line indicates the median; the bottom and top edges of the box indicate the 25th and 75th percentiles, respectively; the whiskers extend to the most extreme data points not considered outliers; the outliers are shown as red plus signs, and an outlier is a value that is more than 1.5 times the interquartile range away from the bottom or top of the box.

3.3. Pseudoproxy reconstructions with CESM-LME

3.3.1. Pseudoproxy methodology and CESM-LME data

Here we test how representative our proxy network is of Jan-Mar SSTs in the Coral Sea, and the effectiveness of the reconstruction method at extracting Jan-Mar SSTs, using input data from the Community Earth System Model Last Millennium Ensemble[60]. The advantage of this approach is that the full target timeseries is known, and the coral-based reconstruction can be replicated using spatio-temporally matched data from within the “model world”, using the same reconstruction approach. By comparing the model’s preindustrial timeseries with the model-based reconstruction it is possible to further evaluate both the PCR method and how representative the coral network is of the regional Jan–Mar SST variability.

We use SST and SSS data from all 13 ensemble members in the CESM Last Millennium Ensemble (LME) as input data to our pseudoproxy reconstructions and compare the results across all ensemble members. A snapshot of the LME data used here is shown in the following figures for indicative purposes. Figure S12 and Figure S13 show the mean SST and SSS in the CESM-LME for the period 1850–1900 for a sample ensemble member, relative to the coral data site locations (yellow dots). Figure S14 and Figure S15 show annual and 31-year smoothed timeseries of the Coral Sea mean Jan–Mar SST and SSS, respectively, in the CESM-LME for the period 850–2005 CE for a sample ensemble member. The interannual and decadal-scale variability in SST and SSS over the centuries is evident in the model data. Both warming and freshening are observed in the post-industrial period. We deem that the LME therefore provides suitable input data for testing our reconstruction.

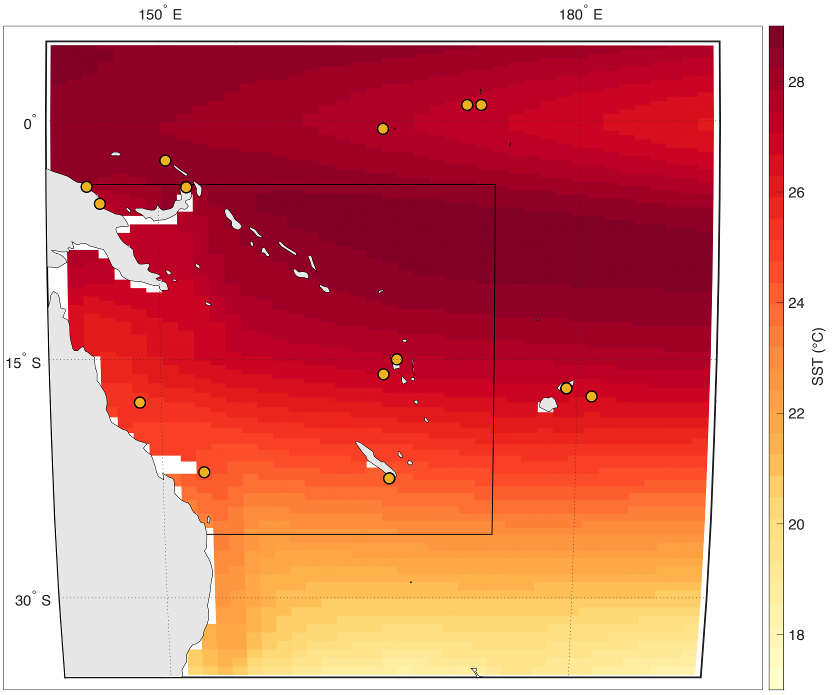

Figure S12 | Climate model mean SST relative to coral data locations. CESM-LME mean SST (°C) for 1850–1900 and coral data locations in LME ensemble member 1.

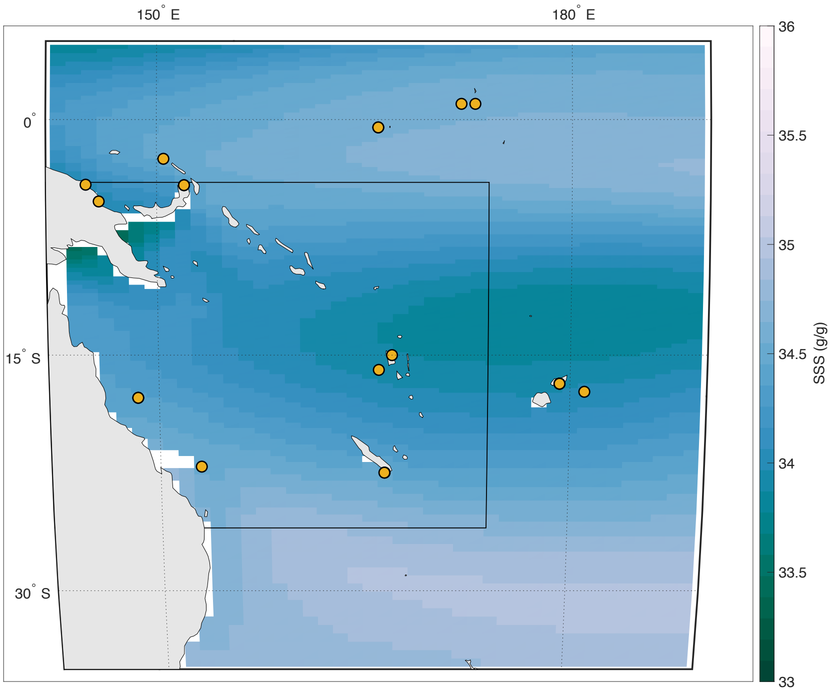

Figure S13 | Climate model mean SSS relative to coral data locations. CESM-LME mean SSS for 1850–1900 and coral data locations in LME ensemble member 1.

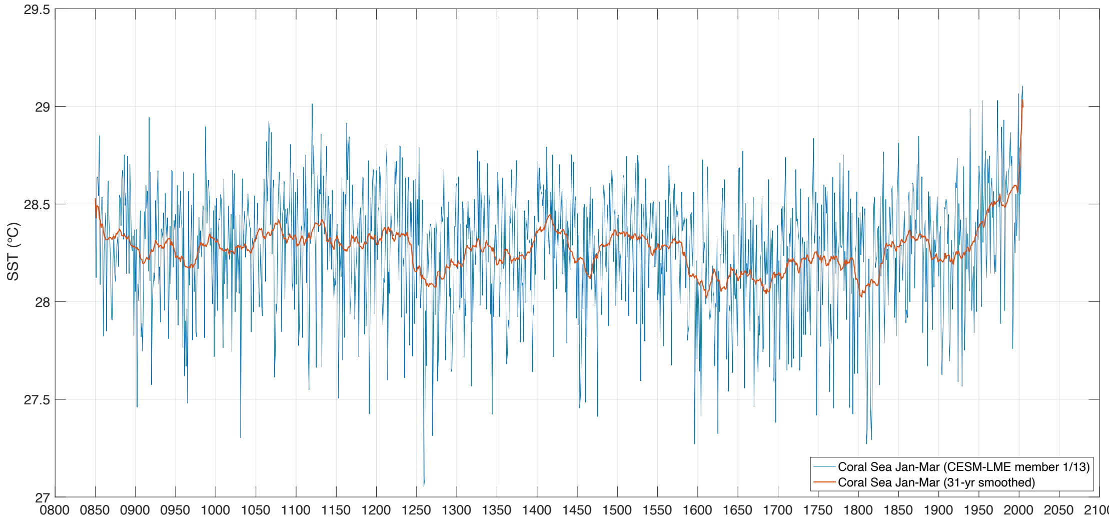

Figure S14 | CESM-LME Coral Sea mean SST for Jan–Mar for ensemble member 1.

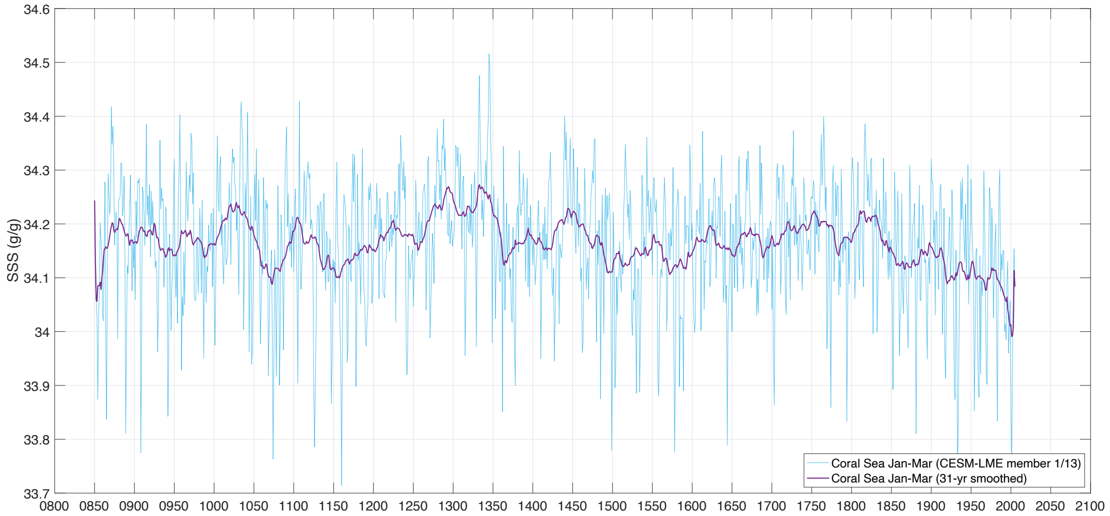

Figure S15 | CESM-LME Coral Sea mean SSS for Jan–Mar for ensemble member 1

3.3.2. Perfect proxy experiment (testing the PCR method and spatio-temporal availability of proxy data)

The CESM-LME data are used here for a 'perfect proxy' experiment, to determine whether or not the coral network's spatio-temporal availability is sufficient for our PCR method to produce a skilful reconstruction of Jan–Mar Coral Sea SSTa. SSTa data from the model, at the same locations (nearest grid point) and temporal availability as the coral network, are used directly as a substitute for the proxy data input in the reconstruction. The data are 'perfect'

proxies because they are assumed to have no non-SST influences. The perfect proxy network forms the predictor matrix at an annual timestep. The nesting and infilling procedures (Figure S16), as well as the Nov–Apr averaging window are the same as the method used for the coral reconstruction. In the perfect proxy experiment we do not apply forward (proxy system) modelling, nor is proxy measurement error included because we want to isolate the influence of the PCR method and spatio-temporal distribution of the network on reconstructing Jan-Mar SST prior to assessing the influence of these affects.

Figure S17 and Figure S18 show the results of the perfect proxy experiment. Figure S17 presents an evaluation that mirrors the evaluation undertaken in the main text, with corresponding temporal coverage of the reconstruction and pseudo-observed SST. Figure S18 shows boxplots which indicate the distribution of a range of evaluation metrics computed for the full past millennium period. The evaluation statistics indicate that the coral network and our PCR approach are suitable for obtaining skilful reconstructions of Jan–Mar Coral Sea SSTa. The timeseries of RE and CE in Figure S17 are all above zero, and the ranges of the evaluation metrics in Figure S18 indicate skilful reconstructions with, for example, low bias, and moderately high RE and CE, explained variance and correlation with the target across the past millennium.

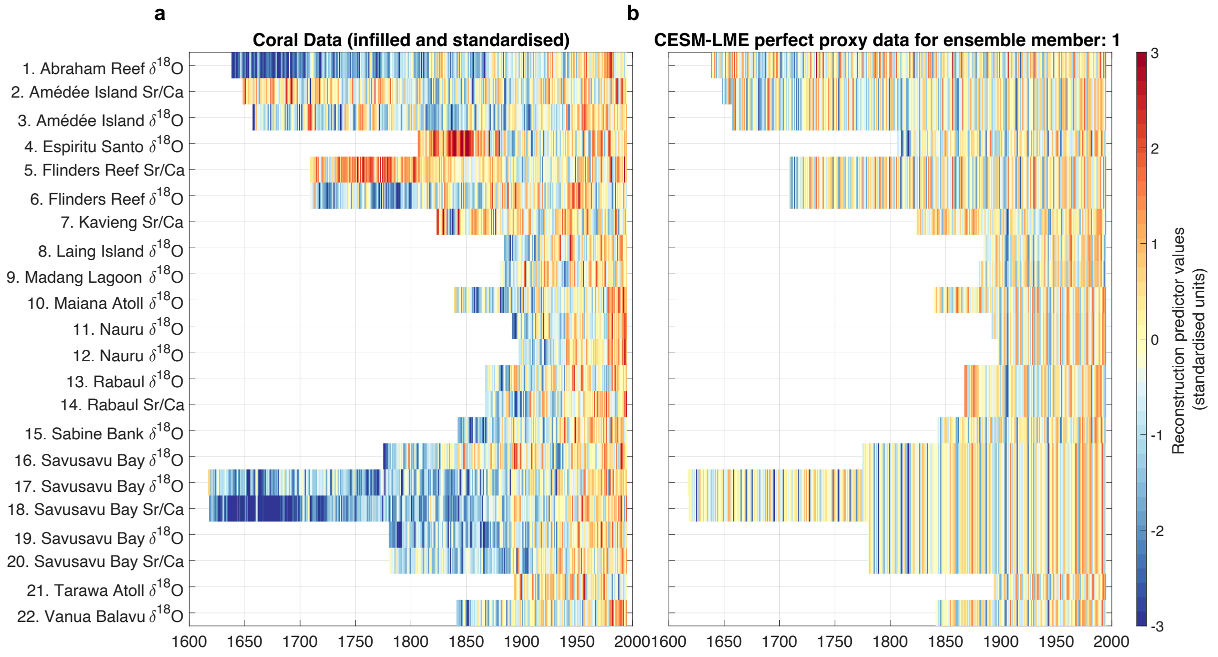

Figure S16 | Alignment of coral network and perfect proxy data availability. Data temporal availability comparison for **a,** the coral network and **b,** the pseudoproxy coral network for LME ensemble member 1 (right panel); data is in standardised units for all sites.

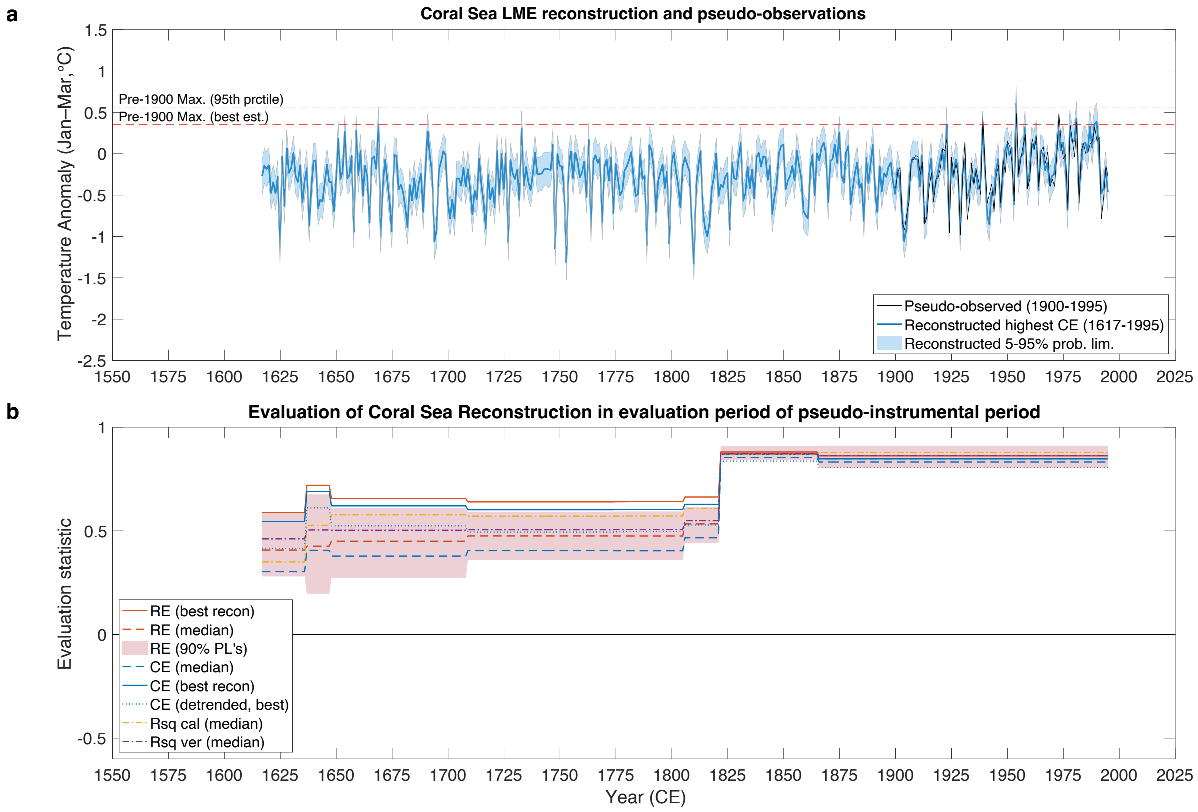

Figure S17 | Perfect Proxy experiment results for CESM-LME ensemble member 1. a, Timeseries of reconstructed (highest CE reconstruction in dark blue and 5–95th percentile probability limits in light blue shading) and pseudo-observed (black) Jan-Mar SSTa for the Coral Sea region in CESM-LME; **b,** Corresponding evaluation statistics. Note that to align with the coral reconstruction, the temporal coverage of the pseudo-observed data in this evaluation commences with the real observations (1900) and finishes in the LME simulation ending year (2005).

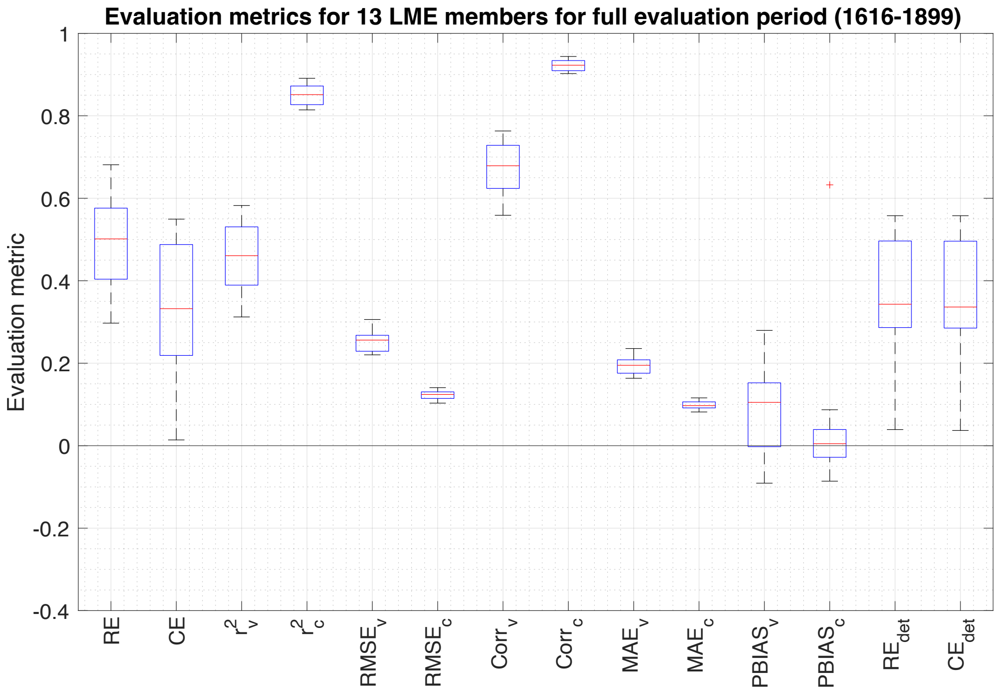

Figure S18 | Evaluation metrics for perfect proxy experiment. Statistics are shown for all CESM-LME ensemble members, evaluted over the full pre-observational period (1618–1899). Evalaution metrics are described in section 3.1. Note that the 282 year evaluation period here (1618–1899) is significantly longer than the calibration periods, which are between 0.6 and 0.8 in the overlapping period, around 60–80 years (e.g. 1930–1995, see Methods). In each box plot, the red central horizontal line indicates the median; the bottom and top edges of the box indicate the 25th and 75th percentiles; the whiskers extend to the most extreme data points not considered outliers (shown as red crosses, values that are more than 1.5 times the interquartile range away from the bottom or top of the box).

3.3.3. Pseudoproxy experiments to assess influence of measurement error on reconstruction skill

The purpose of this pseudoproxy experiment is to determine how the skill of the reconstruction varies for two levels of proxy measurement error (signal-to-noise ratio (SNR) of 4.0 and 1.0). The experiment assesses the impact of $\delta^{18}O_{seawater}$–SSS and other non-SST ‘noise’ on reconstructing SST. Our lower SNR, in particular, is likely to be an overestimate of the noise in coral Sr/Ca and $\delta^{18}O_{coral}$ records, noting the correlations presented in Figure S9.

The pseudoproxy experiments use SST and SSS data from CESM to model coral proxy data using linear regression. The Sr/Ca series are modelled as a function of SST and $\delta^{18}O_{coral}$ is modelled as a function of both SST and SSS from the model. We follow Refs [66,67,90] and use the proxy system model shown in Eq. 1 and 2 to form the pseudoproxy predictor matrix. In Eq. 1, the mean coral Sr/Ca for the reconstruction window is related to the climate model's SST through the coefficient c_1, which is obtained by linearly regressing the coral Sr/Ca (mmol/mol) against ERSSTv5 (°C). The error term is assumed to be gaussian, $\varepsilon \sim N(0,\sigma^2_\varepsilon)$, where σ^2_ε is computed for each record based on two experimental cases in which the error variance is assumed to be equal to a fixed proportion of the data variance for each coral pseudoproxy record, via a hypothesised signal-to-noise (SNR) ratio. We consider two cases of SNR, with values of 4.0 and 1.0, which we judge to represent approximate bookends for plausible high and low SNR situations.

In Eq. 2, the $\delta^{18}O_{coral}$ proxies in the predictor matrix are related to both SST and SSS. Due to the disparities between observational/instrumental salinity datasets, as pointed out by Ref [68] and verified by our analysis in Figure S31, we adopt a value of a_1 of −0.22 ‰/°C based on empirical and theoretical estimates of the $\delta^{18}O_{coral}$–SST relationship[90]. The coefficient a_2 is defined, similarly to Ref [90], at each site by the regional slopes of the $\delta^{18}O_{sw}$–SSS relationship. Figure S6a in Ref [90] supplement is reproduced here in Figure S19 for quick reference. Equatorial tropical coral sites (6°N < latitude > 6°S) have a value of a_2 of 0.27 ‰/(g/g) and our remaining South Pacific coral sites have a value of a_2 of 0.45 ‰/(g/g). The coefficient, b, relates the Vienna Pee Dee Belemnite (VPDB) and Vienna Standard Mean Ocean Water reference standards for $\delta^{18}O_{carbonate}$ and $\delta^{18}O_{sw}$, respectively (b = 0.97002). The error term ε is determined similarly to Eq 1, using SNR = 4.0 and 1.0.

$$(Sr/Ca)_{coral} = c_1\, SST + \varepsilon \tag{1}$$

$$\delta^{18}O_{coral} = a_1\, SST + a_2\, SSS\, b + \varepsilon \tag{2}$$

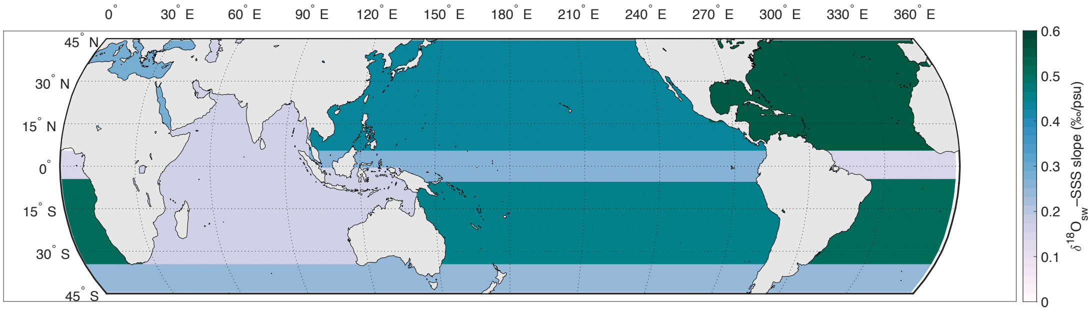

Figure S19 | Estimates of $\delta^{18}O_{sw}$–SSS slopes of the global oceans. Spatial distribution of a_2 ($\delta^{18}O_{sw}$–SSS slope in ‰/psu) adapted from Ref [90] (their Supplementary Figure S6a), which was based on the regional $\delta^{18}O_{sw}$–SSS slopes from Ref [67] and land/sea masks and ocean basin limits in CESM.

The following figures show the results of the pseudoproxy experiments for SNRs of 4.0 and 1.0, respectively. The evaluation metrics highlight the ability of the PCR method and our coral network to obtain skilful reconstructions when the SNR is 4.0, evaluated in both the same manner as the main reconstruction in this study, and with the extended evaluation available to the pseudoproxy experiment. In particular, all CE and CE_{det} values are positive (Figure S20b and Figure S21). The reduction in the number of available proxies going back in time, and particularly around 1825, leads to a modelled loss in skill as can be seen in the example timeseries of the evaluation statistics for CESM-LME ensemble member 1 (Figure S20b). However, the evaluation metrics remain positive for the entire reconstruction, and the summary statistics across all evaluation metrics indicate positive reconstruction skill. When the SNR is reduced to 1.0, that is, the signal and noise variance are of equal magnitude for all of the coral sites, the skill is reduced (Figure S22 and Figure S23). Some periods for some ensemble members indicate negative evaluation metrics. However, even at this high level of simulated proxy noise the median values for key metrics such as CE and CE_{det} remain positive. This provides further evidence of the robustness of the reconstruction method and the coral network employed in this study.

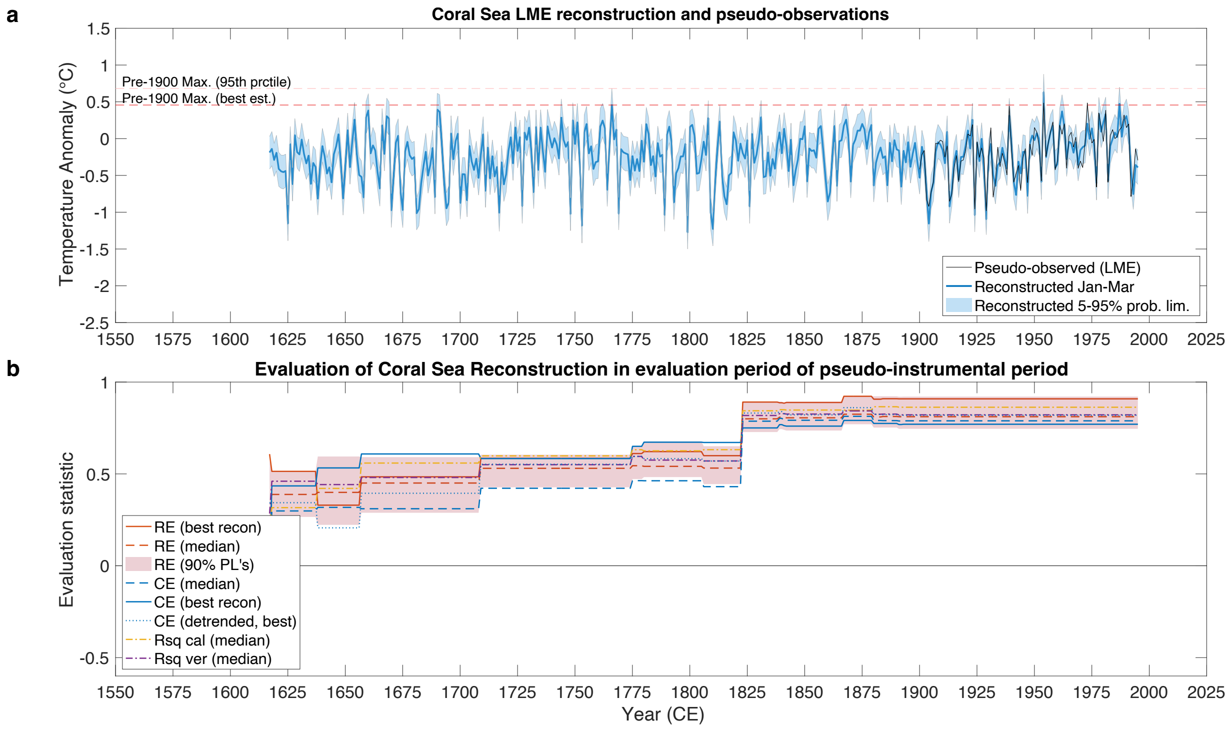

Figure S20 | Pseudoproxy experiment results for signal-to-noise ratio (SNR) of 4.0 for CESM-LME ensemble member 1. a, Timeseries of reconstructed (highest CE reconstruction in dark blue and 5–95th percentile probability limits in light blue shading) and pseudo-observed (black) Jan-Mar SSTa for the Coral Sea region in CESM-LME; **b,** Corresponding evaluation statistics.

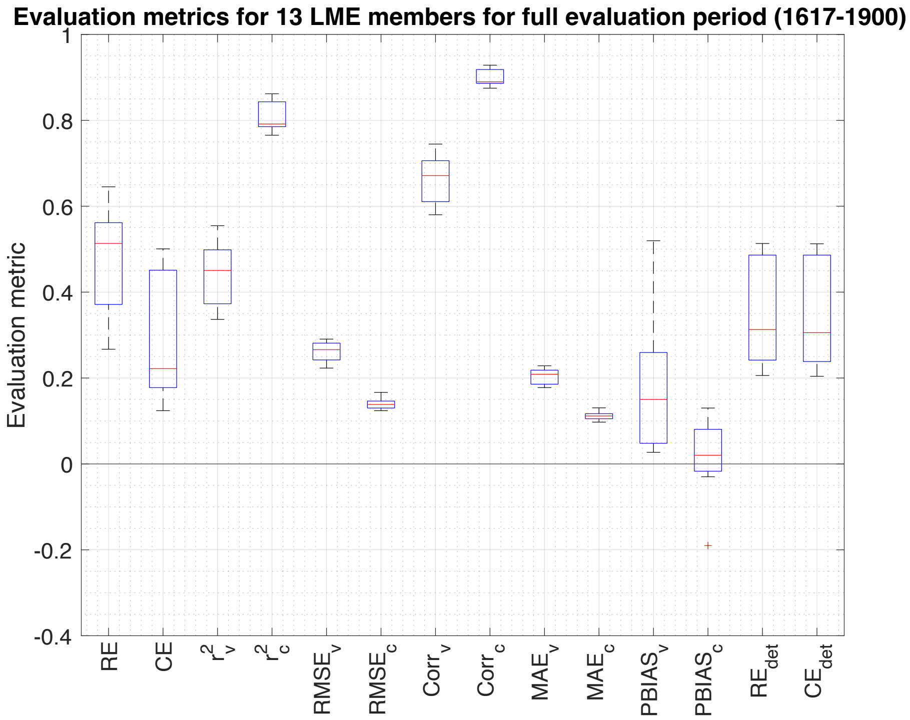

Figure S21 | Evaluation metrics for pseudoproxy experiment for SNR=4.0. Evaluation metrics for all 13 CESM-LME ensemble members. Statistics are shown for all CESM-LME ensemble members, evaluted over the full pre-observational period (1618–1899). Evalaution metrics are described in section 3.1. In each box plot, the red central horizontal line indicates the median; the bottom and top edges of the box indicate the 25th and 75th percentiles; the whiskers extend to the most extreme data points not considered outliers (shown as red crosses, values that are more than 1.5 times the interquartile range away from the bottom or top of the box).

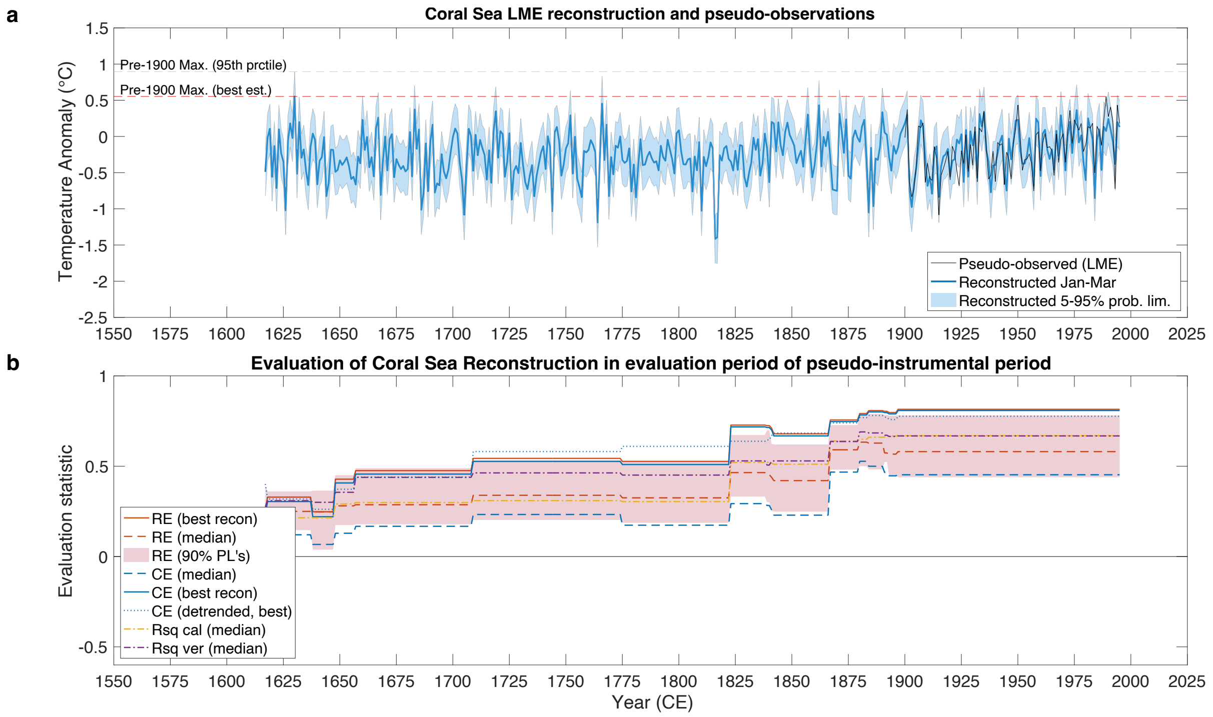

Figure S22 | Pseudoproxy experiment results for signal-to-noise ratio (SNR) of 1.0 for CESM-LME ensemble member 1. a, Timeseries of reconstructed (highest CE reconstruction in dark blue and 5–95th percentile probability limits in light blue shading) and pseudo-observed (black) Jan-Mar SSTa for the Coral Sea region in CESM-LME. **b,** Corresponding evaluation statistics.

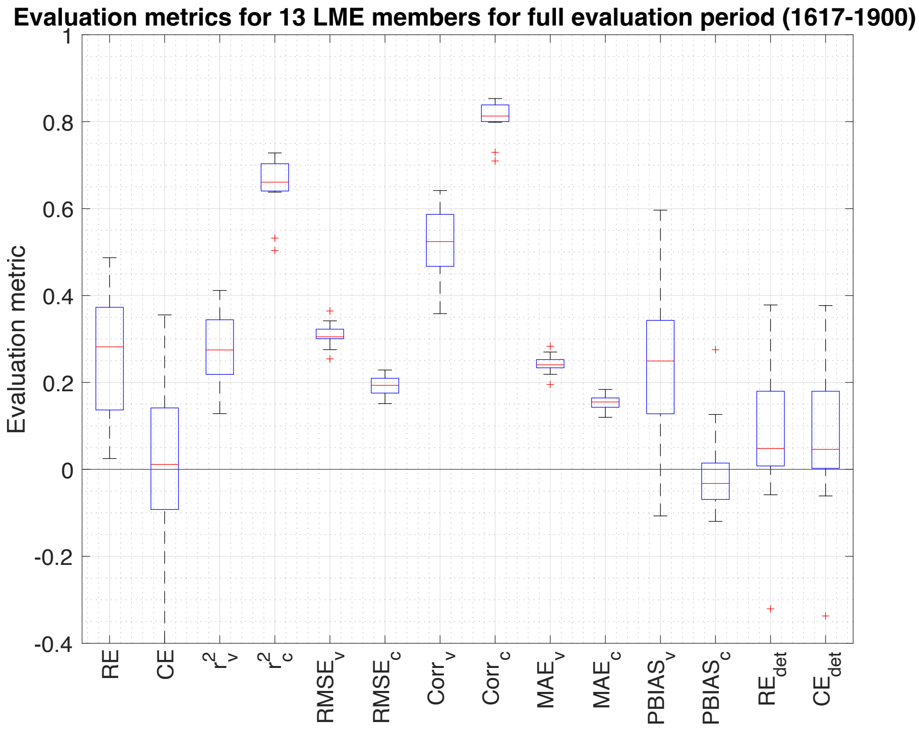

Figure S23 | Evaluation metrics for pseudoproxy experiment for SNR=1.0. Evaluation metrics for all 13 CESM-LME ensemble members. Statistics are shown for all CESM-LME ensemble members, evaluted over the full pre-observational period (1618–1899). Evalaution metrics are described in section 3.1. In each box plot, the red central horizontal line indicates the median; the bottom and top edges of the box indicate the 25th and 75th percentiles; the whiskers extend to the most extreme data points not considered outliers (shown as red crosses, values that are more than 1.5 times the interquartile range away from the bottom or top of the box).

4. Evaluation with independent datasets

4.1. Additional independent 5-year resolution record (Calvo et al. 2007)

A 5-year resolution record from Flinders Reef[24] provides an independent check of the results of this study. One drawback of this record is that the published data does not include a representation of uncertainty. This is why we have included the comparison in the Supplementary Information, rather than the main paper. Figure S4 corresponds to Figure 2b in the main paper but uses the 5-year resolution Flinders Reef (outer reef) record of Ref [24] rather than the nearshore Great Barrier Reef record of Ref [23]. The reconstruction shows a warming trend across the industrial era, similarly to the other reconstructions. A single data point in the early 1700s indicates a high SST anomaly at this site. Note that updated (higher resolution)

$\delta^{18}O$ and Sr/Ca Flinders Reef records from Ref [25] are included as predictors in our Coral Sea SSTa reconstruction.

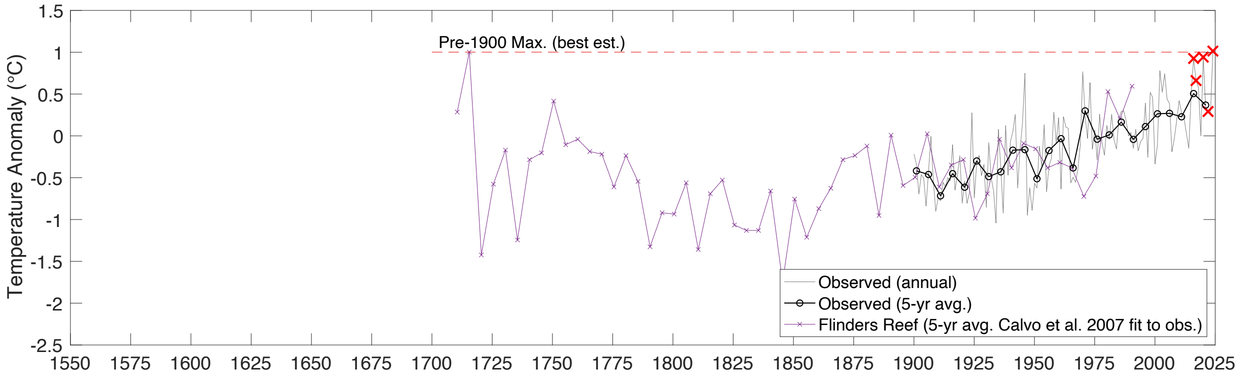

Figure S24 | Independent comparison of observed GBR SST and 5-year resolution record of Calvo et al (2007). Flinders Reef 5-year mean sea surface temperature anomaly series (purple) based on data from Calvo et al. (2007) and aligned with modern SSTa observations for the GBR region (ERSSTv5), shown at annual (thin grey line) and 5-yr resolutions (black line and open circles); dotted red horizontal line indicates best estimate of the pre-1900 maxima; the recent mass coral bleaching years are indicated with red crosses.

4.2. Independent evaluation with modern coral records from the GBR

Here we explore independent evaluation of our main results using modern coral records from within the GBR. There are numerous coral Sr/Ca records from within the GBR that do not extend further back than 1900, and most are a few years to a few decades in temporal coverage, and are therefore too short to meet the inclusion criteria for our multi-century Coral Sea SSTa reconstruction. Overall, these records do, however, cover some, or all, of the interval from 1970 to the 2010s[34,91–102]. Here we evaluate if recent warming is captured in the late 20th century-early 21st century GBR Sr/Ca records, in line with instrumental observations and the modern portion of our multi-century Coral Sea reconstruction. These short modern records bridge the gap between our Coral Sea SSTa reconstruction, which ends in the year 1995, and the more recent warming recorded in the satellite era instrumental records.

There are a total of 44 GBR coral Sr/Ca series in our comparison, all with relatively high temporal resolution (fortnightly to bi-monthly). The locations of the records are shown in Figure S25, and their metadata are shown in Table S3. We use the published relationships between Sr/Ca and SST (linear regression coefficients and intercepts shown in Table S3) to

infer timeseries of SST for each Sr/Ca series, focusing on Jan–Mar period. To aid interpretation, the sites are divided into the following three latitude groups:

- Northern GBR: < 18°S
- Central GBR: 18 – 21°S
- Southern GBR: > 21°S

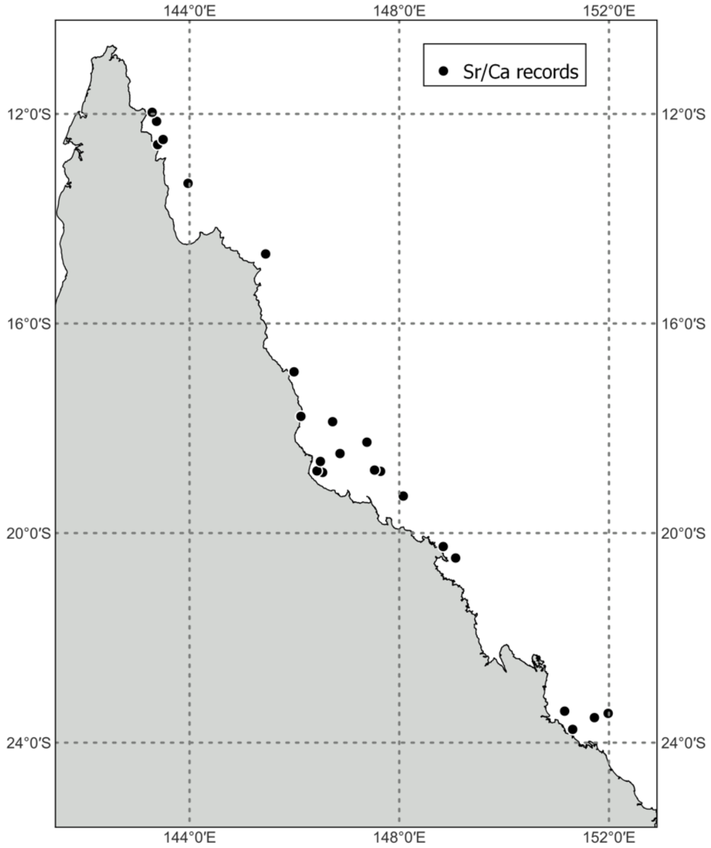

Figure S25 | Locations of modern coral Sr/Ca data from the GBR. These records do not meet the criteria for inclusion in the main Coral Sea SSTa reconstruction of this study (too short).

Table S3. Metadata for our modern (post-1900) coral Sr/Ca compilation from the GBR. Records and data are from the Great Barrier Reef Coral Skeletal Records Database (GBRCD)[34].

Dataset Reference	Reef SIte	GBR Region	Coral Species	Year From	Year To	Resolution	Reference	Reference DOI	Sr/Ca-SST Intercept	Sr/Ca-SST Slope
RE19GBR01	13-050	North	*Porites* sp.	1973	1990	Monthly	Reed et al. 2019	10.1007/s00338-018-01755-8	10.333	-0.049
DO18GBR01	17-065	North	*Porites lutea*	1990	1994	Bi-monthly	D'Olivo et al. 2018	10.1016/j.gca.2018.07.035	10.353	-0.052
RE19CLK01	Clerke Reef	North	*Porites* sp.	1992	2004	Monthly	Reed et al. 2019	10.1007/s00338-018-01755-8	10.4	-0.048
AL03DAV01	Davies Reef	Central	*Porites mayeri*	1989	1993	Fotnightly	Alibert et al. 2003	10.1016/S0016-7037(02)01055-4	10.46	-0.06
DO18DAV01	Davies Reef	Central	*Porites lutea*	1996	2009	Bi-monthly	D'Olivo et al. 2018	10.1016/j.gca.2018.07.035	10.261	-0.051
DO18DAV02	Davies Reef	Central	*Porites lobata*	2007	2013	Bi-monthly	D'Olivo et al. 2018	10.1016/j.gca.2018.07.035	10.136	-0.047
DO18DAV03	Davies Reef	Central	*Porites lobata*	2006	2013	Bi-monthly	D'Olivo et al. 2018	10.1016/j.gca.2018.07.035	10.053	0.042
FA03DAV02	Davies Reef	Central	*Porites* sp.	1990	1993	Fortnightly	Fallon et al. 2003	10.1007/s00338-003-0322-5	10.73	-0.0707
RE19EEL01	Eel Reef	North	*Porites* sp.	1979	1990	Monthly	Reed et al. 2019	10.1007/s00338-018-01755-8	10.445	-0.051
DO18FIT01	Fitzroy Island Reef	North	*Porites australiensis*	1990	1994	Bi-monthly	D'Olivo et al. 2018	10.1016/j.gca.2018.07.035	10.019	-0.042
DO18HAV01	Havannah Reef	Central	*Porites lutea*	1999	2008	Bi-monthly	D'Olivo et al. 2018	10.1016/j.gca.2018.07.035	10.149	-0.045
FA03HAV01	Havannah Reef	Central	*Porites* sp.	1992	1998	Weekly	Fallon et al. 2003	10.1007/s00338-003-0322-5	10.11	-0.0412
BR17HER01	Heron Reef	South	*Isopora* sp.	1971	1976	Monthly	Brenner et al. 2017	10.1002/2016pa002973	11.37	-0.083
BR17HER02	Heron Reef	South	*Isopora* sp.	1971	1976	Monthly	Brenner et al. 2017	10.1002/2016pa002973	11.37	-0.083
BR17HER03	Heron Reef	South	*Isopora* sp.	1971	1975	Monthly	Brenner et al. 2017	10.1002/2016pa002973	11.37	-0.083
BR17HER04	Heron Reef	South	*Isopora* sp.	2007	2012	Monthly	Brenner et al. 2017	10.1002/2016pa002973	11.37	-0.083
BR17HER05	Heron Reef	South	*Isopora* sp.	2010	2012	Monthly	Brenner et al. 2017	10.1002/2016pa002973	11.37	-0.083
SA16HER01b	Heron Reef	South	*Porites lutea*	2005	2014	Bi-monthly	Sadler et al. 2016	10.1002/2016pa002943	10.6672	-0.0651
SA16HER02b	Heron Reef	South	*Porites lobata*	2002	2014	Bi-monthly	Sadler et al. 2016	10.1002/2016pa002943	10.6672	-0.0651
SA16HER03b	Heron Reef	South	*Porites lutea*	2009	2014	Monthly	Sadler et al. 2016	10.1002/2016pa002943	10.6672	-0.0651
SA16HER04b	Heron Reef	South	*Porites lutea*	2010	2014	Bi-monthly	Sadler et al. 2016	10.1002/2016pa002943	10.6672	-0.0651
WU21HUM01	Hummocky Island	South	*Porites* sp.	1897	2017	Monthly	Wu et al. 2021	10.1016/j.scitotenv.2021.147393	10.097	–0.044
WU21HUM02	Hummocky Island	South	*Porites* sp.	1978	2017	Monthly	Wu et al. 2021	10.1016/j.scitotenv.2021.147393	10.196	–0.048
RO14KIN01	King Reefs	North	*Porites* sp.	1989	2008	Seasonal	Roche et al. 2014	10.1177/0959683614534739	9.964	0.044
DO18LIZ01	Lizard Island	North	*Porites australiensis*	2003	2013	Bi-monthly	D'Olivo et al. 2018	10.1016/j.gca.2018.07.035	10.274	-0.045
WU21MAS01a	Mast Head Reef	South	*Porites* sp.	1907	2017	Monthly	Wu et al. 2021	10.1016/j.scitotenv.2021.147393	10.191	–0.053
DO18MYR01	Myrmidon Reef	Central	*Porites solida*	1996	2000	Bi-monthly	D'Olivo et al. 2018	10.1016/j.gca.2018.07.035	10.581	-0.054
DO18MYR02	Myrmidon Reef	Central	*Porites lutea*	2003	2012	Bi-monthly	D'Olivo et al. 2018	10.1016/j.gca.2018.07.035	10.05	-0.038
DO18MYR03	Myrmidon Reef	Central	*Porites lutea*	2005	2012	Bi-monthly	D'Olivo et al. 2018	10.1016/j.gca.2018.07.035	10.562	-0.06
DO18MYR04	Myrmidon Reef	Central	*Porites lutea*	2004	2009	Bi-monthly	D'Olivo et al. 2018	10.1016/j.gca.2018.07.035	9.933	-0.039
MA00MYR01	Myrmidon Reef	Central	*Porites* sp.	1973	1996	Monthly	Marshall 2000; Marshall & McCulloch 2002	10.1016/S0016-7037(02)00926-2	10.4	-0.0575
RA17MYR01	Myrmidon Reef	Central	*Isopora* sp.	2001	2013	Monthly	Razak et al. 2017	10.1007/s00227-017-3099-8	10.44	-0.0512
RA17MYR02	Myrmidon Reef	Central	*Isopora* sp.	2001	2013	Monthly	Razak et al. 2017	10.1007/s00227-017-3099-8	10.44	-0.0512
RE19NOM01	Nomad Reef	North	*Porites* sp.	1990	2008	Monthly	Reed et al. 2019	10.1007/s00338-018-01755-8	10.64	-0.057
FA03ORP01	Orpheus Reef	Central	*Porites* sp.	1989	1992	Fortnightly	Fallon et al. 2003	10.1007/s00338-003-0322-5	10.23	-0.052
GA98ORP01	Orpheus Reef	Central	*Porites lobata*	1970	1994	Weekly	Gagan et al. 1998; Gagan et al. 2002	10.1126/science.279.5353.1014; 10.1029/2002GL015336	10.73	-0.0639
AL03PAN01	Pandora Reef	Central		1993	1998	Fortnightly	Alibert et al. 2003	10.1016/S0016-7037(02)01055-4	10.29	-0.0537
RE19POR01	Portland Roads	North	*Porites* sp.	1975	1990	Monthly	Reed et al. 2019	10.1007/s00338-018-01755-8	10.23	-0.045
DO18RIB01	Rib Ref	Central	*Porites mayeri*	1996	2008	Bi-monthly	D'Olivo et al. 2018	10.1016/j.gca.2018.07.035	10.235	-0.049
WU21SHW01	Shaw Island	Central	*Porites* sp.	1949	2017	Monthly	Wu et al. 2021	10.1016/j.scitotenv.2021.147393	9.991	–0.047
WU21SEN01	South End Curtis Island	South	*Porites* sp.	1901	2017	Monthly	Wu et al. 2021	10.1016/j.scitotenv.2021.147393	10.502	–0.061
WU21SMI01	South Molle Island	Central	*Porites* sp.	1975	2017	Monthly	Wu et al. 2021	10.1016/j.scitotenv.2021.147393	9.798	–0.042
MA00SLY01	Stanley Reef	Central	*Porites* sp.	1995	1999	Fortnightly	Marshall 2000; Marshall & McCulloch 2002	10.1016/S0016-7037(02)00926-2	10.4	-0.0587
FA03WHE01	Wheeler Reef	Central	*Porites* sp.	1982	1985	Weekly	Fallon et al. 2003	10.1007/s00338-003-0322-5	10.35	-0.0595

We firstly examine the individual timeseries of coral Sr/Ca-derived SST from each site in our modern coral data compilation. The Sr/Ca SST estimates in the individual months of Jan, Feb and Mar are extracted for the Northern, Central and Southern GBR groups (Figure S26, Figure S27 and Figure S28, respectively). Where these series are at a higher than monthly resolution

the individual coral series are averaged to produce a single value for each month of the year. The SST trend across the length of each dataset is shown in °C per decade, along with the p-value. We quote a °C per decade value to assist with identifying positive and negative trends in the GBR records. However, even though coral Sr/Ca SST estimates do appear to capture SST variations across latitudinal ranges[103] inter-coral variability could influence the magnitude of these estimates[104].

Individually, most of the available datasets are too short for analysis and interpretation of multidecadal SST trends, and cover different time periods, noting that 77% of the records show a positive trend over their length (34 out of 44 records). Four of the individual records do extend continuously back from 2017 to 1970. Average Jan–Mar Sr/Ca-based SST estimates for these datasets indicate a consistent long-term warming trend. The warming trends are statistically significant over their period of data availability (Figure S29), except for the shorter of the series in these comparisons (WU21SHW01), with a p-value (marginally) below the 5% significance level.

We further examine the data in our compilation by compositing all the individual Jan–Mar mean SST site series into the three latitudinal groups (Figure S30). Note that the series from nearshore King Reef[93] is included as part of the northern GBR latitude group but is excluded from our composite as it has variable sampling resolution (4-10 samples per year) making it difficult to apply SST-conversion equations and consistently extract the Jan–Mar season. These simple Northern, Central and Southern GBR composites, despite the spatial and temporal sparseness of the available GBR coral data, show that the coral-derived Jan–Mar SSTs reveal coherent evidence of SST warming from in-situ GBR coral data. The warming trends for the Central and Southern GBR are statistically significant, whereas the trend for the Northern GBR composite series has a p-value of 0.12.

Overall, our modern coral data compilation represents a valuable in-situ data source that agrees with instrumental observations and the modern portion of the broader Coral Sea region multi-century reconstruction. The composite and individual coral-based SST timeseries longer than 40 years show statistically significant warming trends in the GBR. The statistical significance of warming trends increases as the observational length and number of contributing datasets increases. These results provide supporting evidence for 20th and 21st Century warming on the GBR and the detection of these trends in coral geochemical data, aligning with the main results in this study.

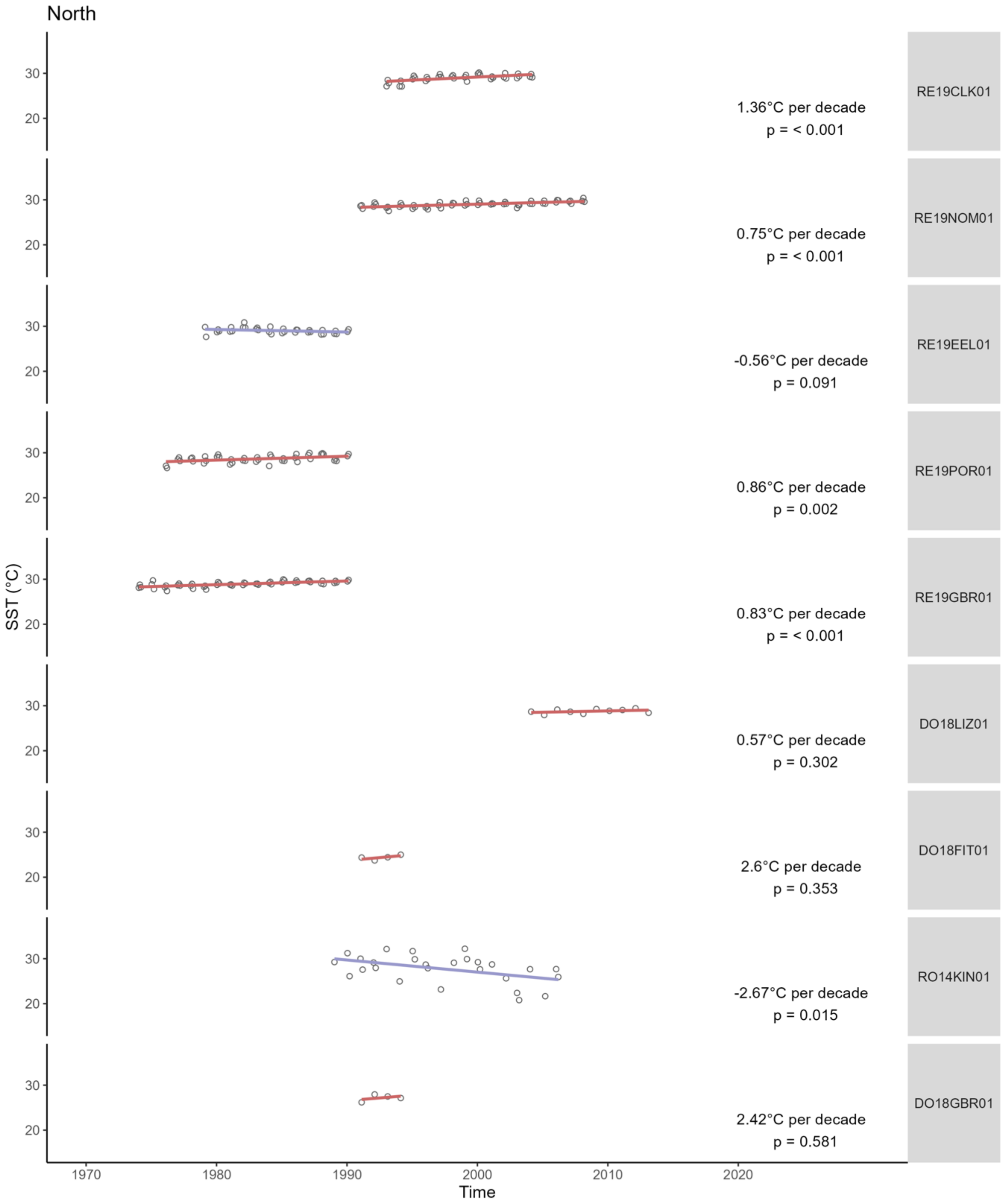

Figure S26 | Coral-derived SST estimates at individual sites in the *Northern* GBR. Results from our modern coral compilation for individual months of Jan, Feb and Mar; red trend lines indicate positive SST trends and blue lines indicate negative trends; Slope estimates and p-values are indicated for each record; Dataset ID abbreviations on right column are as shown in Table S3.

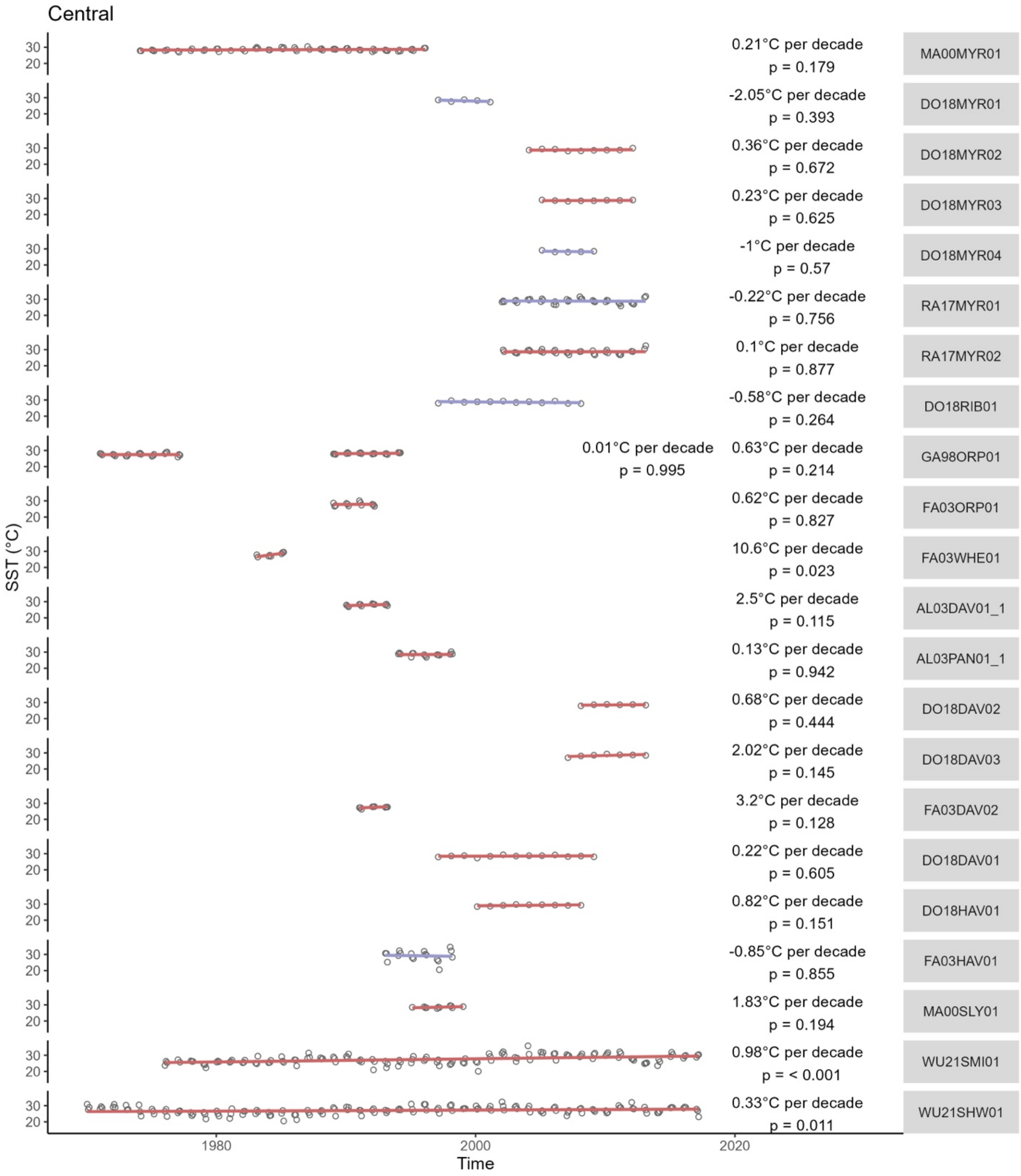

Figure S27 | Coral-derived SST estimates and trends at individual sites in the *Central* GBR. Results from our modern coral compilation for individual months of Jan, Feb and Mar; red trend lines indicate positive SST trends and blue lines indicate negative trends; Slope estimates and p-values are indicated for each record; Dataset ID abbreviations on right column are as shown in Table S3.

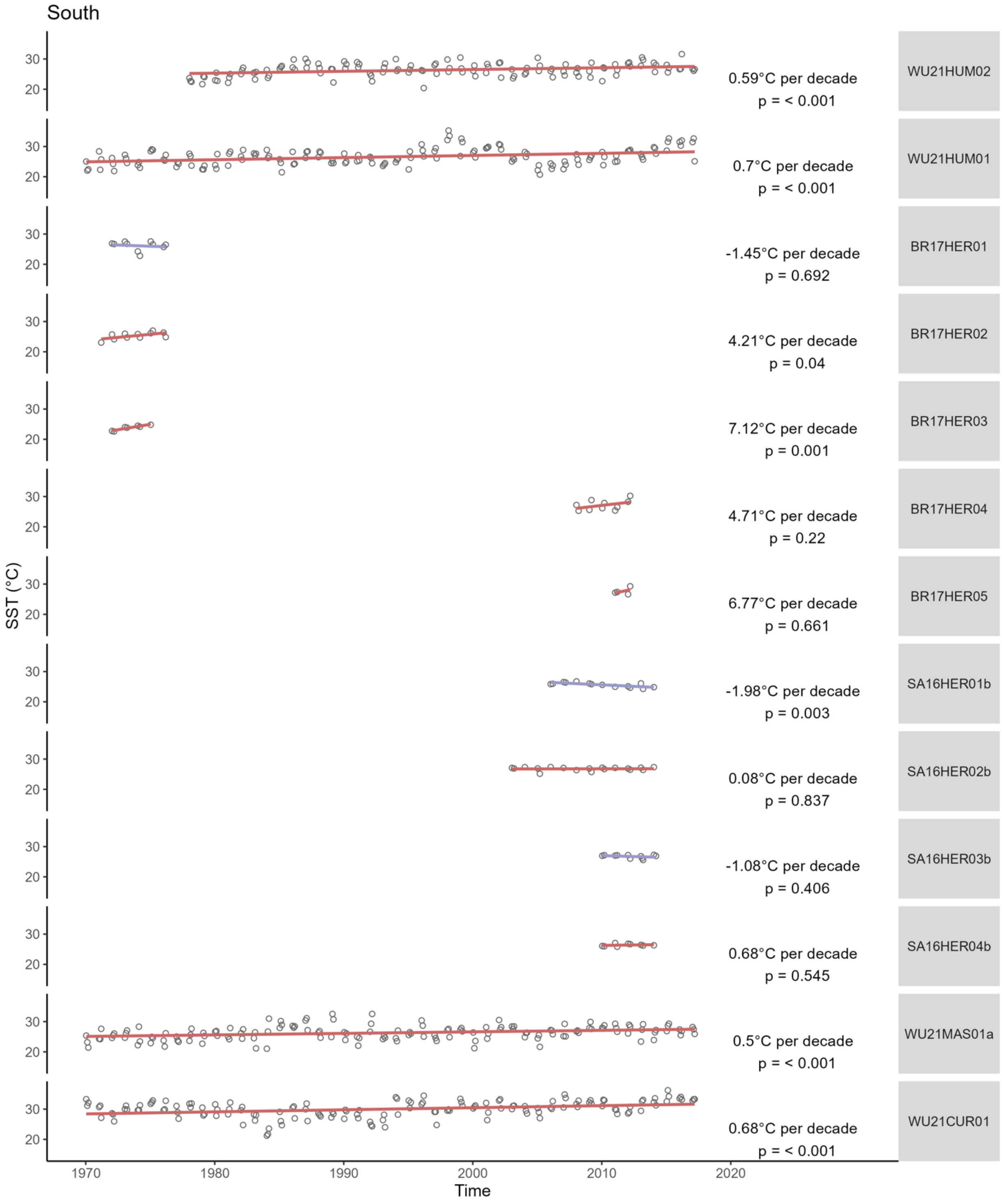

Figure S28 | Coral-derived SST estimates and trends at individual sites in the *Southern* GBR. Results from our modern coral compilation for individual months of Jan, Feb and Mar; red trend lines indicate positive SST trends and blue lines indicate negative trends; slope estimates and p-values are indicated for each record; Dataset ID abbreviations on right column are as shown in Table S3.

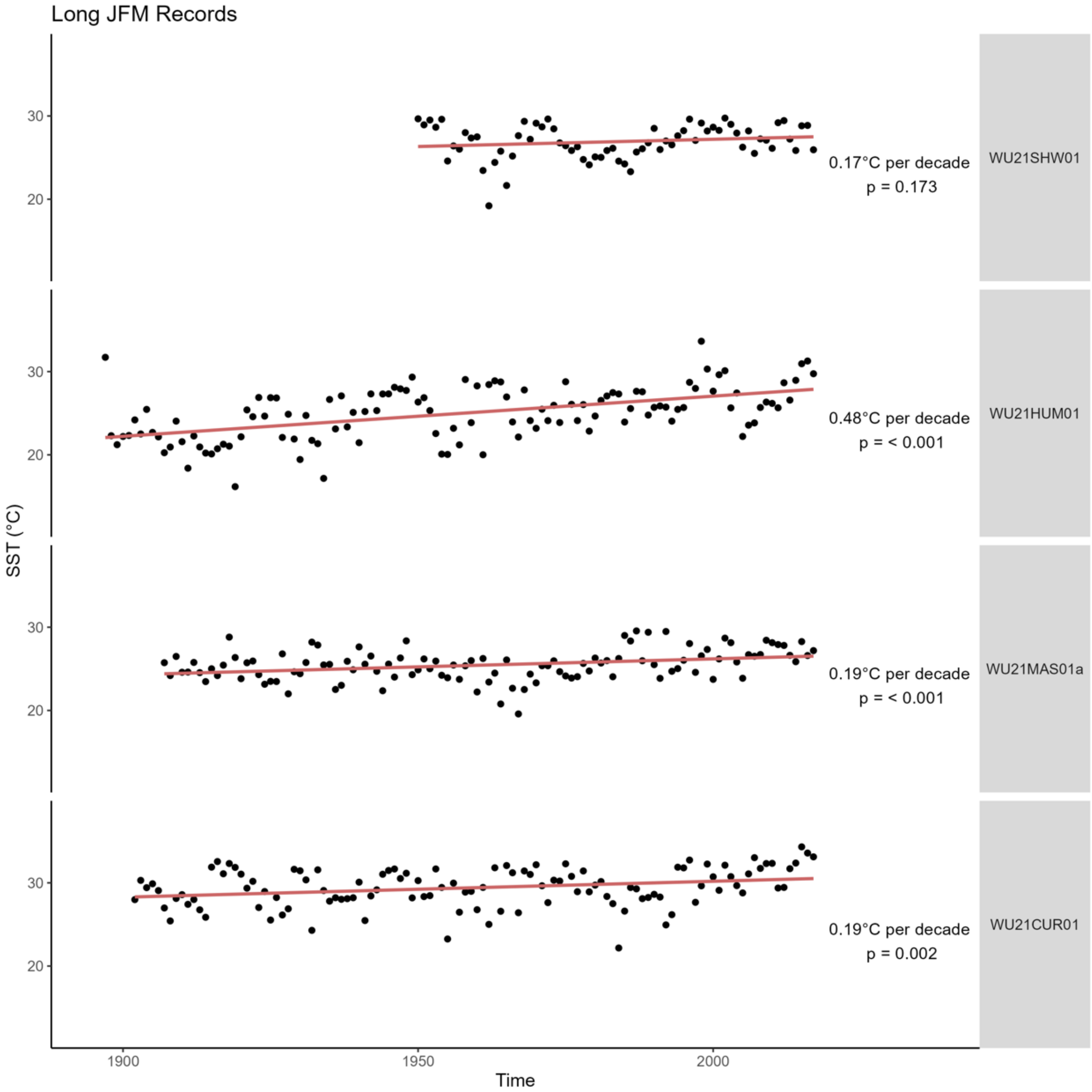

Figure S29 | Estimates for Jan–Mar mean SST and trends for long individual records. Results from our modern coral compilation for the subset of long (extending back beyond 1970) records in our modern coral data compilation. Red trend lines indicate positive SST trends and blue lines indicate negative trends; slope estimates and p-values are indicated for each record; Dataset ID abbreviations on right column are as shown in Table S3.

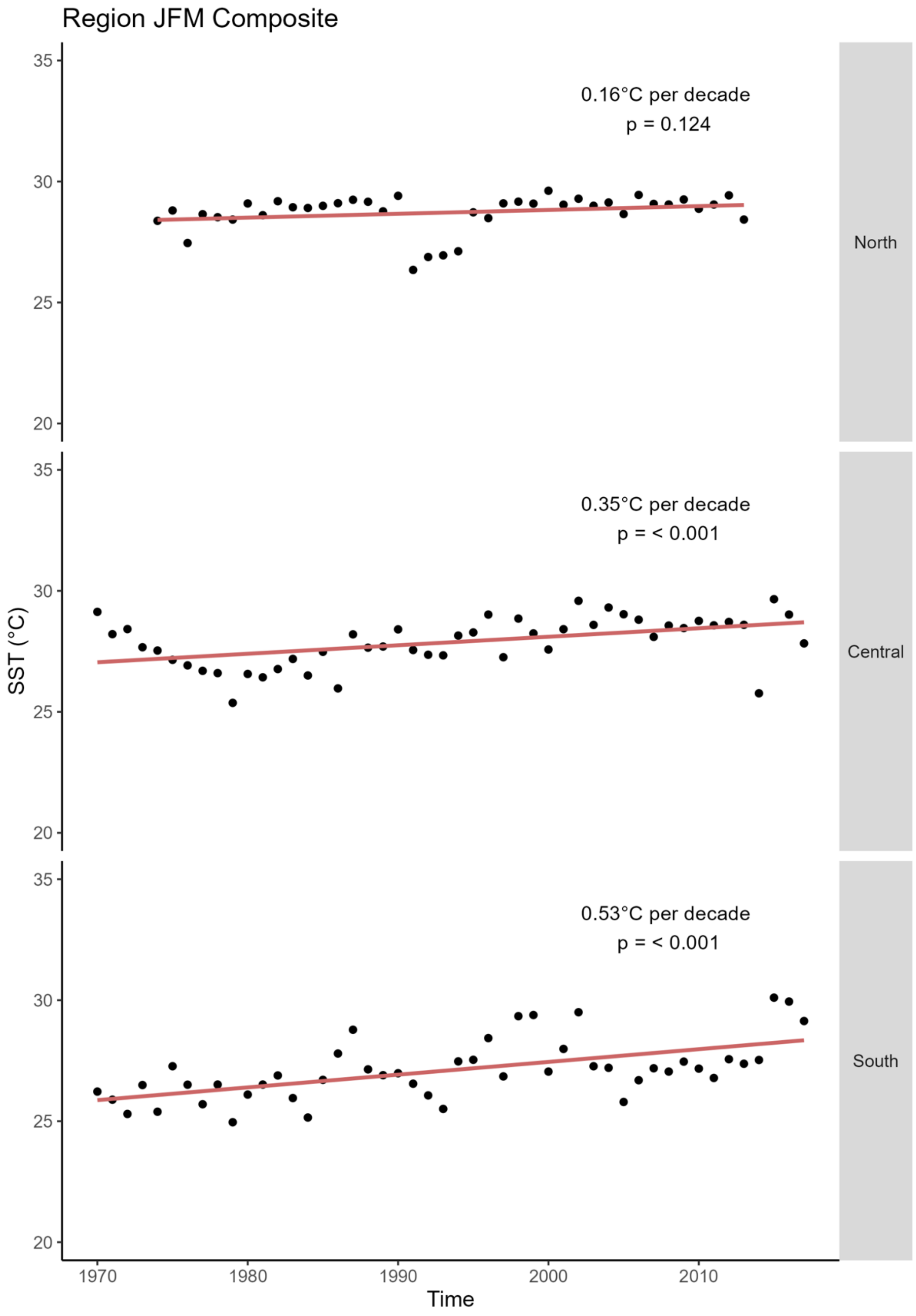

Figure S30 | Regional composite inferred Jan–Mar SST and trends. Results from our modern coral compilation for Sr/Ca records from the Northern, Central and Southern GBR, region indicated in the right column of the figure; red trend lines indicate positive SST trends and blue lines indicate negative trends; slope estimates and p-values are indicated for each record.

5. Reconstruction sensitivity to non-SST parameters

5.1. Analysis of possible confounding impacts of salinity on reconstruction

Here we explore the strength of the correlation between the highest CE reconstruction (using the full network) and instrumental SST and SSS datasets. The aim here is to both evaluate the Jan–Mar SSTa reconstruction against two different spatial SST datasets (ERSSTv5 and HadISST1.1) and to quantify the strength of the link between our reconstruction and the salinity field using three SSS datasets (SODA 3.3.1, ORAS5 and IAP).

Our reconstruction is statistically significantly correlated with both the target (ERSSTv5) and the non-target (HadISST1.1) SST datasets in the Coral Sea region and further afield (Figure S31a–b). The correlations with SST are significant and homogenous over large areas. In contrast, correlations between the SST reconstruction and SSS are either not statistically significant or inconsistent over the Coral Sea (Figure S31c–f), and the sign of the correlations varies across the domain. For the SODA 3.3.1 dataset (covering 1980–1996, Figure S31c), there are almost no areas of statistically significant correlation between our SST reconstruction and SSS. For the ORAS5 dataset (1979–1996, Figure S31d), and for the IAP dataset (for both the 1940–1996 and 1980–1996 periods, Figure S31e–f), around 15–20% of the Coral Sea region has a statistically significant negative correlation between the highest CE Coral Sea SSTa reconstruction and the gridded SSS data during Jan–Mar. With limited direct SSS observations and disparities between the temporal coverage and the methodologies used to derive the SSS datasets, reconciling discrepancies between results based on different SSS datasets remains a challenge[68]. We conclude, in the absence of longer SSS datasets and greater consistency between them, that SSS has a negligibly weak relationship with our Coral Sea SST reconstruction and that our methodology therefore successfully isolates the SST signal from any strong impacts from non-SST $\delta^{18}O_{sw}$ variations that co-vary with SSS.

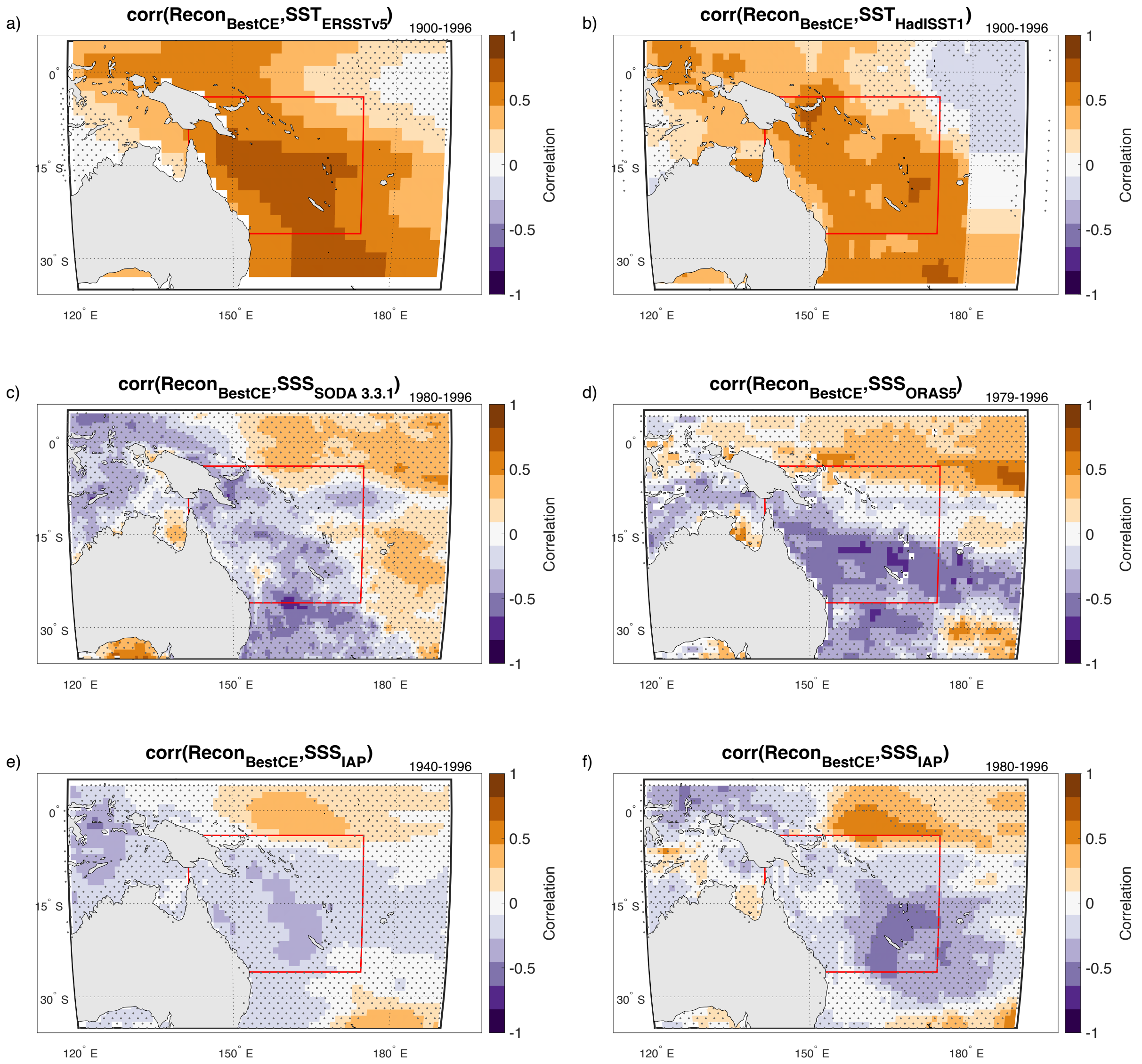

Figure S31 | Correlation between our reconstruction and observed SST/SSS. a–e, Correlation between the best reconstruction of Coral Sea Jan–Mar SSTa (highest CE, ERSSTv5 target) and the mean Jan–Mar SST and SSS from instrumental/reanalysis datasets (as shown in the panel titles); correlations computed over the common periods as stated in the upper right of each panel. Grey stippling indicates the *absence* of statistical significance at the 5% significnace level (p>0.05). Instrumental gridded datasets are: a) ERSSTv5 (1854–1996), b) HadISST1 (1870-1996), c) SODA 3.3.1 (1980–1996), d) ORAS5 (ensemble member 0, 1979–1996), e) SSS IAP (1940–1996) and e) IAP shortened to 1980–1996.

5.2. *Sensitivity to proxy network*

This section presents the results from a series of sensitivity tests which evaluate the influence of different subsets of coral data on the key conclusions of this study. The Coral Sea SSTa reconstructions remain skilful for their entire duration and the recent thermal extremes in our primary instrumental dataset (ERSSTv5) exceed the upper confidence limit of the reconstructed pre–1900 Jan–Mar SSTa in the Coral Sea (Figure S32–Figure S44).

5.2.1. *Sr/Ca network*

The six available Sr/Ca records (alone) are used in this Sr/Ca-only sensitivity test. Figure S32 and Figure S33 are otherwise the same as Figures 2a–c and 3 in the main paper (which uses the full network of 22 records).

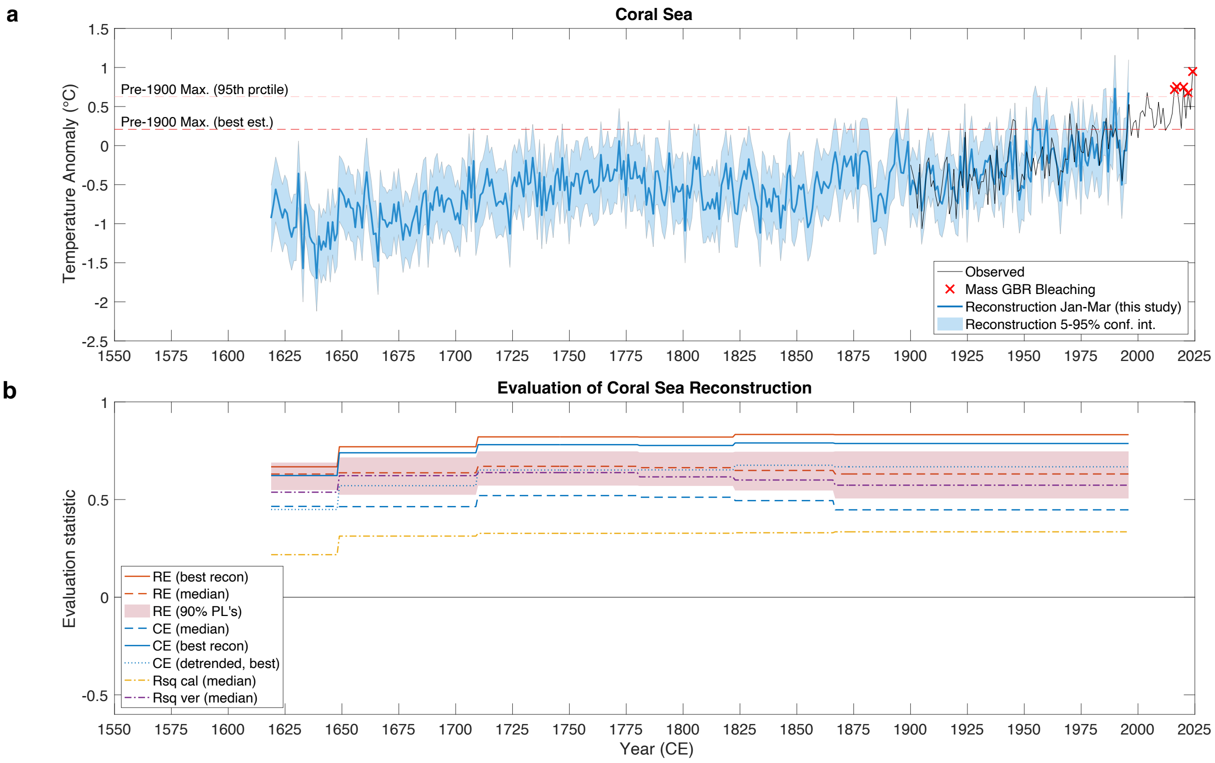

Figure S32 | Multi-century reconstruction of sea surface temperatures using the Sr/Ca network. As for Fig 2a and 2c in the main text, but using the six Sr/Ca records only for the reconstruction. **a,** Coral Sea reconstructed and observed mean sea surface temperature anomalies, relative to 1961–1990, for the period 1618–2024; highest skill (maximum CE) reconstruction with the Sr/Ca network shown in dark blue; 5–95th percentile reconstruction uncertainty shown in lighter blue shading; observed (ERSSTv5) data shown in black; recent

five mass bleaching events shown with red crosses; dotted horizontal lines indicate the best estimate (highest skill) and 95th percentile uncertainty bound for the maximum pre-1900 SSTa; **b,** Coral Sea reconstruction evaluation metrics (Supplementary Section 3.1);

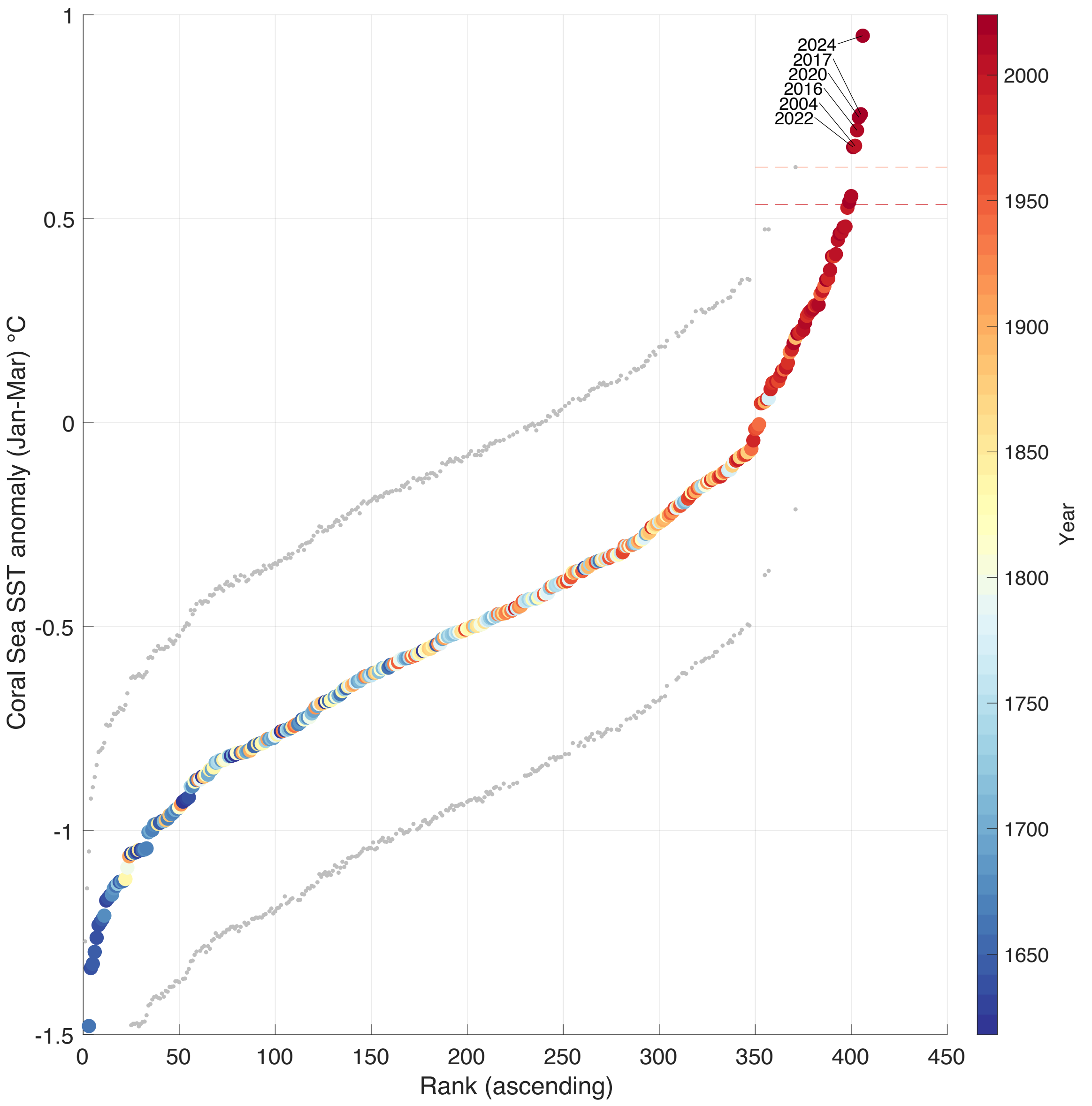

Figure S33 | Exceptional nature of recent Coral Sea surface temperatures using the Sr/Ca network. As for Fig 3 in the main text but using the six Sr/Ca series only. Ranked sea surface temperature anomalies, relative to 1961–1990 (coloured circles) for Jan–Mar for 1618–2024 for the best estimate (highest skill, Sr/Ca coral series) reconstruction (1618-1899) and instrumental (ERSSTv5) data (1900–2024); year indicated by colour of filled circles; 5–95th uncertainty in pre-1900 reconstructed temperature anomalies shown with small grey dots; year labels indicate warmest six years on record, five of which were mass coral bleaching events on

the GBR; light red (upper) dashed line indicates the upper 95th percentile uncertainty bound of the maximum pre-1900 reconstructed sea surface temperature anomaly; dark red (lower) dashed line indicates the 90th percentile limit.

5.2.2. Long network

The five longer records which all cover at least the period 1700–1900 CE are used in this sensitivity test. The two figures here are otherwise the same as Figure 2a–c and Figure 3 in the main paper (which uses the full network of 22 records).

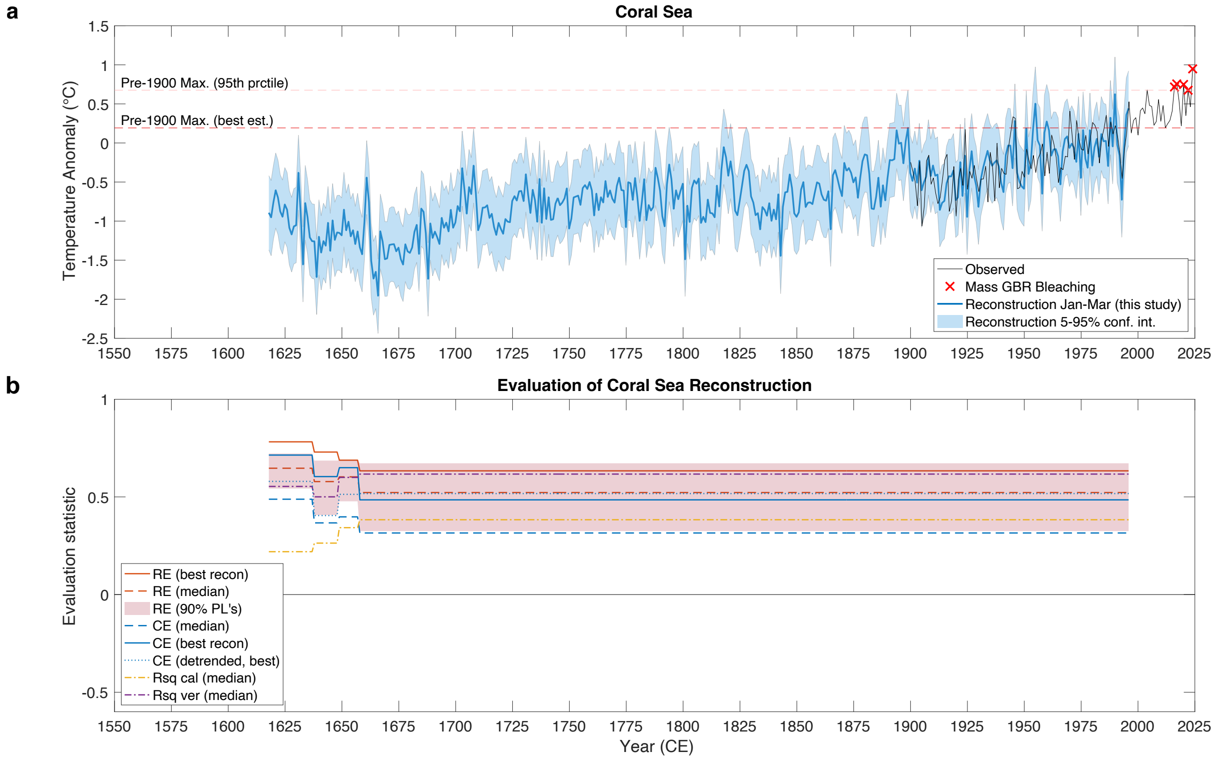

Figure S34 | Multi-century reconstruction of sea surface temperatures using Long network. As for Fig 2a and 2c in the main paper but using the five coral series from the Long network only. **a,** Coral Sea reconstructed and observed mean sea surface temperature anomalies, relative to 1961–1990, for the period 1618–2024; highest skill (maximum CE) reconstruction with the Long proxy network shown in dark blue; 5–95th percentile reconstruction uncertainty shown in lighter blue shading; observed (ERSSTv5) data shown in black; recent five mass bleaching events shown with red crosses; dotted horizontal lines indicate the best estimate (highest skill) and 95th percentile uncertainty bound for the maximum pre-1900 SSTa; **b,** Coral Sea reconstruction evaluation metrics (Supplementary Section 3.1);

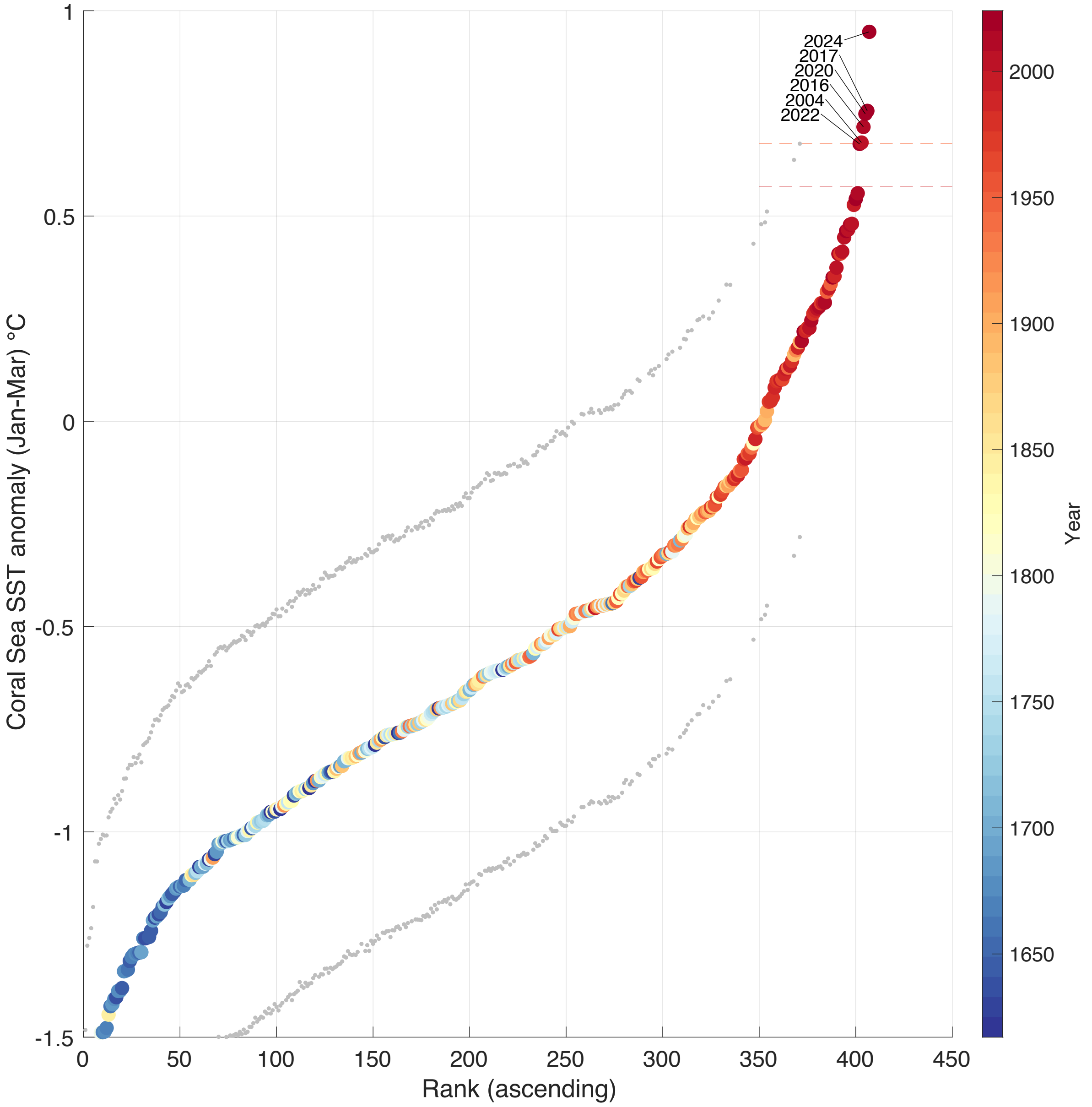

Figure S35 | Exceptional nature of recent Coral Sea surface temperatures using the Long network. As for Fig 3 in the main text but using the five coral series from the Long network only. Ranked sea surface temperature anomalies, relative to 1961–1990 (coloured circles) for Jan–Mar for 1618–2024 for the best estimate (highest skill, Long network) reconstruction (1618-1899) and instrumental (ERSSTv5) data (1900–2024); year indicated by colour of filled circles; 5–95th uncertainty in pre-1900 reconstructed temperature anomalies shown with small grey dots; year labels indicate warmest six years on record, five of which were mass coral bleaching events on the GBR; light red (upper) dashed line indicates the upper 95th percentile uncertainty bound of the maximum pre-1900 reconstructed sea surface temperature anomaly; dark red (lower) dashed line indicates the 90th percentile limit.

5.2.3. Best-10 proxies

This sensitivity test uses the ‘best’ network of records, that is, the network of records which have the highest overall CE when the number of records included in the reconstruction is varied between 1–22 (and incorporated in a stepwise manner in order of highest to lowest correlation with the ERSSTv5 target, see Section 5.2.5). The subset with the highest CE corresponds to the proxy subset with the 10 highest correlated proxy series. The two figures here are otherwise the same as Figures 2a–c and 3 in the main paper (which uses the full network of 22 records).

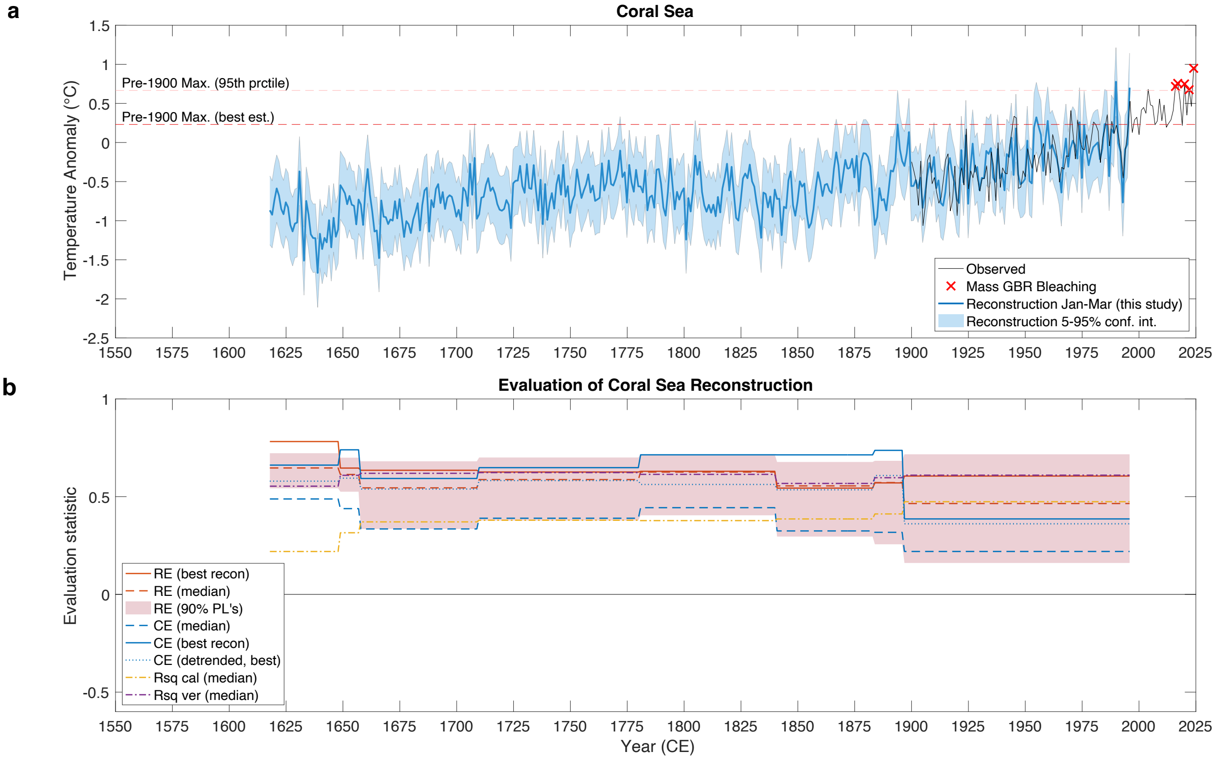

Figure S36 | Multi-century reconstruction of sea surface temperatures using Best-10 network. As for Fig 2a and 2c in the main paper but using the ten coral series from the Best-10 network only. **a,** Coral Sea reconstructed and observed mean sea surface temperature anomalies, relative to 1961–1990, for the period 1618–2024; highest skill (maximum CE) reconstruction with the Best-10 proxy network shown in dark blue; 5–95th percentile reconstruction uncertainty shown in lighter blue shading; observed (ERSSTv5) data shown in black; recent five mass bleaching events shown with red crosses; dotted horizontal lines indicate the best estimate (highest skill) and 95th percentile uncertainty bound for the maximum pre-1900 SSTa; **b,** Coral Sea reconstruction evaluation metrics (Supplementary Section 3.1);

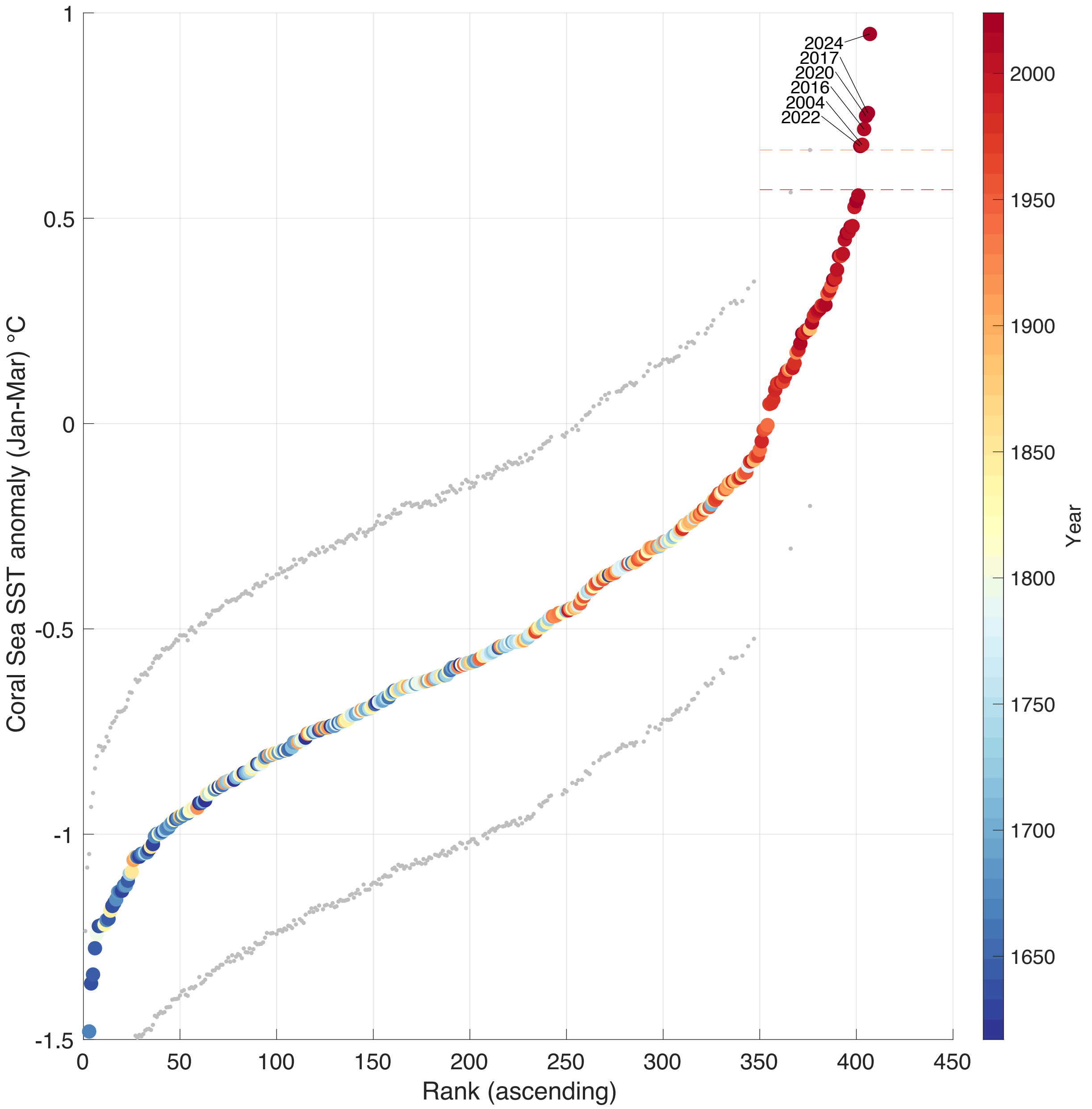

Figure S37 | Exceptional nature of recent Coral Sea surface temperatures using the Best-10 network. As for Fig 3 in the main paper but using the ten coral series from the Best-10 network only. Ranked sea surface temperature anomalies, relative to 1961–1990 (coloured circles) for Jan–Mar for 1618–2024 for the best estimate (highest skill, Best-10 network) reconstruction (1618-1899) and instrumental (ERSSTv5) data (1900–2024); year indicated by colour of filled circles; 5–95th uncertainty in pre-1900 reconstructed temperature anomalies shown with small grey dots; year labels indicate warmest six years on record, five of which were mass coral bleaching events on the GBR; light red (upper) dashed line indicates the upper

95th percentile uncertainty bound of the maximum pre-1900 reconstructed sea surface temperature anomaly; dark red (lower) dashed line indicates the 90th percentile limit.

5.2.4. OmitBioMed

This sensitivity test omits records from the full network for which there is a cited potential for biological mediation of the records, and also those records which have either an unexpected (i.e. positive) or lack of a statistically significant correlation with the target. The six omitted records and the reasons for testing their exclusion here are as follows:

1. Espiritu Santo, $\delta^{18}O$, Quinn et al., 1996 (positive correlation with target)
2. Kavieng, Sr/Ca, Alibert et al., 2008 (potential biological mediation/non-climatic artefacts)
3. Nauru-2, $\delta^{18}O$, Guilderson et al., 1999 (potential biological mediation/non-climatic artefacts)
4. Rabaul, $\delta^{18}O$, Quinn et al., 2006 (potential biological mediation/non-climatic artefacts)
5. Rabaul, Sr/Ca, Quinn et al., 2006 (potential biological mediation/non-climatic artefacts)
6. Tarawa Atoll, $\delta^{18}O$, Cole et al., 1993 (no significant correlation with target)

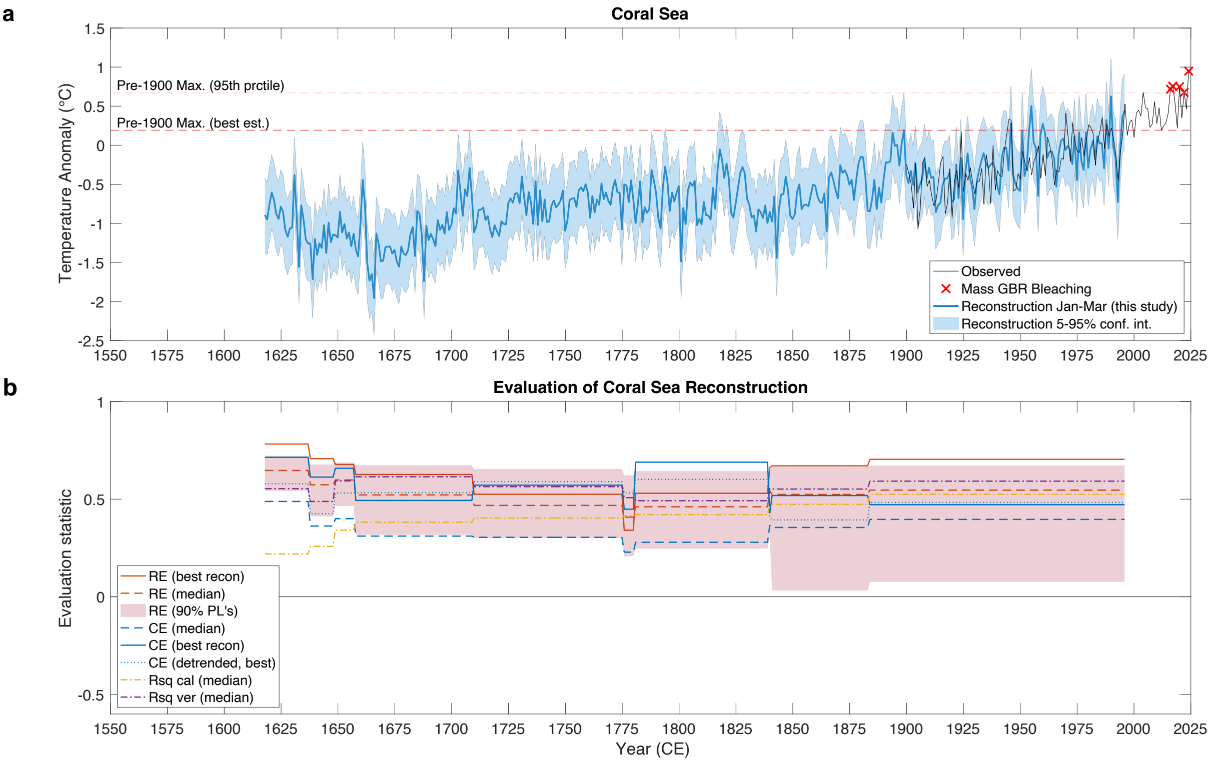

Figure S38 | Multi-century reconstruction of sea surface temperatures using OmitBioMed network. As for Fig 2 a–c in the main paper but using the 16 coral series from the *OmitBioMed* network only. **a,** Coral Sea reconstructed and observed mean sea surface

temperature anomalies, relative to 1961–1990, for the period 1618–2024; highest skill (maximum CE) reconstruction with the OmitBioMed proxy network shown in dark blue; 5–95th percentile reconstruction uncertainty shown in lighter blue shading; observed (ERSSTv5) data shown in black; recent five mass bleaching events shown with red crosses; dotted horizontal lines indicate the best estimate (highest skill) and 95th percentile uncertainty bound for the maximum pre-1900 SSTa; **b,** Coral Sea reconstruction evaluation metrics (Supplementary Section 3.1);

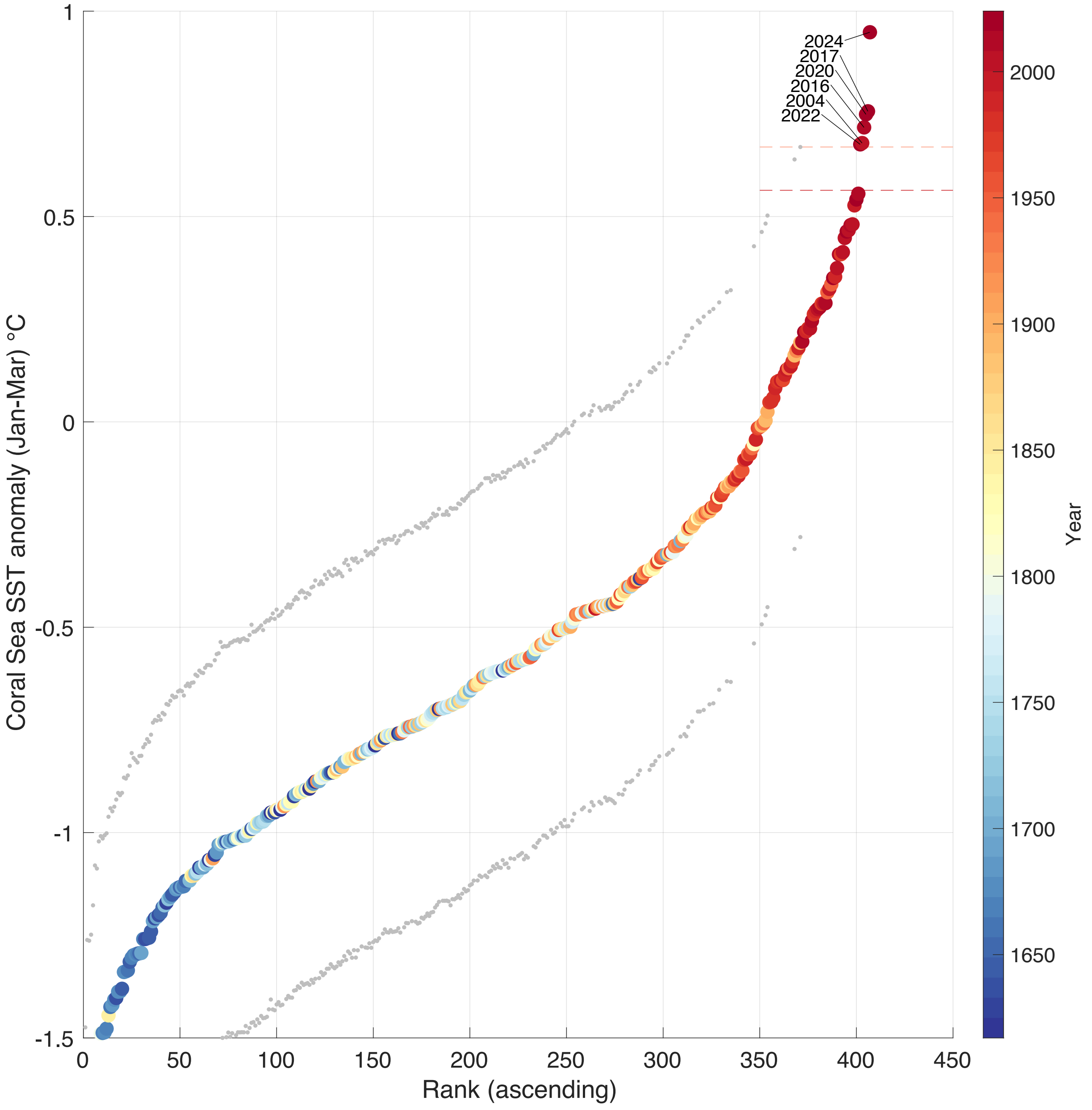

Figure S39 | Exceptional nature of recent Coral Sea surface temperatures using the OmitBioMed network. As for Fig 3 in the main paper but using the 10 records from the

OmitBioMed network only. Ranked sea surface temperature anomalies, relative to 1961–1990 (coloured circles) for Jan–Mar for 1618–2024 for the best estimate (highest skill, OmitBioMed network) reconstruction (1618-1899) and instrumental (ERSSTv5) data (1900–2024); year indicated by colour of filled circles; 5–95th uncertainty in pre-1900 reconstructed temperature anomalies shown with small grey dots; year labels indicate warmest six years on record, five of which were mass coral bleaching events on the GBR; light red (upper) dashed line indicates the upper 95th percentile uncertainty bound of the maximum pre-1900 reconstructed sea surface temperature anomaly; dark red (lower) dashed line indicates the 90th percentile limit.

5.2.5. Proxy network perturbation

Here we perturb the proxy network and perform an ensemble of Coral Sea SSTa (Jan–Mar) reconstructions using subsets of our proxy network ranging from 1 proxy through to all 22 proxies. The aim here is to objectively and systematically assess whether the exclusion of gradually lower correlated records affects the key findings of our study. In each subset we include the n_{pert} proxies (with n_{pert} ranging from 1 to 22) in order from highest to lowest correlation with the target (this order is shown in Table S4). For example, for the reconstruction with 10 proxies, we include the top 10 records in Table S4; note that this subset is identical to the 'Best-10' sensitivity test described earlier.

For each of these 22 sets of reconstructions, we:

(i) evaluate the reconstructions and compare the CE, RE, detrended CE and detrended RE of the highest skill reconstruction (maximum time integrated CE) across all 22 proxy networks;

(ii) quantify our reconstruction error (using the method described in the main text of this study); and

(iii) compare the highest reconstructed pre–1900 SSTa with recent high SSTa values (during GBR mass bleaching years) to test whether our finding, that recent thermal extremes are unusually high with respect to the last four centuries of reconstructed Coral Sea SSTa, is sensitive to changes in the proxy network.

Table S4 | Proxy records ranked by correlation with target. Proxy network (and metadata) in order of highest absolute correlation with instrumental ERSSTv5 Coral Sea Jan–Mar mean SSTa.

Corr. rank	Series No.	Site name	Proxy	Country	Year from	Year to	Citation	Correlation with Coral Sea SSTa (ERSSTv5)
1	18	Savusavu Bay	Sr/Ca	Fiji	1618	2000	Linsley et al. (2006)	0.59
2	2	Amédée Island	Sr/Ca	New Caledonia	1649	1999	DeLong et al. (2012)	0.52
3	3	Amédée Island	$\delta^{18}O$	New Caledonia	1658	1992	Quinn et al. (1998)	0.51
4	8	Laing Island	$\delta^{18}O$	Papua New Guinea	1884	1993	Tudhope et al. (2001)	0.48
5	12	Nauru	$\delta^{18}O$	Nauru	1897	1995	Guilderson et al. (1999)	0.46
6	22	Vanua Balavu	$\delta^{18}O$	Fiji	1841	2004	Dassié et al. (2014)	0.44
7	19	Savusavu Bay	$\delta^{18}O$	Fiji	1781	1996	Linsley et al. (2006)	0.44
8	17	Savusavu Bay	$\delta^{18}O$	Fiji	1617	2001	Linsley et al. (2006)	0.39
9	20	Savusavu Bay	Sr/Ca	Fiji	1781	1997	Linsley et al. (2006)	0.38
10	5	Flinders Reef	Sr/Ca	Australia	1710	1992	Zinke et al. (2023)	0.31
11	1	Abraham Reef	$\delta^{18}O$	Australia	1638	1983	Druffel & Griffin (1999)	0.30
12	15	Sabine Bank	$\delta^{18}O$	Vanuatu	1842	2007	Gorman et al. (2012)	0.28
13	14	Rabaul	Sr/Ca	Papua New Guinea	1867	1997	Quinn et al. (2006)	0.27
14	9	Madang Lagoon	$\delta^{18}O$	Papua New Guinea	1880	1993	Tudhope et al. (2001)	0.27
15	10	Maiana Atoll	$\delta^{18}O$	Kiribati	1840	1994	Urban et al. (2000)	0.24
16	13	Rabaul	$\delta^{18}O$	Papua New Guinea	1867	1997	Quinn et al. (2006)	0.23
17	16	Savusavu Bay	$\delta^{18}O$	Fiji	1776	2001	Bagnato et al. (2005)	0.22
18	4	Espiritu Santo	$\delta^{18}O$	Vanuatu	1807	1979	Quinn et al. (1996)	0.20
19	11	Nauru	$\delta^{18}O$	Nauru	1891	1995	Guilderson et al. (1999)	0.19
20	7	Kavieng	Sr/Ca	Papua New Guinea	1823	1997	Alibert et al. (2008)	0.12
21	6	Flinders Reef	$\delta^{18}O$	Australia	1710	1992	Zinke et al. (2023)	0.10
22	21	Tarawa Atoll	$\delta^{18}O$	Kiribati	1894	1990	Cole et al. (1993)	0.00

Figure S40 shows the CE, RE, detrended CE and detrended RE of the highest skill reconstruction (maximum time integrated CE) across all 22 proxy networks. All networks produce skilful reconstructions, with CE>0.7 and detrended CE>0.5 for all networks with n_{pert}>1. We conclude that our reconstruction methodology, using existing records, obtains skilful reconstructions and is robust against changes to the network of available data that meet our inclusion criteria.

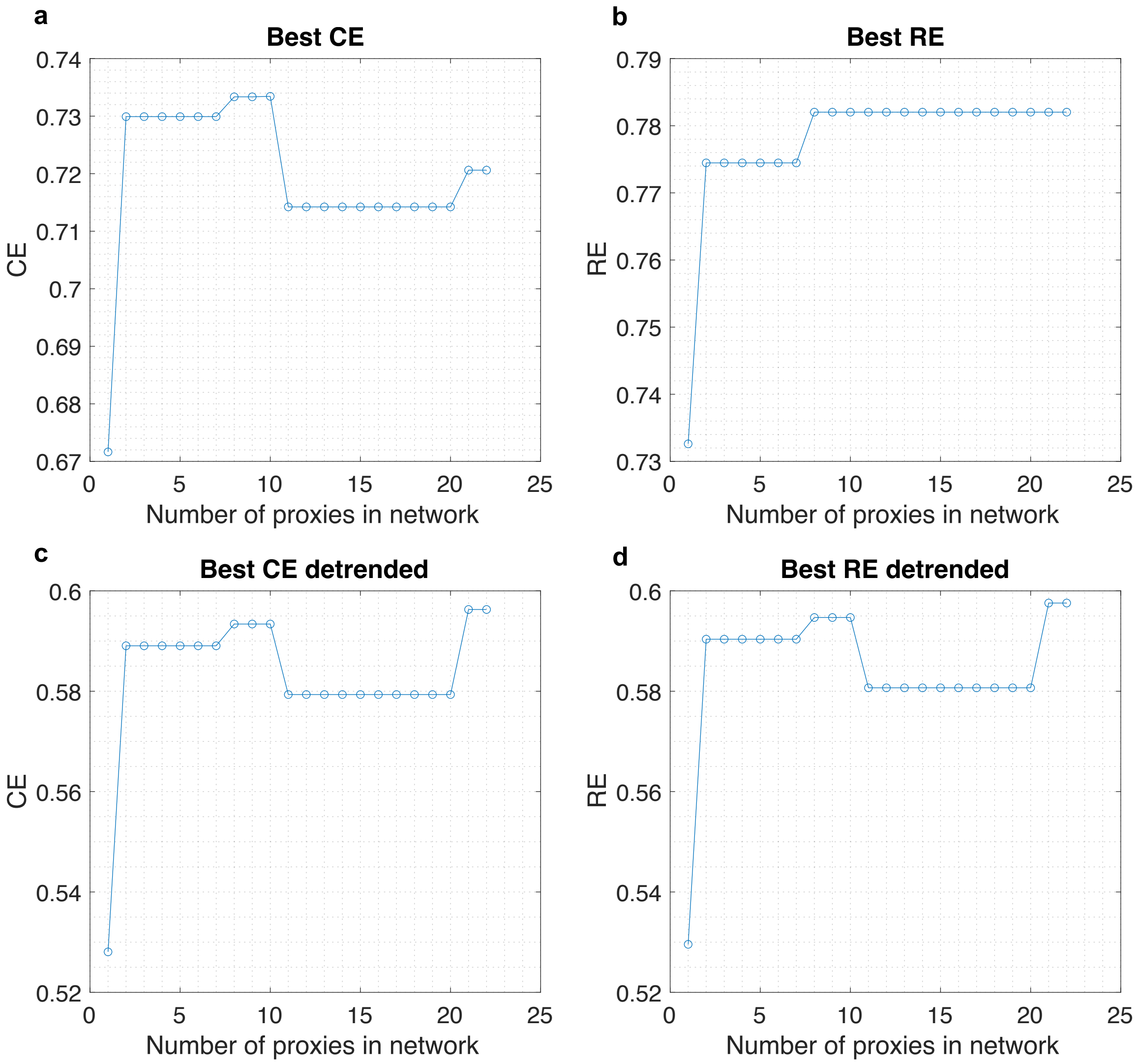

Figure S40 | Skill metrics for network perturbation test. a, CE and **b,** RE of the highest skill reconstruction (maximum time-integrated CE) for each proxy subset in proxy network perturbation test; similarly, **c,** CE and **d,** RE for these highest skill reconstructions but computed after detrending both the calibration and reconstruction series.

Figure S41 shows, for the highest time-integrated CE reconstruction in each of the 22 proxy subsets, the highest pre–1900 reconstructed SSTa [Best estimate (the reconstruction) and its upper 75th, 90th and 95th percentile limits] and the instrumental SSTa in the five recent bleaching years (2016, 2017, 2020, 2022, 2024). The recent thermal maxima all far exceed the best estimate of the highest pre-1900 SSTa in all proxy subsets. The five events are >0.1°C above the 90th percentile limit of all but 2 of the 22 proxy subsets. The 2022 event, the lowest of the four recent events, is approximately equal to the 95th percentile limit of the pre-1900 maximum in the best CE reconstruction from all but 2 of the 22 subsets. The 2024 event is exceptionally warm, at >0.20°C warmer than all of the 95th percentile limits. Results are similar when using HadISST1.1 (Figure S42) instead of ERSSTv5, except that the recent high

SST years have lower anomalies and different rankings in HadISST1.1, and our reconstructed uncertainties are wider, likely associated with the slightly weaker correlations with the coral data. We note that the ERSSTv5 dataset is selected by the Bureau of Meteorology for its GBR regional SST climate monitoring, due to its more comprehensive homogenisation and exclusive use of in-situ data (Bureau of Meteorology, *pers. comm*, 12/09/2023). For the reconstruction calibrated to the HadISST1.1 dataset, the 2016, 2017, 2020 and 2022 extreme SST years still greatly exceed the upper 75th percentile uncertainty limit for all 22 subsets, and for around 15 of the 22 subsets the recent bleaching years exceed the 90th percentile uncertainty limit of the pre-1900 maximum.

We conclude from the network perturbation analysis that for the primary instrumental dataset used in this study (ERSSTv5), and with the data and methodology used here, that the 2017, 2020 and 2024 Jan–Mar thermal extremes in the Coral Sea were (at least) *extremely likely* to be warmer than any other year between 1618–1899 (compared to the 95th percentile across the 22 proxy subsets), and all five events 2016, 2017, 2020, 2022 and 2024 events were (at least) *very likely* to be warmer than any pre–1900 Jan–Mar SSTa in the Coral Sea since at least 1618.

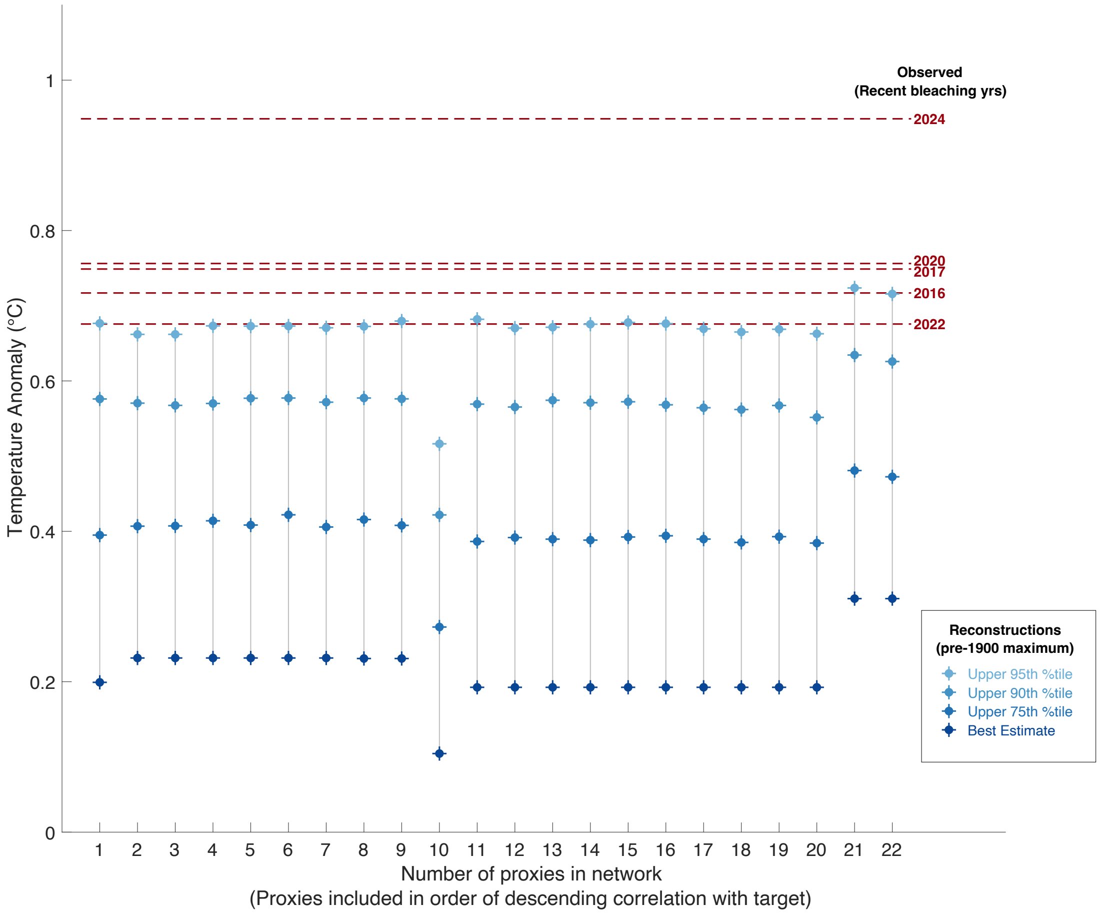

Figure S41 | Recent SSTa extremes (ERSSTv5) relative to reconstructed highest pre-1900 SSTa and percentile limits in network perturbation test. Recent Coral Sea Jan–Mar SSTa during mass bleaching years relative to the reconstructed (calibrated to ERSSTv5) pre-1900 maxima for each of the 22 proxy subsets in the network perturbation test; Blue dots from dark to light show the best estimate (highest CE), 75th, 90th and 95th percentile limits of the reconstructed maxima; SSTa of observed recent high SSTa events is shown with red dotted horizontal lines (2016, 2017, 2020, 2022, 2024).

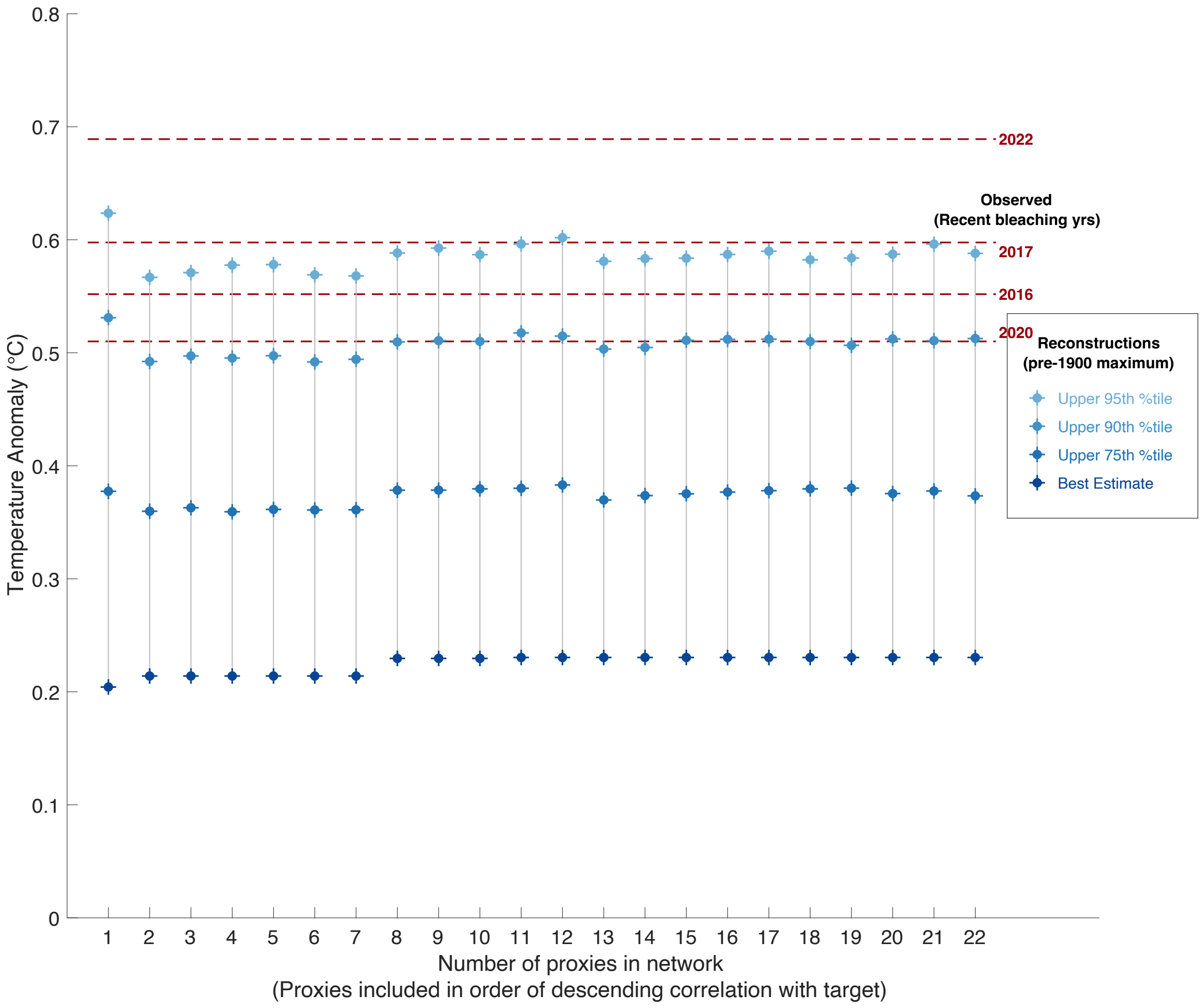

Figure S42 | Recent SSTa extremes (HadISST1.1) relative to reconstructed highest pre-1900 SSTa and percentile limits in network perturbation test. As for Figure S41 but using HadISST1.1. Recent Coral Sea Jan–Mar SSTa during mass bleaching years relative to the reconstructed (calibrated to HadISST1.1) pre-1900 maxima for each of the 22 proxy subsets in the network perturbation test; Blue dots from dark to light show the best estimate (highest CE), 75th, 90th and 95th percentile limits of the reconstructed maxima; SSTa of observed recent high SSTa events is shown with red dotted horizontal lines (2016, 2017, 2020, 2022). for reconstruction calibration and comparison of high SST values during recent mass coral bleaching years. Note that 2024 data for Jan–Mar was not available for HadISST1.1 at the time of writing.

5.2.6. Comparisons across proxy subsets and instrumental datasets

Here we collate the results from the sensitivity tests and compare the results using HadISST1.1 for calibration as well as ERSSTv5. The timeseries of the highest CE reconstructions for each of the five proxy subset sensitivity tests are shown in Figure S43 for ERSSTv5 and Figure S44

for HadISST1.1. As noted previously in relation to Figure S3, there are appreciable differences between the instrumental datasets, particularly for the Coral Sea SST prior to 1950, for which HadISST1.1 tends to be warmer than ERSSTv5. The reconstruction using HadISST1.1 as the target results in a more muted centennial trend, with the 1700–1900 period having a mean SSTa of around -0.25°C, rather than around -0.5°C for ERSSTv5. Since the HadISST1.1 dataset has lower correlations with the proxy network (see Figure S9), its reconstructions have lower evaluation metrics, and advice from the Bureau of Meteorology suggests that ERSSTv5 has more comprehensive homogenisation than HadISST, we have presented the reconstruction with ERSSTv5 in the main text. We note however that the key result, that the recent mass bleaching years are above the best estimate of the pre-1900 maximum, is evident for reconstructions based on both HadISST1.1 and ERSSTv5. Mass bleaching in the context of more muted recent warming relative to past centuries could be concerning as it may point to the GBR corals having a higher sensitivity to changes in temperature.

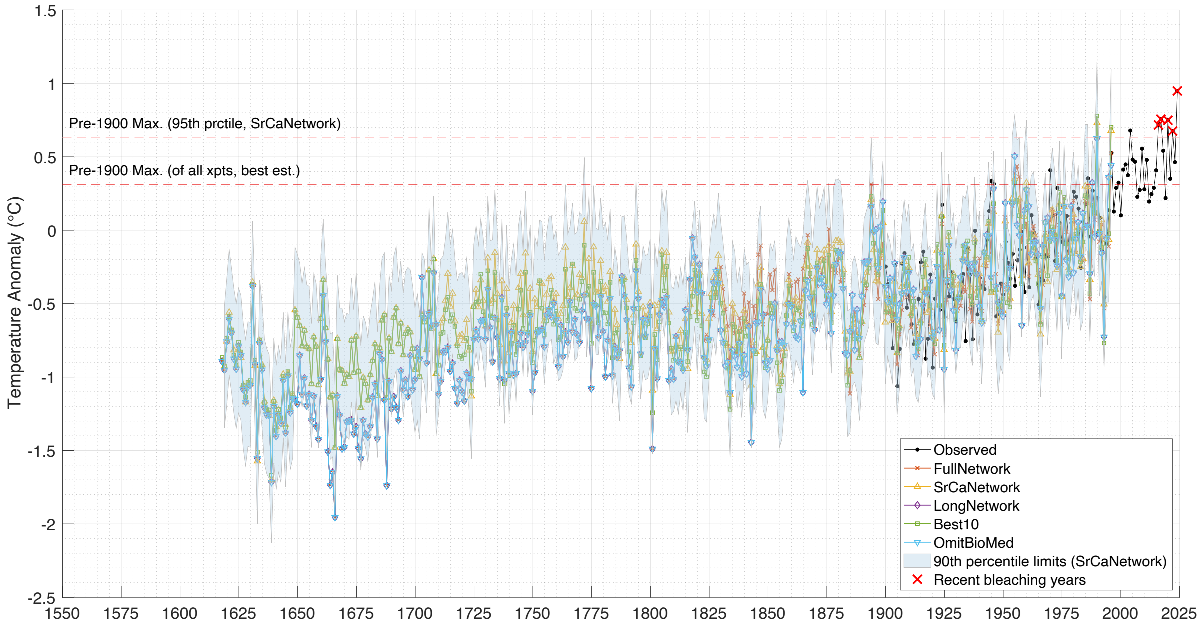

Figure S43 | Reconstructed Coral Sea SSTa for five proxy subsets calibrated to ERSSTv5 (2017–2024). Best estimate (highest CE) reconstructions for the five proxy subsets; 90th percentile limit uncertainty bounds for the Sr/Ca-only network. Horizontal red lines show pre-1900 maximum of the best estimate of all subsets and the 95th percentile limit for the pre-1900 maximum for the Sr/Ca-only network. Correlations between each of the reconstructions for the proxy subsets are significant at the 5% significance level ($\rho>0.87$, $p<0.01$).

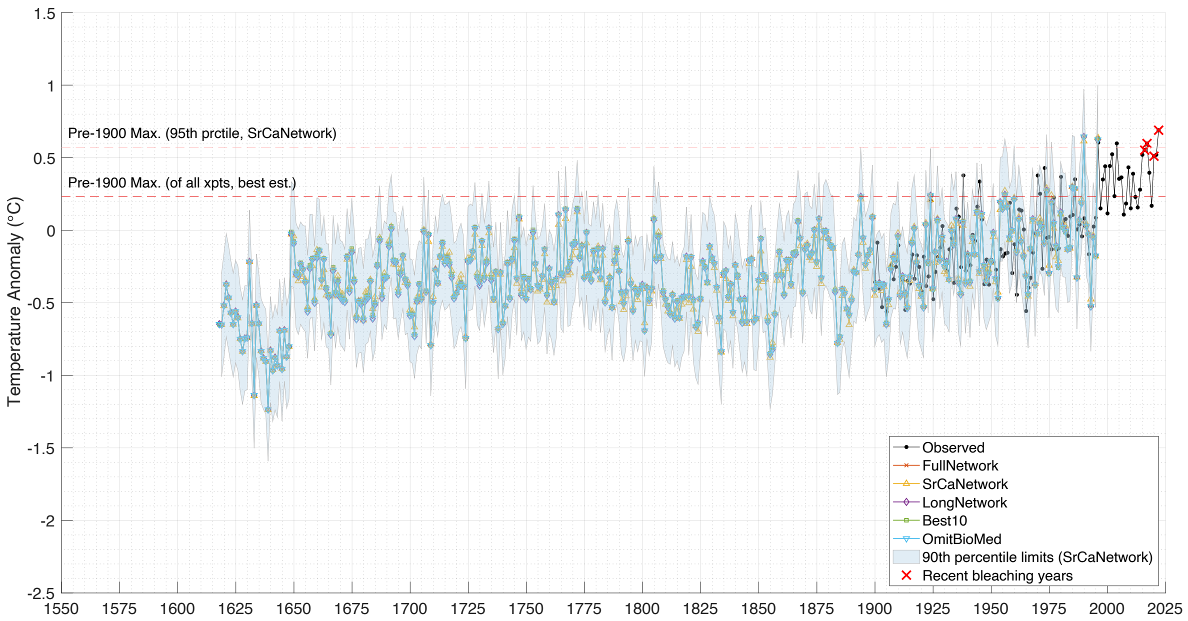

Figure S44 | Reconstructed Coral Sea SSTa for five proxy subsets calibrated to HadISST1.1 (2017–2023). Similarly to Figure S45 but for HadISST1.1, best estimate (highest CE) reconstructions for the five proxy subsets; 90th percentile limit uncertainty bounds for the Sr/Ca-only network. Horizontal red lines show pre-1900 maximum of the best estimate of all subsets and the 95th percentile limit for the pre-1900 maximum for the Sr/Ca-only network. Correlations between each of the reconstructions for the proxy subsets are significant at the 5% significance level ($\rho>0.95$, $p<0.01$). Note 2024 Jan–Mar data was not available for HadISST1.1 at the time of writing.

5.2.7. Sensitivity to averaging window of sub-annual coral records

For the main results of this study (shown in Figs. 2 and 3), we averaged the coral proxy data over the period Nov–Apr to account for possible dating inaccuracies at the monthly timestep and the possibility of post depositional smearing of the summer signal from bio-smoothing. Here we undertake a reconstruction with the full network of all 22 proxies using a narrower (Jan–Mar) window for the proxy averaging step, precisely matching the reconstruction target window. The reconstruction remains skilful (Fig. S46c), but not quite as skilful as the previous result which used the Nov–Apr window (Fig 2c in the main text). The recent Coral Sea SSTa for the five mass coral bleaching years exceed both the 90th and 95th uncertainty limits of the pre-1900 maximum (Fig. S47a), so the key conclusions of this study are again confirmed.

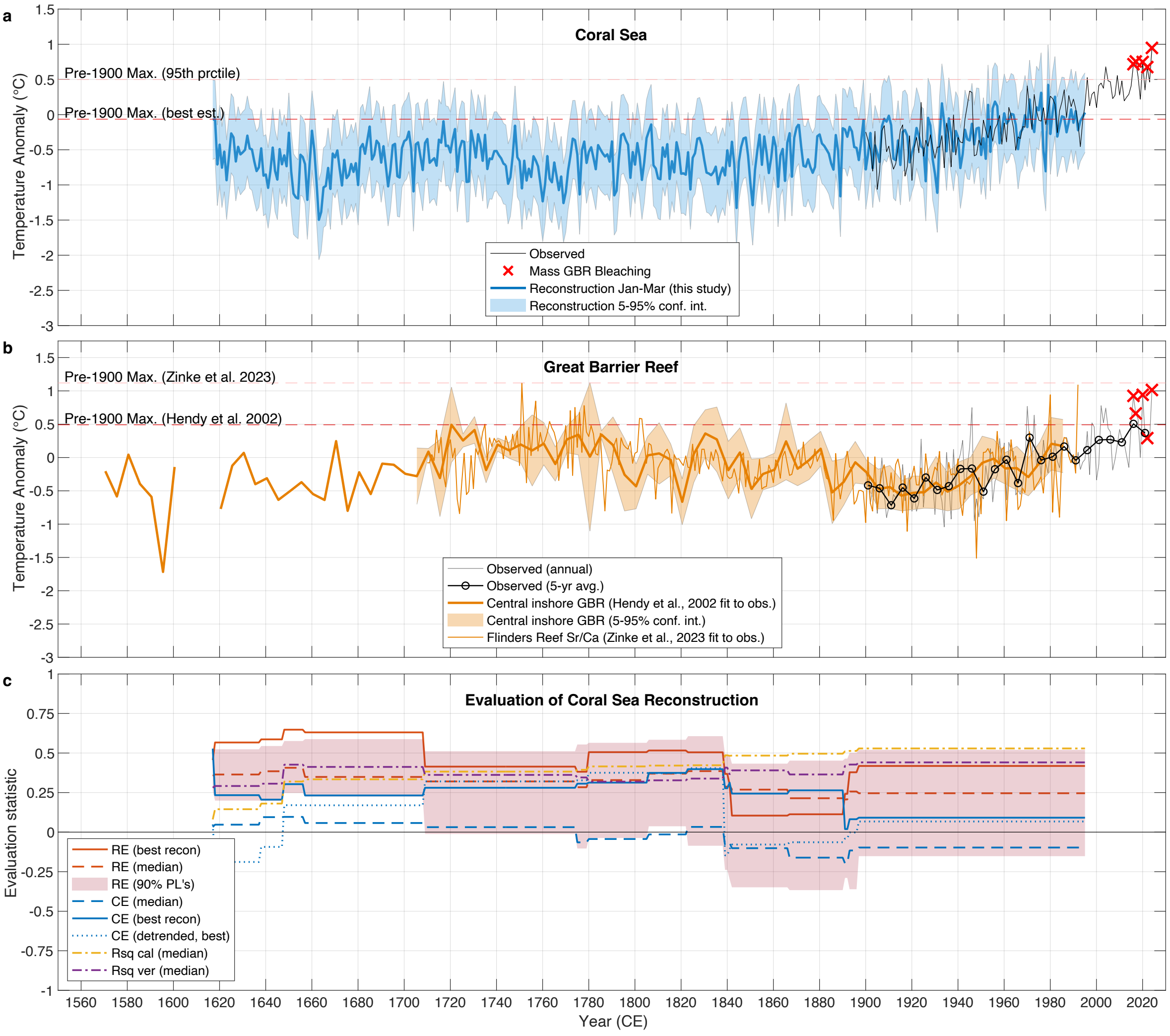

Figure S48 | Multi-century reconstruction of sea surface temperatures using Jan–Mar as the proxy averaging period (ERSSTv5). As for Fig 2 a–c in the main paper but using a Jan–Mar proxy averaging period for the sub-annually resolved coral data rather than Nov–Apr (and the map in panel d omitted). **a,** Coral Sea reconstructed and observed mean sea surface temperature anomalies, relative to 1961–1990, for the period 1618–2024; highest skill (maximum CE) reconstruction with the full proxy network shown in dark blue; 5–95th percentile reconstruction uncertainty shown in lighter blue shading; observed (ERSSTv5) data shown in black; recent five mass bleaching events shown with red crosses; dotted horizontal lines indicate the best estimate (highest skill) and 95th percentile uncertainty bound for the maximum pre-1900 SSTa; **b,** Central Great Barrier Reef sea surface temperature series in thick and thin orange lines aligned with modern observations shown at annual (thin grey line) and 5-yr resolution (black line and open circles); dotted horizontal lines indicate best estimate pre-1900 maxima; **c,** Coral Sea reconstruction evaluation metrics (Supplementary Section 3.1);

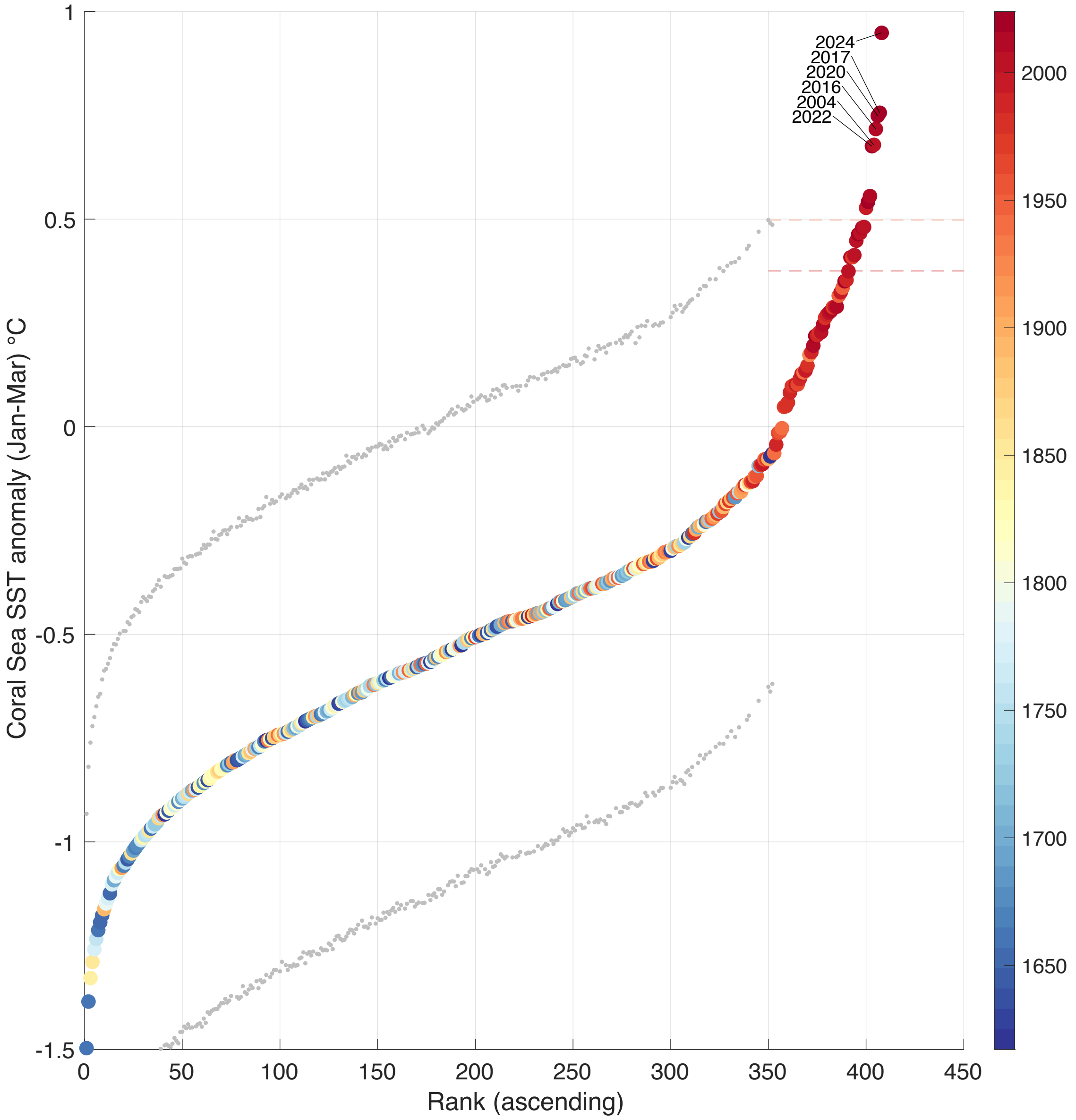

Figure S49 | Exceptional nature of recent Coral Sea surface temperatures, using Jan–Mar proxy averaging period (ERSSTv5). As for Fig 3 in the main paper but using a Jan–Mar proxy averaging period for the sub-annually resolved coral data. Ranked sea surface temperature anomalies, relative to 1961–1990 (coloured circles) for Jan–Mar for 1618–2024 for the best estimate (highest skill, full coral network) reconstruction (1618-1899) and instrumental (ERSSTv5) data (1900–2024); year indicated by colour of filled circles; 5–95th uncertainty in pre-1900 reconstructed temperature anomalies shown with small grey dots; year labels indicate warmest six years on record, five of which were mass coral bleaching events on the GBR; light red (upper) dashed line indicates the upper 95th percentile uncertainty bound of the maximum pre-1900 reconstructed sea surface temperature anomaly; dark red (lower) dashed line indicates the 90th percentile limit.

6. Climate model attribution with CMIP6 models

6.1. Model runs available and transient climate responses

Table S5 shows the climate model runs and the Transient Climate Responses (TCR) of each model, as reported by Ref [31].

Table S5 | CMIP6 model runs summary; TCR estimates from Ref [31].

No	CMIP6 Model	Number of Hist runs available	Number of HistNat runs available	TCR (°C)	TCR in likely range? (1.4–2.2°C)
1	ACCESS-CM2	3	3	1.96	Y
2	ACCESS-ESM1-5	30	3	1.97	Y
3	BCC-CSM2-MR	3	3	1.55	Y
4	CESM2	11	3	2.00	Y
5	CNRM-CM6-1	28	10	2.22	N
6	CanESM5	50	28	2.71	N
7	FGOALS-g3	6	3	1.50	Y
8	GFDL-ESM4	3	3	1.63	Y
9	GISS-E2-1-G	39	15	1.80	Y
10	HadGEM3-GC31-LL	4	4	2.49	N
11	IPSL-CM6A-LR	32	10	2.35	N
12	MIROC6	50	3	1.55	Y
13	MRI-ESM2-0	6	4	1.67	Y
14	NorESM2-LM	3	3	1.49	Y

6.2. Signal and noise for TCR-likely, equal numbers of model ensemble members

Figure S50 shows timeseries of the signal, noise and signal-to-noise ratio of the Coral Sea Jan–Mar SSTa for the CMIP6 models which are within the likely range (1.4–2.2°C) for their TCR (hereafter "TCR likely") based on Ref [31] (see Methods).

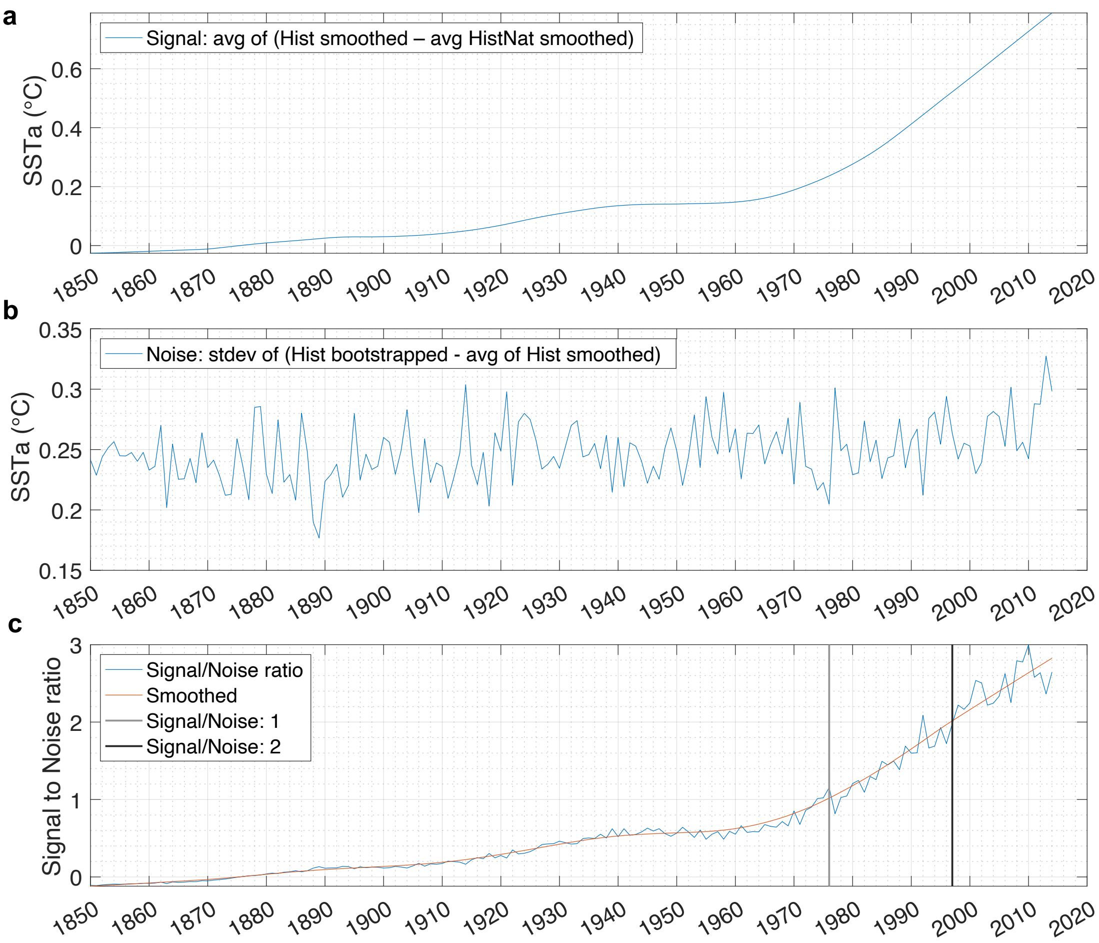

Figure S51 | Climate model simulations of the climate change signal and noise in Coral Sea surface temperatures. a, Signal, **b,** noise and **c,** signal-to-noise ratio of CMIP6 TCR-likely models as shown in Figure 4 of the main text, bootstrap sampled with equal numbers of ensemble members (3) per model. The anthropogenic 'signal' is the mean of the difference between the smoothed (using a 41-yr Lowess filter) modelled historical Coral Sea SSTa and the mean smoothed modelled historical natural SSTa. The "noise" is the standard deviation of the residuals of the modelled historical SSTa from its smoothed timeseries. The ratio of these two values is computed for each year (Jan–Mar season). The signal-to-noise ratio exceeds 1 and 2 in 1976 and 1997, respectively.

6.3. Sensitivity of time of emergence to model subset and ensemble numbers

Here we test the sensitivity of the time of emergence, as estimated by the signal-to-noise ratio method from ref [32], to the model subset and the availability of differing numbers of ensemble members across the models. In the main text, we use the TCR-likely subset of models (those with a 'Y' in column 6 of Table S5, as determined by ref [31], the supplementary data from which is located here). The four models excluded from the TCR-likely subset are: CNRM-CM6-1, CanESM5, HadGEM3-GC31-LL, IPSL-CM6A-LR. The remaining 10 TCR likely models in the subset are: ACCESS-CM2, ACCESS-ESM1-5, BCC-CSM2-MR, CESM2, FGOALS-g3, GFDL-ESM4, GISS-E2-1-G, MIROC6, MRI-ESM2-0, NorESM2-LM. We also ensure that each remaining model has equal representation in our analysis using a bootstrapping approach which samples 3 ensemble members from each model (the minimum number of ensemble members from any model in our subset). We undertake two additional sensitivity tests here, using:

a) All models and all available ensemble members (unequal model weighting because different models have different numbers of ensemble members): the model ensemble is taken as-is, with no subsampling of the ensemble members.
b) All models and an equal number of ensemble members (equal model weighting): we use a bootstrapping approach to sample an equal number of ensemble members for each model.

The sensitivity tests are intended to explore the influence of the model subsets and the equal/unequal representation of models, via their numbers of ensemble members, on our analysis. The results from both tests (with all models) show more rapid rate of historical warming ('signal') in the latter 20th century, but also a wider 5–95th percentile ('noise') range. The net effect is that the signal-to-noise (S/N) ratio reaches 1.0 and 2.0 within 3–4 years of the TCR-likely subset analysis, highlighting the robustness of the approach.

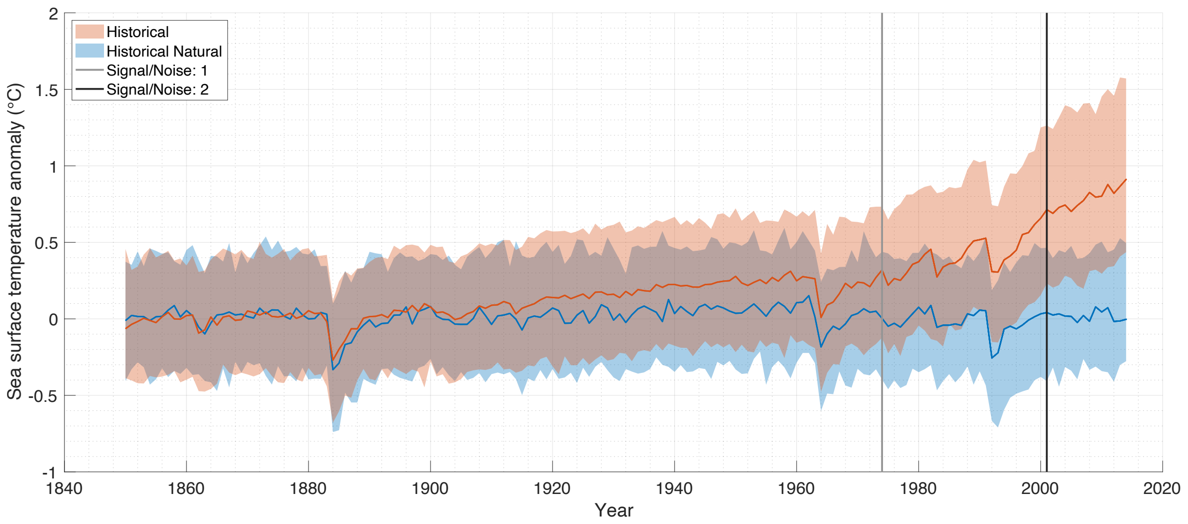

Figure S52 | Climate change is driving the rise in Coral Sea surface temperatures, using all models and ensemble members. As for Fig 4 from main text, but for sensitivity test (a), all models, all ensemble members. Climate model simulations of Coral Sea Jan–Mar sea surface temperature anomalies relative to the 1850–1900 average for the period 1850–2014, for all available model simulations; Blue line (median) and light blue shading (5–95th percentiles) are from the 'historical natural' climate model simulations (no anthropogenic forcing is included); Red line and light red shading is from the 'historical' simulations from the same set of climate models, but *with* anthropogenic influences on the climate included. The climate model-derived time of emergence of anthropogenic climate change is shown in the grey and black vertical lines when the ratio of the climate change signal to the standard deviation of noise/variability across model ensemble members first rises above one and two respectively.

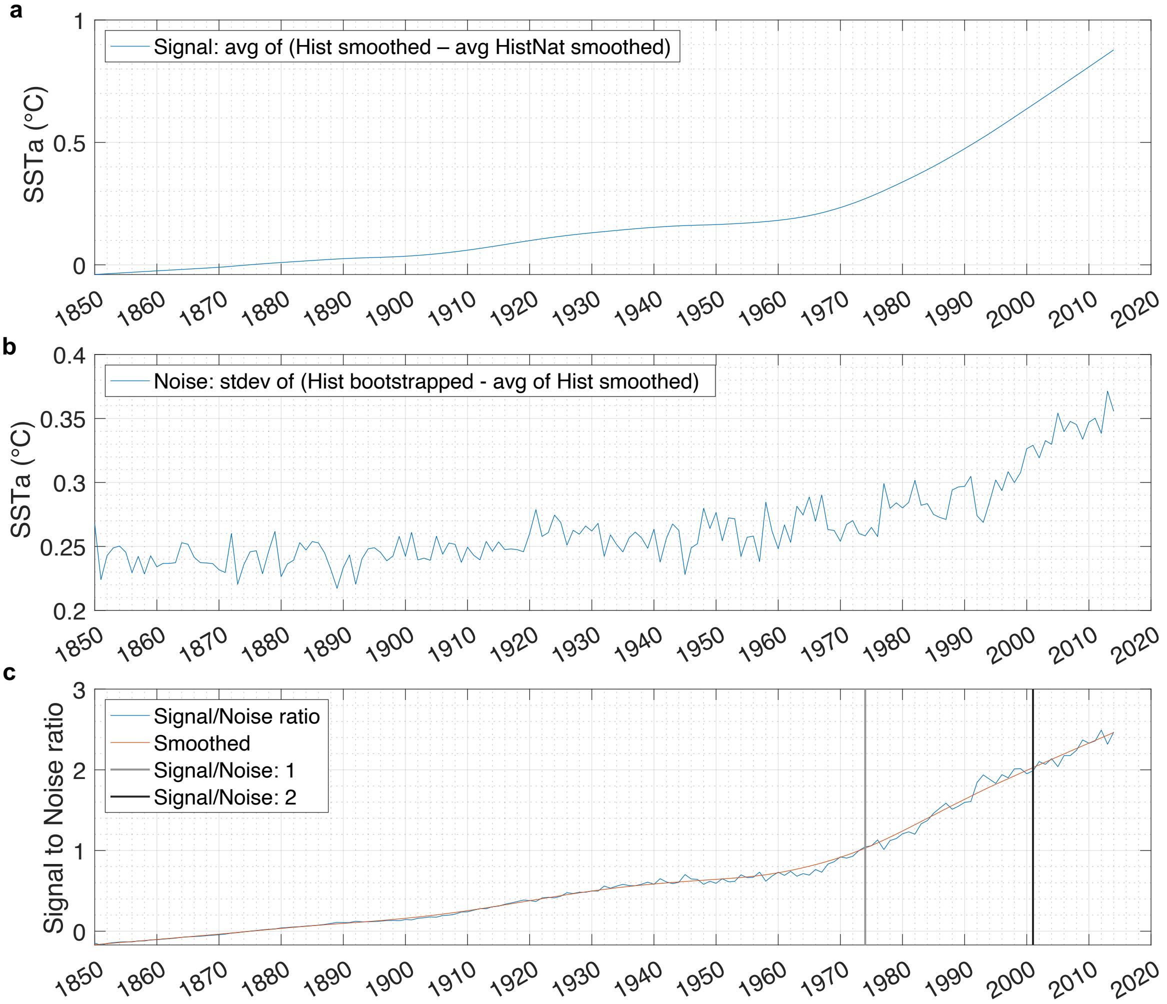

Figure S53 | Climate model simulations of the climate change signal and noise in Coral Sea surface temperatures for all models and ensemble members. a, Signal, **b,** noise and **c,** signal-to-noise ratio of all available model simulations. The anthropogenic 'signal' is the mean of the difference between the smoothed (using a 41-yr Lowess filter) modelled historical Coral Sea SSTa and the mean smoothed modelled historical natural SSTa. The "noise" is the standard deviation of the residuals of the modelled historical SSTa from its smoothed timeseries. The ratio of these two values is computed for each year (Jan–Mar season).

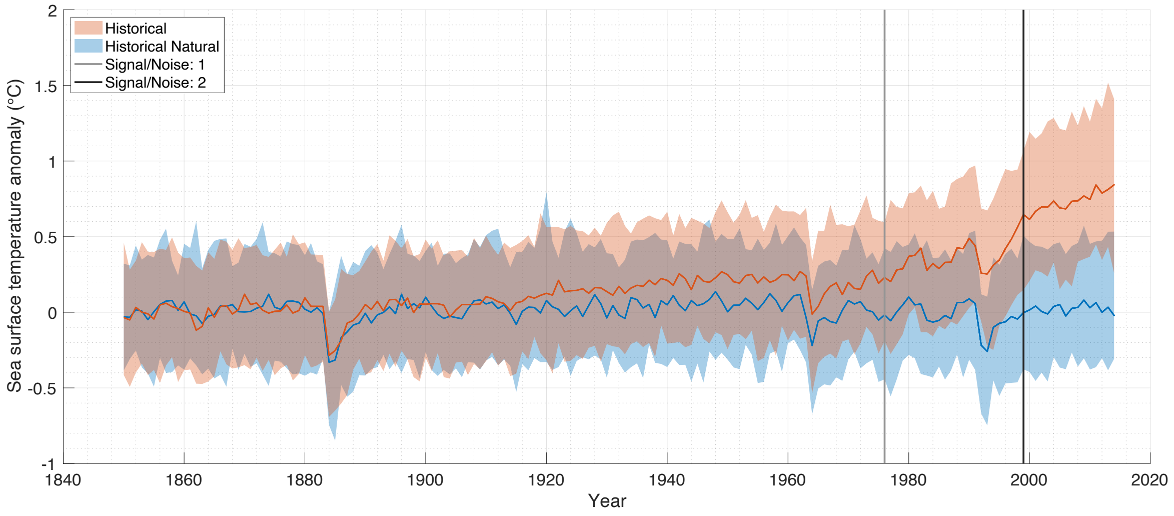

Figure S54 | Climate change is driving the rise in Coral Sea surface temperatures, using all models with equal number of ensemble members. As for Fig 4 from main paper, but for sensitivity test (b), all available models but an equal number of ensemble members per model. Climate model simulations of Coral Sea Jan–Mar sea surface temperature anomalies relative to the 1850–1900 average for the period 1850–2014, for all available models but bootstrapped to have equal number of simulations per model (3); Blue line (median) and light blue shading (5–95th percentiles) are from the 'historical natural' climate model simulations (no anthropogenic forcing is included); Red line and light red shading is from the 'historical' simulations from the same set of climate models, but *with* anthropogenic influences on the climate included. The climate model-derived time of emergence of anthropogenic climate change is shown in the grey and black vertical lines when the ratio of the climate change signal to the standard deviation of noise/variability across model ensemble members first rises above one and two respectively.

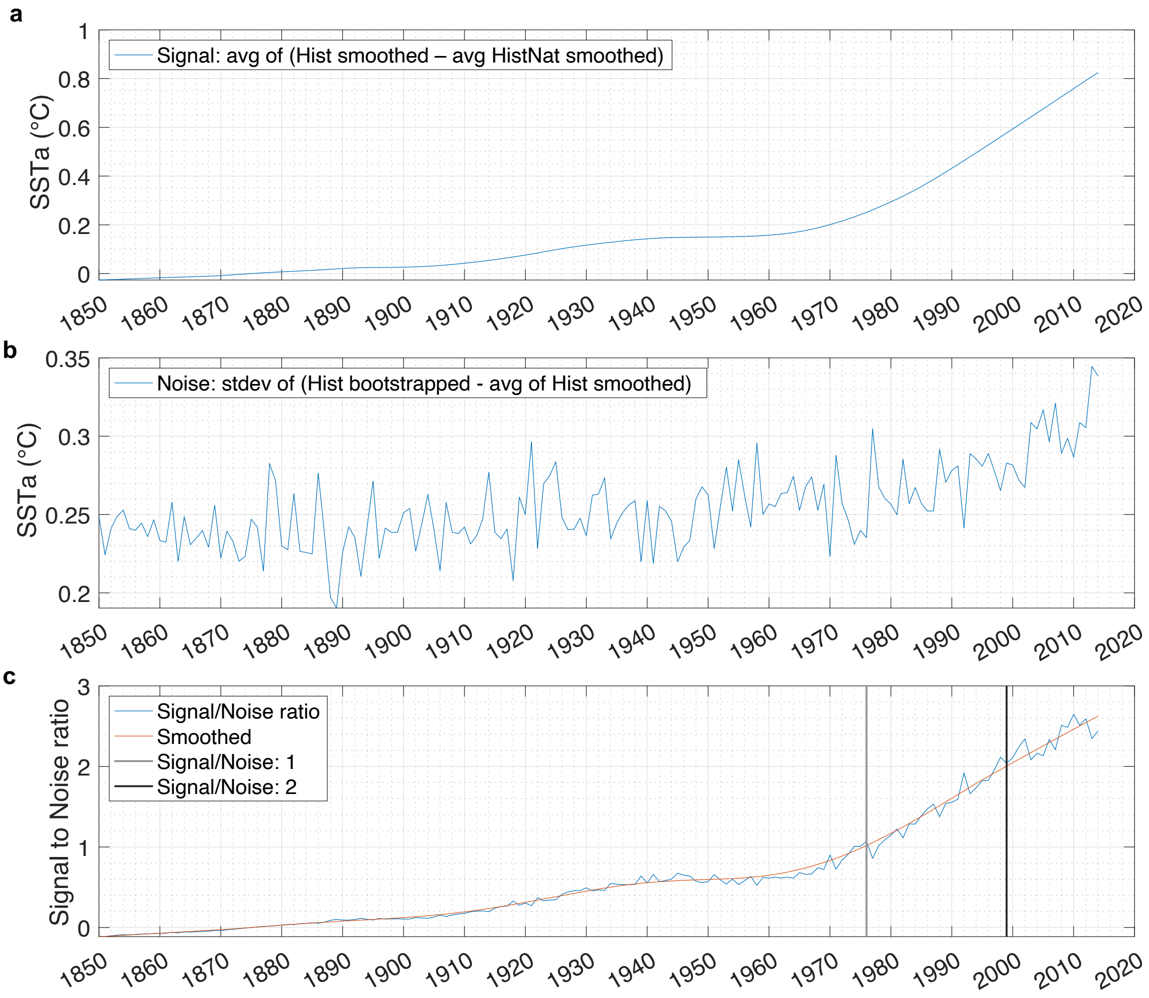

Figure S55 | Climate model simulations of the climate change signal and noise in Coral Sea surface temperatures for all models and equal numbers of ensemble members. As for Figure S51, but for but for sensitivity test (b), all models with an equal number of ensemble members per model. **a,** Signal, **b,** noise and **c,** signal-to-noise ratios. The anthropogenic 'signal' is the mean of the difference between the smoothed (using a 41-yr Lowess filter) modelled historical Coral Sea SSTa and the mean smoothed modelled historical natural SSTa. The "noise" is the standard deviation of the residuals of the modelled historical SSTa from its smoothed timeseries. The ratio of these two values is computed for each year (Jan–Mar season).

Supplementary References

71. Freeman, E. *et al.* ICOADS Release 3.0: a major update to the historical marine climate record. *International Journal of Climatology* **37**, 2211–2232 (2017).

72. Huang, B. *et al.* Uncertainty estimates for sea surface temperature and land surface air temperature in NOAAGlobalTemp version 5. *J Clim* **33**, 1351–1379 (2020).

73. Druffel, E. R. M. & Griffin, S. Variability of surface ocean radiocarbon and stable isotopes in the southwestern Pacific. *J Geophys Res* **104**, 23607–23613 (1999).

74. DeLong, K. L., Quinn, T. M., Taylor, F. W., Lin, K. & Shen, C.-C. Sea surface temperature variability in the southwest tropical Pacific since AD 1649. *Nat Clim Chang* **2**, 799–804 (2012).

75. Quinn, T. *et al.* A multicentury stable isotope record from a New Caledonia coral: Interannual and decadal SST variability in the southwest Pacific since 1657. *Paleoceanography* **13**, 412–426 (1998).

76. Quinn, T. M., Crowley, T. J. & Taylor, F. W. New stable isotope results from a 173-year coral from Espiritu Santo, Vanuatu. *Geophys Res Lett* **23**, 3413–3416 (1996).

77. Alibert, C. & Kinsley, L. A 170-year Sr/Ca and Ba/Ca coral record from the western Pacific warm pool: 1. What can we learn from an unusual coral record? *J Geophys Res Oceans* **113**, C04008 (2008).

78. Tudhope, A. W. *et al.* Variability in the El Niño-Southern Oscillation through a glacial-interglacial cycle. *Science* **291**, 1511–1517 (2001).

79. Urban, F. E., Cole, J. E. & Overpeck, J. T. Influence of mean climate change on climate variability from a 155-year tropical Pacific coral record. *Nature* **407**, 989–993 (2000).

80. Guilderson, T. P. & Schrag, D. P. Reliability of coral isotope records from the western Pacific warm pool: A comparison using age-optimized records. *Paleoceanography* **14**, 457–464 (1999).

81. Quinn, T. M., Taylor, F. W. & Crowley, T. J. Coral-based climate variability in the Western Pacific Warm Pool since 1867. *J Geophys Res* **111**, C11006 (2006).

82. Gorman, M. K. *et al.* A coral-based reconstruction of sea surface salinity at Sabine Bank, Vanuatu from 1842 to 2007 CE. *Paleoceanography* **27**, PA3226 (2012).

83. Bagnato, S., Linsley, B. K., Howe, S. S., Wellington, G. M. & Salinger, J. Coral oxygen isotope records of interdecadal climate variations in the South Pacific Convergence Zone region. *Geochemistry, Geophysics, Geosystems* **6**, Q06001 (2005).

84. Linsley, B. K. *et al.* Tracking the extent of the South Pacific Convergence Zone since the early 1600s. *Geochemistry Geophysics Geosystems* **7**, Q05003 (2006).

85. Cole, J. E., Fairbanks, R. G. & Shen, G. T. Recent Variability in the Southern Oscillation: Isotopic Results from a Tarawa Atoll Coral. *Science* **260**, 1790–1793 (1993).

86. Dassié, E. P. *et al.* A Fiji multi-coral δ18O composite approach to obtaining a more accurate reconstruction of the last two-centuries of the ocean-climate variability in the South Pacific Convergence Zone region. *Paleoceanography* **29**, 1196–1213 (2014).

87. Carton, J. A., Chepurin, G. A. & Chen, L. SODA3: A New Ocean Climate Reanalysis. *J Clim* **31**, 6967–6983 (2018).

88. Zuo, H., Balmaseda, M. A., Tietsche, S., Mogensen, K. & Mayer, M. The ECMWF operational ensemble reanalysis-analysis system for ocean and sea ice: A description of the system and assessment. *Ocean Science* **15**, 779–808 (2019).

89. Cheng, L. *et al.* Improved Estimates of Changes in Upper Ocean Salinity and the Hydrological Cycle. *J Clim* **33**, 10357–10381 (2020).

90. Thompson, D. M. *et al.* Identifying Hydro-Sensitive Coral δ 18O Records for Improved High-Resolution Temperature and Salinity Reconstructions. *Geophys Res Lett* **49**, e2021GL096153 (2022).

91. Wu, Y., Fallon, S. J., Cantin, N. E. & Lough, J. M. Assessing multiproxy approaches (Sr/Ca, U/Ca, Li/Mg, and B/Mg) to reconstruct sea surface temperature from coral skeletons throughout the Great Barrier Reef. *Science of the Total Environment* **786**, 147393 (2021).

92. Sadler, J., Webb, G. E., Leonard, N. D., Nothdurft, L. D. & Clark, T. R. Reef core insights into mid-Holocene water temperatures of the southern Great Barrier Reef. *Paleoceanography* **31**, 1395–1408 (2016).

93. Roche, R. C. *et al.* Mid-Holocene sea surface conditions and riverine influence on the inshore Great Barrier Reef. *Holocene* **24**, 885–897 (2014).

94. Reed, E. V, Cole, J. E., Lough, J. M., Thompson, D. & Cantin, N. E. Linking climate variability and growth in coral skeletal records from the Great Barrier Reef. *Coral Reefs* **38**, 29–43 (2019).

95. Razak, T. B. *et al.* Use of skeletal Sr/Ca ratios to determine growth patterns in a branching coral Isopora palifera. *Mar Biol* **164**, 96 (2017).

96. Marshall, J. F. Decadal-scale, high resolution records of sea surface temperature in the eastern Indian and south western Pacific Oceans from proxy records of the strontium/calcium ratio of massive porites corals. vol. PhD (Australian National University, 2000).

97. Marshall, J. F. & McCulloch, M. T. An assessment of the Sr/Ca ratio in shallow water hermatypic corals as a proxy for sea surface temperature. *Geochim Cosmochim Acta* **66**, 3263–3280 (2002).

98. Gagan, M. K. *et al.* Coral oxygen isotope evidence for recent groundwater fluxes to the Australian Great Barrier Reef. *Geophys Res Lett* **29**, 43–1–43–4 (2002).

99. D'Olivo, J. P., Sinclair, D. J., Rankenburg, K. & McCulloch, M. T. A universal multi-trace element calibration for reconstructing sea surface temperatures from long-lived Porites corals: Removing 'vital-effects'. *Geochim Cosmochim Acta* **239**, 109–135 (2018).

100. Fallon, S. J., McCulloch, M. T. & Alibert, C. Examining water temperature proxies in Porites corals from the Great Barrier Reef: a cross-shelf comparison. *Coral Reefs* **22**, 389–404 (2003).

101. Brenner, L. D., Linsley, B. K. & Potts, D. C. A modern Sr/Ca-δ18O-sea surface temperature calibration for Isopora corals on the Great Barrier Reef. *Paleoceanography* **32**, 182–194 (2017).

102. Alibert, C. *et al.* Source of trace element variability in Great Barrier Reef corals affected by the Burdekin flood plumes. *Geochim Cosmochim Acta* **67**, 231–246 (2003).

103. Murty, S. A. *et al.* Spatial and Temporal Robustness of Sr/Ca-SST Calibrations in Red Sea Corals: Evidence for Influence of Mean Annual Temperature on Calibration Slopes. *Paleoceanogr Paleoclimatol* **33**, 443–456 (2018).

104. Sayani, H. R., Cobb, K. M., DeLong, K., Hitt, N. T. & Druffel, E. R. M. Intercolony δ18O and Sr/Ca variability among Porites spp. corals at Palmyra Atoll: Toward more robust coral-based estimates of climate. *Geochemistry, Geophysics, Geosystems* **20**, 5270–5284 (2019).

105. Otto, F. E. L. Geert Jan van Oldenborgh 1961–2021. *Nat Clim Chang* **11**, 1017–1017 (2021).
